# Molecular analyses of zebrafish V0v spinal interneurons and identification of transcriptional regulators downstream of Evx1 and Evx2 in these cells

**DOI:** 10.1186/s13064-023-00176-w

**Published:** 2023-11-28

**Authors:** Samantha J. England, Amber K. Rusnock, Amra Mujcic, Angelica Kowalchuk, Sarah de Jager, William C. Hilinski, José L. Juárez-Morales, Matthew E. Smith, Ginny Grieb, Santanu Banerjee, Katharine E. Lewis

**Affiliations:** 1 Biology Department, Syracuse University, Syracuse, NY USA; 2 Physiology, Development and Neuroscience Department, Cambridge University, Cambridge, UK; 3 Programa de IxM-CONAHCYT, Centro de Investigaciones Biológicas del Noroeste, S.C. (CIBNOR), La Paz, Baja California Sur, México; 4 Biological Sciences Department, SUNY-Cortland, Cortland, NY USA

**Keywords:** V0 interneurons, Spinal cord, Zebrafish, scRNA-seq, V1 cells, Hmx3, Skor, Neuronal intermediate filament (NIF), Glutamatergic, Gene regulatory network (GRN)

## Abstract

**Background:**

V0v spinal interneurons are highly conserved, glutamatergic, commissural neurons that function in locomotor circuits. We have previously shown that Evx1 and Evx2 are required to specify the neurotransmitter phenotype of these cells. However, we still know very little about the gene regulatory networks that act downstream of these transcription factors in V0v cells.

**Methods:**

To identify candidate members of V0v gene regulatory networks, we FAC-sorted wild-type and *evx1;evx2* double mutant zebrafish V0v spinal interneurons and expression-profiled them using microarrays and single cell RNA-seq. We also used in situ hybridization to compare expression of a subset of candidate genes in *evx1;evx2* double mutants and wild-type siblings.

**Results:**

Our data reveal two molecularly distinct subtypes of zebrafish V0v spinal interneurons at 48 h and suggest that, by this stage of development, *evx1;evx2* double mutant cells transfate into either inhibitory spinal interneurons, or motoneurons. Our results also identify 25 transcriptional regulator genes that require Evx1/2 for their expression in V0v interneurons, plus a further 11 transcriptional regulator genes that are repressed in V0v interneurons by Evx1/2. Two of the latter genes are *hmx2* and *hmx3a*. Intriguingly, we show that Hmx2/3a, repress dI2 interneuron expression of *skor1a* and *nefma*, two genes that require Evx1/2 for their expression in V0v interneurons. This suggests that Evx1/2 might regulate *skor1a* and *nefma* expression in V0v interneurons by repressing Hmx2/3a expression.

**Conclusions:**

This study identifies two molecularly distinct subsets of zebrafish V0v spinal interneurons, as well as multiple transcriptional regulators that are strong candidates for acting downstream of Evx1/2 to specify the essential functional characteristics of these cells. Our data further suggest that in the absence of both Evx1 and Evx2, V0v spinal interneurons initially change their neurotransmitter phenotypes from excitatory to inhibitory and then, later, start to express markers of distinct types of inhibitory spinal interneurons, or motoneurons. Taken together, our findings significantly increase our knowledge of V0v and spinal development and move us closer towards the essential goal of identifying the complete gene regulatory networks that specify this crucial cell type.

**Supplementary Information:**

The online version contains supplementary material available at 10.1186/s13064-023-00176-w.

## Background

For the Central Nervous System (CNS) to operate correctly, neurons with appropriate functions need to be precisely created and accurately connected into circuits. However, we still do not understand how this vital aspect of neural development is achieved. The spinal cord is a powerful system for establishing fundamental principles of neuronal fate specification and circuit assembly, as it is relatively simple and experimentally tractable compared to the brain. It is also an essential part of the CNS as the spinal cord controls locomotion and receives and processes sensory information from the trunk and limbs. In addition, spinal cord dysfunction caused by abnormal development, injury or disease can profoundly impair quality of life. Therefore, it is essential that we better understand neuronal specification in the spinal cord, so that we can develop more effective therapies to treat these debilitating conditions.

To elucidate how spinal cord circuitry develops, we first need to establish how neuronal functional properties are specified, as these properties determine the circuits that specific neurons participate in and their functions within those circuits. Most of the neurons in the spinal cord are interneurons, so called because their cell bodies and axons reside entirely within the CNS. Interneurons have essential roles within most spinal circuits. One of the most important functional properties that helps to define distinct interneurons, and their specific functions in neural circuitry, is which neurotransmitter they use to communicate with other cells. Spinal interneurons use three major neurotransmitters: glutamate, which is excitatory; glycine, which is inhibitory; and GABA, which, with a few exceptions at particular stages of development, is usually inhibitory. If these neurotransmitter phenotypes are wrongly specified then the affected interneurons will inappropriately inhibit rather than excite, or vice versa, their synaptic partners within neuronal circuits, and the functional outputs and behaviors of those circuits will be dramatically disturbed, usually with pathological consequences [1].

All of the data so far, suggest that neurotransmitter phenotypes, and other aspects of cell fate specification are determined by the transcription factors that an interneuron expresses when it becomes post-mitotic and starts to differentiate (e.g. [2–7]). In most cases analyzed so far, several transcription factors act together, or in succession, as part of a Gene Regulatory Network (GRN) that specifies a particular functional property (e.g. [3–6]). We already know several transcription factors that are part of GRNs that specify spinal interneuron inhibitory [4–10] or excitatory [4, 8, 11–14] phenotypes. However, there are still fundamental gaps in our knowledge of how spinal interneuron neurotransmitter phenotypes are specified and maintained. For example, it is very unlikely that we have identified all the transcription factors that play crucial roles in the GRNs in any specific class of spinal interneurons, or all their epistatic relationships.

In this study, we concentrate on V0v spinal interneurons. These are highly conserved glutamatergic, commissural neurons that exist in all vertebrates examined so far. V0v spinal interneurons are located in the middle of the dorsal/ventral axis of the spinal cord, and they are required for correct left–right alternation during fast locomotion [15–26]. Given their important role in locomotion, it is vital that we understand how V0v interneurons are specified, and, in particular, how they adopt their excitatory neurotransmitter fate. Within both the mouse and zebrafish spinal cord, two highly related transcription factors, Evx1 and Evx2 (Evx1/2), are exclusively expressed in V0v interneurons, and, crucially, these Evx transcription factors are required for the glutamatergic neurotransmitter phenotypes of these cells [14, 17]. However, our previous analyses of zebrafish *evx1;evx2* double mutants did not detect changes in other V0v interneuron functional characteristics, such as axon trajectory, and, unlike in previous mouse studies, V0v interneurons in *evx1;evx2* double mutant zebrafish did not trans-fate into V1 interneurons [14, 17]. These data suggest that Evx1/2 are specifically required for the glutamatergic phenotype of V0v spinal interneurons in both zebrafish and mouse, but that these transcription factors may have additional functions in mammalian V0v spinal interneurons. Alternatively, as we only analyzed early developmental stages in our zebrafish studies, it is also possible that the zebrafish *evx1;evx2* mutant phenotype is more similar to that of mouse at later stages. To explore this possibility, in this study, we provide the first temporal analysis of V0v mutant phenotypes in any species.

In previous studies, we have started to define the GRNs downstream of Evx1/2 in V0v neurotransmitter fate specification. We have shown that Lmx1ba and Lmx1bb (Lmx1ba/b) act downstream of Evx1/2 in the specification and/or maintenance of V0v glutamatergic fates, and Evx1/2, but not Lmx1ba/b, are also required to repress inhibitory phenotypes in V0v interneurons [13, 14]. These data suggest that there may be two different important GRNs downstream of *evx1/2* in V0v spinal interneurons, one that specifies excitatory / glutamatergic fates and one that represses inhibitory / glycinergic fates.

To identify additional members of these essential GRNs, we FAC-sorted zebrafish V0v spinal interneurons and expression-profiled them using microarrays that contain probe-sets for all the genes in the zebrafish genome which encode proteins containing identified DNA-binding domains. In addition to *lmx1ba* and *lmx1bb*, we identified several other genes that are specifically enriched in V0v interneurons, compared to all post-mitotic spinal neurons and trunk cells. In this study, we examine the expression of these genes and their ohnologs during spinal cord development using in situ hybridization, and confirm that *skor1a, skor1b, skor2, ebf3a, uncx, nefma, nefmb, neff1* (formerly called *zgc:65851*) and *inab* are all expressed in appropriate spatio-temporal patterns to be part of GRNs that specify V0v interneuron neurotransmitter phenotypes. We then test whether the spinal cord expression of any of these genes is regulated by Evx1/2, by examining their expression in *evx1;evx2* double mutants by in situ hybridization and single-cell RNA-sequencing (scRNA-seq). We use these two complementary methods to examine two different stages of development. The scRNA-seq experiment also enables us to confirm that the gene expression changes we observe are specifically in V0v interneurons, and, importantly, to investigate for the first time whether there are distinct subsets of wild-type (WT) zebrafish V0v cells with different gene expression profiles. To develop future therapies to replace or repair damaged locomotor circuits, it is crucial that we understand whether distinct types of V0v interneurons exist, as well as how these cells are specified. The data from our experiments demonstrate that *skor1a, skor1b, skor2, ebf3a* and *neff1* all require Evx1/2 function for their expression in V0v interneurons, suggesting that these genes are part of GRNs downstream of Evx1/2 in these cells. In contrast, our results for *inab*, *nefma*, *nefmb* and *uncx* differ between our in situ hybridization and scRNA-seq experiments, suggesting that these genes may be differentially regulated by Evx1/2 at distinct stages of development. Taken together, these results identify several new candidates that may be part of GRNs that specify and/or maintain V0v functional properties.

Interestingly, our scRNA-seq data suggest the existence of two molecularly distinct clusters of WT V0v interneurons at 48 h. Similarly, there are two distinct clusters of what we presume are *evx1/2* single mutant cells, that are each most similar to a different WT cluster. Several of the transcription factors that we analyzed using in situ hybridization are more prominently expressed in one of the WT and single mutant cluster pairs than in the other one. Our scRNA-seq analyses also identified multiple additional transcription factors that are either downregulated or upregulated in *evx1/2* single mutant V0v cells, and are, therefore, strong candidates for being part of GRNs that specify and/or maintain V0v interneuron neurotransmitter phenotypes. Many of these are also more closely associated with one pair of WT and single mutant clusters than the other. Taken together, these data suggest that the two distinct subsets of V0v interneurons are specified by different GRNs. Our scRNA-seq data also suggest the intriguing possibility that by 48 h, subsets of *evx1;evx2* double mutant cells have transfated into either distinct types of inhibitory spinal interneurons, including a small group of V1 interneurons, or motoneurons. This is more reminiscent of the results in mouse *Evx1* mutants than our earlier data, suggesting that there is a higher level of conservation of Evx1/2 function between mammals and teleosts at later stages of development. Finally, and intriguingly, we show that Evx1/2 repress expression of *hmx2* and *hmx3a* in zebrafish V0v interneurons and that Hmx2/3a, in turn, repress expression of *skor1a* and *nefma* in dI2 interneurons. This suggests that Evx1/2 might regulate *skor1a* and *nefma* expression in V0v interneurons by repressing Hmx2/3a expression. Taken together, our data move us much closer towards the crucial goal of identifying the complete GRNs that specify the crucial neurotransmitter phenotypes of V0v spinal interneurons.

## Methods

### Ethics statement

All zebrafish experiments in this research were carried out in accordance with the recommendations and approval of Syracuse University Institutional Animal Care and Use (IACUC) committee.

### Fish lines

Zebrafish (*Danio rerio*) were maintained on a 14-h light / 10-h dark cycle at 28.5^◦^C. Embryos were obtained from natural paired and/or grouped spawnings of WT (AB, TL or AB/TL hybrid) fish, heterozygous *evx1*^*i232/*+^*;evx2*^*sa140/*+^ mutants [14], *Tg(pax2a:GFP)* transgenic fish [27], *Tg*(*evx1:EGFP*)^*SU1*^ and *Tg*(*evx1:EGFP*)^*SU2*^ (also known as *Tg(evx1-Mmu.Fos:GAL4-VP16,UAS:EGFP)*^*SU2*^*)* transgenic fish [14], heterozygous *hmx2;hmx3a*^*SU44;SU44*^ deletion mutants [12], or *Tg (hmx CNEIII:cfos:GAL4-VP16,UAS:EGFP)*^*SU41*^ transgenic fish (this publication).

### Morpholino injections

For double knockdown (DKD) translation-blocking experiments, 3.5 nl of a mixture containing 2 ng/nl each of a translation-blocking *hmx2* morpholino (5’ TTCCGCTGTCCTCCGAATTATTCAT) and a translation-blocking *hmx3a* morpholino (5’ ACGTATCCTGTGTTGTTTCGGGCAT), plus 5 ng/nl of a control zebrafish *p53* morpholino (5’ GCGCCATTGCTTTGCAAGAATTG) was injected into the single-cell of a one-cell stage *Tg(hmx CNEIII:cfos:GAL4-VP16,UAS:EGFP)*^*SU41*^ embryo (all morpholinos obtained from Gene Tools). DKD embryos exhibit delayed development from somitogenesis stages onwards when compared to uninjected controls. To circumvent this, they were incubated at 32°C from 9 h post fertilization (h) onwards, whereas control embryos remained at 28.5°C. This ensured that control and injected embryos reached the desired developmental stage of 27 h at approximately the same time. The lateral line primordium does not migrate in DKD animals, so this could not be used to stage injected embryos. Instead, these embryos were visually inspected and processed for fluorescence-activated cell sorting (FACS) when they displayed the same head-trunk angle, head size and eye size as prim-staged, uninjected control embryos [28]. Morpholino injections always produce a spectrum of phenotypes, since it is hard to ensure that every cell receives the same dose. Therefore, prior to processing for FACS at 27 h, we removed any embryos with severely abnormal morphology (stunted length and/or severely developmentally delayed, likely caused by receiving too much morpholino). DKD morphant embryos display a slight curled-tail-down morphology. Embryos that lacked this morphology (and may therefore not have received any or sufficient morpholino) were also removed before processing for FACS.

### Construction of *Tg(hmx:CNEIII:cfos:GAL4-VP16,UAS:EGFP)*^*SU41*^ Line

Potential *hmx* enhancer regions were identified by multispecies comparisons using Shuffle-LAGAN [29] and visualized using VISTA [30]. Zebrafish (*Danio rerio*) *hmx2* and *hmx3a* (ENSDARG00000070954 and ENSDARG00000070955 respectively, the two genes are adjacent on chromosome 17, Zv9) and orthologous sequences from human (ENSG00000188620, NCBI36 Ensembl release 54), mouse (ENSMUSG00000040148, NCBIM37 Ensembl release 54) and chicken (ENSGALG00000023415, Galgal4, Ensembl release 80) were obtained from Ensembl (http://www.ensembl.org). The *Xenopus tropicalis hmx3* (XB-GENE-483776) gene sequence was obtained from https://www.xenbase.org/entry/. *Danio rerio hmx2/hmx3a* sequence was used as baseline and annotated using exon/intron information from Ensembl. The alignment was performed using a 100 bp window and a cutoff of 70% identity. A comparison of approximately 62.5 Kb of *Danio rerio* genomic sequence extending 30 Kb upstream and 21 Kb downstream of *hmx2/hmx3a* identified three Conserved Non-coding Elements (CNEs) located either 5’ to *hmx3a* (CNE I and CNE II) or intergenic between *hmx3a* and *hmx2* (CNE III). CNE I is located 6193 bp upstream of the start codon of *hmx3a*. CNE II is located 2886 bp upstream of the start codon of *hmx3a*. CNE III is located 3199 bp downstream of the stop codon of *hmx3a* and 3297 bp upstream of the start codon of *hmx2*. Using genomic DNA, we PCR-amplified amplicons of 690 bp, 480 bp and 919 bp for CNE I, CNE II and CNE III respectively, using the following primers:

FW Hmx3 CNEI: CTCTCTGGGCGAAACAGCAC,

RV Hmx3 CNEI: ACACAGGTGATGCCTTCCAC,

FW Hmx3 CNEII: ATACGTGGGCAATTACAGCG,

RV Hmx3 CNEII: ATGGCAGGCCTACATCATCC,

FW Hmx3 CNEIII: AATAGACGGCGAGAACGTGA,

RV Hmx3 CNEIII: CCGGCTGAACAGGCTTTTTG.

PCR conditions were: 98°C for 30 s, followed by 30 cycles of 98°C for 10 s, 62°C for 30 s, 72°C for 30 s, and a final 10 min extension step at 72°C.

Separate reporter constructs were generated for each of the three *hmx* CNEs. First, the 690 bp (CNE I), 480 bp (CNE II), and 919 bp (CNE III) amplicons were cloned into the pDONR™ P4-P1R vector from Invitrogen using Gateway technology [31, 32]. Constructs were assembled using each of the *CNE I, CNE II* and *CNE III hmx* 5′ pDONR constructs with the *cfos minimal promoter:Gal4VP16,UAS:EGFP* middle entry construct [14, 33] and the pCSDest2 vector [34] to generate *Tg(Tol2:hmx CNEI:cfos minimal promoter:Gal4VP16,UAS:EGFP:pA:Tol2), Tg(Tol2:hmx CNEII:cfos minimal promoter:Gal4VP16,UAS:EGFP:pA:Tol2)*, and *Tg(Tol2:hmx CNEIII:cfos minimal promoter:Gal4VP16,UAS:EGFP:pA:Tol2)*.

Plasmid DNA and *transposase* mRNA for microinjection was prepared as in [35, 36]. Approximately 10 nl of a combination of plasmid DNA [60–80 ng/μl] and *transposase* mRNA [30 ng/μl] was injected into both blastomeres of 1–2-cell stage zebrafish embryos. F0 embryos injected with either *Tg(Tol2:hmx CNEI:cfos minimal promoter:Gal4VP16,UAS:EGFP:pA:Tol2)* or *Tg(Tol2:hmx CNEII:cfos minimal promoter:Gal4VP16,UAS:EGFP:pA:Tol2)* displayed only weak, ectopic EGFP expression in the heart, notochord and skin. We did not observe expression in the ear, lateral line primordium or spinal cord. Therefore, since none of these expression locations resembled endogenous *hmx2/3a* expression, we did not pursue these transgenic lines further. *Tg(Tol2:hmx CNEIII:cfos minimal promoter:Gal4VP16,UAS:EGFP:pA:Tol2)*-injected embryos showed EGFP expression in the spinal cord, similar to endogenous *hmx* expression and were raised to adulthood and out-crossed to identify founders to generate the stable *Tg(hmx CNEIII:cfos:GAL4-VP16,UAS:EGFP)*^*SU41*^ line which we used in the experiments in this paper. Note though that this line also does not recapitulate endogenous *hmx3a* expression in either the ear or lateral line primordium, suggesting that the enhancer region(s) driving expression in these tissues is not present in CNE III (data not shown).

### in situ hybridisation and immunohistochemistry

We fixed embryos in 4% paraformaldehyde/phosphate-buffered saline (PBS) and performed single in situ hybridizations and immunohistochemistry plus in situ hybridization double-labelling experiments as previously described [37, 38]. Sources of in situ hybridization probes are provided in Supp. Table 1. PCR-based in situ probes were created with cDNA from 27 h WT zebrafish embryos. We extracted total RNA by homogenizing 50–100 mg of embryos in 1 ml of TRIzol reagent (Ambion, 15596026). We confirmed RNA integrity (2:1 ratio of 28S:18S rRNA bands) and quality (A260/A280 ratio of ~ 2.0) using agarose gel electrophoresis and spectrophotometry respectively. We synthesized cDNA using Bio-Rad iScript Reverse Transcription Supermix kit (Bio-Rad, 1708891). We amplified each sequence using Phusion High-Fidelity DNA Polymerase (M0530L, NEB) and 5 µl cDNA in 50 µl total reaction volumes, using the primers and annealing temperatures shown in Supp. Table 1. To avoid cross-reactivity, whenever possible, riboprobes were designed against 3’UTR or coding sequence lacking all conserved protein domains in Pfam [39]. Primers were designed using Primer3 web version 4.1.0 at https://primer3.ut.ee [40, 41] and the following design parameters: optimum primer size: 22 bp (minimum: 20 bp, maximum: 25 bp), optimum annealing temperature: 58.0°C (minimum: 57.0°C, maximum: 60.0°C), and optimum GC content: 50% (minimum: 40%, maximum: 60%). The preferred product size range was 800–1100 bp. This was not always possible, if there was little or no novel coding and/or 3’ UTR sequence available (see Supp. Table 1). The PCR conditions were: 98.0°C for 30 s, 35 cycles of: 98.0°C for 10 s; annealing temperature in Supp. Table 1 for 30 s and 72.0°C for 30 s, followed by a final extension for 5 min at 72.0°C. The PCR product was assessed on a 1% TAE gel, before purifying through phenol:chloroform:isoamyl alcohol extraction and precipitation with 0.2 M NaCl and ice-cold ethanol. If non-specific banding was generated in addition to the desired PCR product, the specific product was purified from the agarose gel using the Monarch DNA Gel Extraction Kit (NEB, T0120S). Each reverse primer contains the T3 RNA Polymerase minimal promoter sequence (shown in bold and underlined in Supp. Table 1). in situ probe synthesis was performed using 1 µg purified PCR product, T3 RNA Polymerase (Roche, 11031171001) and DIG RNA Labeling Mix (Roche, 11277073910).

Embryos older than 24 h were usually incubated in 0.003% 1-phenyl-2-thiourea (PTU) to prevent pigment formation. For some experiments we added 5% of Dextran Sulfate to the hybridization buffer (* in Table 1). Dextran sulfate can increase specific staining in in situ hybridization experiments as it facilitates molecular crowding [42, 43].

**Table 1:** Statistical comparisons of numbers of cells expressing particular genes in mutant experiments

Fig.	Comparison	Gene	Difference between two means	*P*-value
4C	30 h WT (63.3 ± 2.3) vs *evx1;evx2* (39.8 ± 2.7)	skor1a*	24↓	** < 0.001**^**+**^
4F	30 h WT (61.3 ± 2.0) vs *evx1;evx2* (12.7 ± 1.2)	skor1b*	49↓	** < 0.001**^**+**^
4I	30 h WT (46.7 ± 1.5) vs *evx1;evx2* (22.6 ± 0.7) — all labelled cells	*skor2*	24↓	** < 0.001**^**+**^
N.S	30 h WT (24.3 ± 1.3) vs *evx1;evx2* (0.1 ± 0.1) — ventral labelled cells only	*skor2*	24↓	**0.002**^**^**^
4L	30 h WT (49.2 ± 2.9) vs *evx1;evx2* (24.0 ± 3.7)	*ebf3a*	25↓	**0.019**^**^**^
4O	30 h WT (111.8 ± 3.4) vs *evx1;evx2* (107.3 ± 2.6)	*uncx*	5↓	0.333^+^
5C	30 h WT (171.0 ± 5.9) vs *evx1;evx2* (151.2 ± 5.9)	nefma*	20↓	**0.045**^**^**^
5F	30 h WT (107.5 ± 19.1) vs *evx1;evx2* (119.0 ± 8.0)	*nefmb*	12↑	0.565^+^
5I	30 h WT (81.6 ± 6.9) vs *evx1;evx2* (45.0 ± 5.4)	neff1*	37↓	**0.001**^**+**^
5L	30 h WT (195.4 ± 9.7) vs *evx1;evx2* (197.6 ± 6.5)	*inab*	2↑	0.855^+^
Supp. 2D	30 h WT (29.0 ± 2.1) vs *evx1;evx2* (23.4 ± 3.4)	*nefla*	6↓	0.202^+^
Supp. 2G	30 h WT (81.8 ± 2.5) vs *evx1;evx2* (81.8 ± 3.4)	*neflb*	0	0.991^+^
11C	30 h WT (111.2 ± 12.4) vs *evx1;evx2* (157.2 ± 8.1)	*hmx3a*	46↑	**0.015**^**+**^
12D	27 h WT (46.0 ± 2.8) vs *hmx2;hmx3a* (47.4 ± 2.4)	evx1*	1↑	0.718^+^
12G	27 h WT (39.6 ± 3.0) vs *hmx2;hmx3a* (38.0 ± 2.9)	evx2*	2↓	0.713^+^
12J	27 h WT (61.8 ± 2.7) vs *hmx2;hmx3a* (107.4 ± 2.2)	*skor1a*	46↑	**0.008**^^^
12M	27 h WT (33.4 ± 1.6) vs *hmx2;hmx3a* (30.8 ± 1.5)	*skor1b*	3↓	0.264^+^
12P	27 h WT (48.4 ± 1.7) vs *hmx2;hmx3a* (43.8 ± 2.8)	*skor2*	5↓	0.197^+^
12S	27 h WT (51.9 ± 0.8) vs *hmx2;hmx3a* (56.6 ± 2.0)	*ebf3a*	5↑	0.079^^^
12V	27 h WT (84.3 ± 4.8) vs *hmx2;hmx3a* (162.4 ± 6.2)	*nefma*	78↑	** < 0.001**^+^
12Y	27 h WT (44.2 ± 2.6) vs *hmx2;hmx3a* (48.0 ± 2.4)	*neff1*	4↑	0.376^+^

Statistical comparisons between WT and mutant embryos. *evx1;evx2* = *evx1*^*i232/i232*^*;evx2*^*sa140/sa140*^ double mutant embryos and *hmx2;hmx3a* = *hmx2;hmx3a*^*SU44/SU44*^ deletion mutants. First column indicates the figure panel that contains the values plot for the comparison. N.S. = data not shown. Second column states the age (h) and genotypes being compared. Numbers within parentheses indicate mean numbers of cells ± standard error of the mean (S.E.M.). In all cases, these values are the mean of at least 3 embryos and in all cases except the fourth data row of the table, cells were counted in all dorsal–ventral spinal cord rows. Column three lists the gene that the cell counts, and statistical comparison refer to. *Asterisks indicate experiments performed with Dextran Sulfate (see Methods). The fourth column indicates the difference between the two mean values for the embryos being compared. All values are rounded to the nearest whole number. ↑ = increase, ↓ = decrease. Last column shows the *P-*value for the comparison, rounded to three decimal places. Statistically significant (*P* < 0.05) values are indicated in bold. Statistical test used is indicated by superscript symbol: Wilcoxon-Mann–Whitney test (^^^), or type 2 Student’s t-test (^+^). For a discussion of why particular tests were used, see Methods

In cases where we did not detect expression of a particular gene in the spinal cord, we checked for low levels of expression by exposing embryos to prolonged staining. In some cases, this produced higher background (diffuse, non-specific staining), especially in the hindbrain, where ventricles can sometimes trap anti-sense riboprobes.

For in situ hybridization and immunohistochemistry double-labelling experiments, after detection of the in situ hybridization reaction using either Tyramide SuperBoost Kit B40915 (with HRP, Goat anti-mouse IgG and Alexa Fluor 594 Tyramide, ThermoFisher Scientific) or NBT/BCIP (Roche, 11681451001), embryos were washed 8 × 15 min in PBST (PBS with 0.1% Tween-20) and incubated in Image-iT FX Signal Enhancer (ThermoFisher Scientific, I36933) for 30 min at room temperature. Immunohistochemistry was performed using chicken polyclonal anti-GFP primary antibody (Ab13970, Abcam, 1:500) and a Goat anti-chicken IgY (H + L), Alexa Fluor 488 secondary antibody (A-11039, ThermoFisher Scientific, 1:1000).

Sometimes we were unable to perform double fluorescent staining experiments due to very weak labelling with our RNA probes. In these cases, we combined single fluorescent labelling with NBT/BCIP chromogenic staining. However, whereas stronger in situ signals from weak RNA probes can be obtained using NBT/BCIP, visualization of co-expressing cells becomes more difficult with this method. To preserve the integrity of both the NBT/BCIP chromogenic in situ signal and the weaker IHC signal, embryos were stored and mounted in VECTASHIELD Antifade Mounting Medium (Vector Laboratories, H-1000–10).

### Fluorescent-activated cell sorting (FACS)

The microarray expression profiling experiments are described in detail in [13, 44]. *P*-values were corrected for multiple testing [45–47]. These data have been deposited in the NCBI Gene Expression Omnibus with accession number GSE145916.

For single-cell RNA-seq (scRNA-seq) experiments, embryos were obtained from crossing heterozygous *evx1*^*i232/*+^*;evx2*^*sa140/*+^ fish that were homozygous for *Tg(evx1:EGFP)*^*SU2*^. Embryos were screened for fluorescence from 30 h onwards using a fluorescent dissecting microscope. Only EGFP-positive embryos were used for dissections and FACS at 48 h. These experiments were performed at 48 h because EGFP is expressed in significantly more V0v interneurons at this time point than at earlier developmental stages [14]. Only 1/16 embryos will be double mutants from an incross of *evx1;evx2* heterozygous parents, and we are limited in the number of embryos that we can dissect for each experiment as we need to limit the time that dissected trunks wait on ice before being dissociated and FAC-sorted (see below).

For bulk RNA-seq experiments, uninjected control embryos and *hmx2;hmx3a* DKD morphant embryos in the *Tg(hmx CNEIII:cfos:GAL4-VP16,UAS:EGFP)*^*SU41*^ background (generated as described above) were screened for fluorescence from 24 h onwards. Only EGFP-positive control and *hmx2;hmx3a* DKD morphant animals were used for dissociation and FACS at 27 h. For qRT-PCR experiments, fluorescent embryos were generated from incrosses of homozygous *Tg(hmx CNEIII:cfos:GAL4-VP16,UAS:EGFP)*^*SU41*^ fish and used for dissociation and FACS at 27 h. Stage-matched embryos from WT incrosses were used as negative controls for FACS set-up.

For all these experiments, embryos were deyolked, and trunks were dissected and dissociated as described in [44], with the following modifications: Trunk tissue was dissected anteriorly at the boundary between the hindbrain and spinal cord, and posteriorly, immediately above the end of the yolk extension. Embryos were processed in batches of 50 and stored on ice for a maximum of two hours prior to dissociation, to preserve cell and mRNA viability. To ensure complete dissociation of trunk tissue with the Papain Dissociation System (Worthington Biochemical Corporation, LK003150), trunks from 27 and 48 h samples were incubated in 1 ml Papain/DNase mix with gentle rocking at 28.5°C for 30 min and 40 min respectively. The digested tissue was then allowed to settle for 10 s before the Papain/DNase mix was carefully decanted until approximately 500 µl remained. Immediately after homogenising the digested tissue mixture with a sterile p200 tip, we passed each sample through a 40 µm Flowmi cell strainer (Merck, BAH136800040) into a sterile microcentrifuge tube. After Papain inactivation, samples were resuspended in 1 ml Leibovitz’s L-15 medium (ThermoFisher Scientific, 21083027) + 0.5% FBS (Gibco, ThermoFisher Scientific, 16000036) and stored on ice. Immediately before FACS, DAPI (Merck, D9542) and Draq5 (BioLegend, 424101) were added at a final concentration of 5 µg/ml and 5 µM respectively. To further maintain maximum cell viability and preserve endogenous mRNA expression, which are the most significant technical barriers to transcriptional profiling [48], FACS was performed no later than four hours after beginning embryo deyolking and dissection.

FACS was performed using a Becton Dickinson FACS Aria III Cell Sorter at the SUNY Upstate Medical University Research Flow Core using the parameters described previously [44] with the following modifications. Ice-cold samples were filtered through 35 µm mesh strainers into 5 ml round-bottomed polystyrene tubes (Corning Falcon, 352235). All FAC-sorting and collection steps were performed at + 4°C, using a 100 µm nozzle and 20 psi sort pressure. Successive doublet exclusion gates (forward scatter height x forward scatter width, followed by side scatter height x side scatter width) were used to finesse capture of real single cells. Accurate live/dead filtering was performed by selecting for DAPI-negative (sick cells are DAPI-permeant and excluded) and Draq-5-positive (only healthy nuclei are Draq-5 permeant) cells, as described by Lush and colleagues [49].

For our 27 h bulk RNA-seq and qRT-PCR experiments, cells were sorted directly into sterile 1.5 ml microcentrifuge tubes containing 100 µl of Buffer RLT (Qiagen RNeasy Micro Kit, 74004) plus 143 mM β-mercaptoethanol. Sorted cells were stored at -80°C prior to RNA extraction. On average, 1.38% and 1.12% of the cells that we sorted, from uninjected control and *hmx2;hmx3a* double morphant embryos respectively, were EGFP-positive. After FAC-sorting, 100% of all sorted cells were EGFP-positive.

For our 48 h *evx1*^*i232/*+^*;evx2*^*sa140/+*^*;Tg(evx1:EGFP)*^*SU2*^ scRNA-seq experiments, EGFP-positive cells were sorted and fixed using a methanol fixation protocol modified from the 10 × Genomics Sample Preparation Demonstrated Protocol “Methanol Fixation of Cells for Single Cell RNA Sequencing” (https://www.10xgenomics.com). EGFP-positive cells were sorted directly into 5 ml round-bottomed tubes containing 3.5 mls of freshly made, pre-chilled, 90% methanol (for HPLC, > 99%, Merck, 34860)/10% Dulbecco’s Phosphate-Buffered Saline (DPBS, No calcium, No magnesium, Merck, D8537) fixative. A tube of EGFP-negative cells was also collected to assess fixation efficiency. On average, 3.875% of the cells that we sorted were EGFP-positive. After FAC-sorting, on average, 94.715% of the collected cells were EGFP-positive. Sorted cells were incubated on ice for 1 h before assessing fixation efficiency of the EGFP-negative control tube using Trypan Blue (ThermoFisher Scientific, 15250061) and a hemocytometer. Samples with intact, fully fixed cells, containing little or no cell debris were stored at + 4°C for up to six days prior to rehydrating and performing single-cell capture with the 10 × Genomics Chromium system (see below).

### Single-cell RNA-seq

To rehydrate our fixed EGFP-positive 48 h *evx1*^*i232*^*;evx2*^*sa140*^*;Tg(evx1:EGFP)*^*SU2*^ cells (stored at + 4°C for up to six days post-FACS – see above), we first centrifuged each sample at 300 rcf for 10 min at + 4°C using a swing-bucket centrifuge. We recommend against using a fixed rotor centrifuge as this can severely reduce recovery yields. Note that we never observed cell pellets during this step. Therefore, we marked the outer side of each 5 ml round-bottomed sample tube prior to centrifuging and avoided decanting from this side of the tube to prevent disruption and loss of the cell pellet. Next, we carefully removed most of the supernatant with a sterile p1000 tip, until approximately 100 µl remained in the tube. Samples were always kept on ice. Each cell pellet was then gently resuspended by adding 2 ml of freshly made, pre-chilled Rehydration Buffer (1 × Dulbecco’s Phosphate-Buffered Saline, no calcium, no magnesium (Merck, D8537), 1.0% UltraPure BSA (ThermoFisher Scientific, AM2616), 0.5 u/µl Roche Protector RNase Inhibitor (Merck, 3335402001)) and gently pipetting 10 times. It is important to avoid making foam. We repeated the centrifugation and resuspension in Rehydration Buffer steps as previously. After the second Rehydration step, we again centrifuged at 300 rcf for 10 min at + 4°C before carefully removing all but 30–40 µl of supernatant. Using a sterile p200 tip, we carefully resuspended the cell pellet and immediately measured the cell concentration in triplicate using a Bio-Rad TC20 automated cell counter (Bio-Rad, 1450102). We also checked a small aliquot using a compound microscope to ensure we had single cell suspensions. As described by 10 × Genomics in their Sample Preparation Demonstrated Protocol “Methanol Fixation of Cells for Single Cell RNA Sequencing” (https://www.10xgenomics.com), we too recovered approximately 50% of the sorted cells after rehydration. Therefore, we rehydrated cells from four separate FACS experiments prior to performing single-cell capture.

We isolated single cells using a 10 × Genomics Chromium system, aiming for capture of 10,000 cells per well (Chromium Next GEM Chip G Single Cell Kit, 1000127). We loaded 4 wells in total. This, and all subsequent library preparation steps were performed at the SUNY Upstate Medical University Molecular Analysis Core. We prepared libraries using a 10 × Genomics Chromium Next GEM Single Cell 3’ GEM, Library and Gel Bead Kit (v3.1, 10 × Genomics, 1000128) and sequenced them on an Illumina NextSeq500 to a depth of at least 50,000 reads per cell (Illumina NextSeq 500/500 High Output Kit, v2.5, 150 cycles, 20024907). We then performed demultiplexing and counts analysis as per the manufacturer’s instructions using Cell Ranger v4.0.0 software (https://www.10xgenomics.com) and the Lawson Lab zebrafish transcriptome annotation model V4.3.2 [50]. We analyzed the data using Partek Flow Genomic Analysis Software [51]. Multiplets were removed by filtering out cells with > 12,000 counts and > 2,500 detected genes. Sick and/or “leaky” cells were removed by filtering out cells with < 500 detected genes and > 6% mitochondrial transcripts. We normalized the data using a counts per million (CPM) algorithm and applied a logarithmic transformation to improve data visualization. The outcome of normalization was assessed by principal components analysis (PCA), graph-based clustering and Uniform Manifold Approximation and Projection (UMAP) plotting, using the NN-Descent method of nearest neighbor type calculation and Euclidean distance metrics. We manually inspected 2D UMAP plots to assess clustering quality based on expression of known V0v spinal interneuron markers. We excluded immature spinal cells that had begun to express the transgene but otherwise lacked expression of V0v post-mitotic genes to focus our analysis only on post-mitotic, differentiated V0v cells. We then fine-tuned the clustering by manually deducing and extrapolating cell fate assignments by comparing expression profiles of 48 h single-cell clusters with the molecular phenotypes of V0v spinal interneurons in 24 h and 30 h WT, *evx1*^*i232/i232*^ and *evx2*^*sa140/sa140*^ single mutant and *evx1*^*i232/i232*^*;evx2*^*sa140/sa140*^ double mutant embryos, as described by Juárez-Morales and colleagues [14] and data in this study (see Results). To perform gene-specific analyses (GSA) of differential expression, we used the statistically robust Hurdle Model with default parameters in Partek Flow [51]. Under these conditions, the Hurdle Model in Partek Flow is equivalent to the widely used Model-based Analysis of Single-cell Transcriptomics (MAST) framework, which also incorporates Hurdle modelling [52]. Hurdle models deal efficiently with the sources of nuisance variation commonly associated with single-cell datasets, such as sparsely detected cells (which influence the cellular detection rate, an indicator of technical and/or biological variability between samples) and bimodal gene expression values (where many genes have zero expression values in the matrix, which can bias the interpretation of how much genes above the detection threshold are really expressed) [53, 54]. When we examined our single-cell data, we observed that there were several small subsets of cells within Mutant Group 3 with distinct transcriptional profiles. Each of these subsets had so few cells, that we were concerned that this would underpower the Hurdle model by compromising the effectiveness of the variance modelling. To overcome this limitation, and further aid determination of differential expression, we also performed analysis of variance (ANOVA) using default parameters in Partek Flow [51]. Unlike the Hurdle model, ANOVA models the expression of each gene independently of all the others, and Nault and colleagues have shown that it is the best method for calculating differential expression in scRNA-seq data when cell numbers are small [55]. Therefore, we provide the data from both Hurdle modelling and ANOVA for analytical rigor.

We analyzed each of our four libraries separately. We omitted two of our libraries from further downstream analysis due to significant presence of notochord cells (which variably and ectopically express our *Tg(evx1:EGFP)*^*SU2*^ construct). This ectopic expression was much less abundant in our remaining two libraries and so these were used for the analysis shown in this paper. The sequencing depth for these two libraries approaches saturation (81.9% for one library and 89.3% for the other library), providing a high probability of detecting transcripts expressed at low levels. We captured 61,748 and 95,333 mean reads per cell, plus 1,718 and 1,460 median genes per cell for each of these two libraries respectively. We combined the data from these two libraries using the Counts Aggregation pipeline in Cell Ranger v4.0.0 and reanalyzed the data as described above. For the combined data, we identified 2860 cells that passed quality controls and V0v cell fate assignment (see Results).

### Bulk RNA-Seq

EGFP-positive cells were FAC-sorted from 27 h uninjected control and *hmx2;hmx3a* DKD *Tg(hmx CNEIII:cfos:GAL4-VP16,UAS:EGFP)*^*SU41*^ embryos as described above. RNA extractions were performed using a method based on that of [56] with the following modifications. Prior to performing RNA extractions, all work surfaces and pipettors were treated with RNaseZAP (ThermoFisher Scientific, AM9780). Throughout the process, samples were stored on ice unless otherwise stated. Frozen FAC-sorted cell lysates were removed from storage at -80°C and thawed in a 37°C water bath, before transferring to sterile microcentrifuge tubes. If necessary, sample volumes were increased to 250 µl with UltraPure DNase/RNase-Free distilled water (ThermoFisher Scientific, 10977035). 750 µl TRIzol LS Reagent (ThermoFisher Scientific, 10296028) was added to each 250 µl sample, before homogenising by gently pipetting up and down ten times with a sterile p1000 pipette tip. Samples were immediately transferred to Phasemaker tubes (which had been pre-centrifuged as per the manufacturer’s instructions (ThermoFisher Scientific, A33248)), before incubating for 5 min at room temperature. 200 µl chloroform was added to each sample. The tubes were then shaken vigorously for 15 s and incubated for a further 5 min at room temperature. The samples were then centrifuged for 5 min at 16,000 × g at 4°C, before transferring the RNA-containing upper aqueous phase to a sterile centrifuge tube and adding one volume of 70% RNase-free ethanol. Samples were inverted to mix thoroughly, and the supernatant immediately loaded to an RNeasy MinElute column (from the RNeasy Micro Kit, Qiagen, 74004), before centrifuging for 15 s at 10,000 rpm. Wash steps with RW1 buffer, RPE buffer and 80% RNase-free ethanol were performed as per the RNeasy Micro Kit instructions. Samples were eluted in 14 µl RNase-free water. RNA integrity was assessed with the Agilent RNA 6000 Pico chip (Agilent, 5067–1513) on an Agilent 2100 Bioanalyzer. Only samples with RNA integrity (RIN) values > 9 were used for library preparation. RNA concentrations were measured with the Qubit RNA High-Sensitivity Assay Kit (ThermoFisher Scientific, Q32852) and a Qubit 3.0 fluorometer (ThermoFisher Scientific, Q33216).

cDNA synthesis and the subsequent library preparation steps were performed at the SUNY Upstate Medical University Molecular Analysis Core. cDNA was synthesised using the SMART-Seq v4 Ultra Low Input RNA Kit for Sequencing (Takara, 634888), and used to make sequencing libraries with the Nextera XT DNA Library Preparation Kit (Illumina, FC-131–1024). cDNA and library quality were measured with the Agilent High Sensitivity DNA Kit (Agilent, 5067–4626) on an Agilent 2100 Bioanalyzer. We prepared individual libraries for five biological replicates of uninjected control;*Tg(hmx CNEIII:cfos:GAL4-VP16,UAS:EGFP)*^*SU41*^ EGFP-positive FAC-sorted cells and for five biological replicates of *hmx2;hmx3a* DKD;*Tg(hmx CNEIII:cfos:GAL4-VP16,UAS:EGFP)*^*SU41*^ EGFP-positive FAC-sorted cells. Libraries were sequenced on an Illumina NextSeq500 to a depth of 20 million reads per sample (Illumina NextSeq 500/500 High Output Kit, v2.5, 75 cycles, Catalog # 20024906).

The data was analyzed using Partek Flow Genomic Analysis Software [51]. We first performed pre-alignment quality control assessment and recovered a minimum average read length of 74 bases and a minimum average read quality (Phred score) of 33.99, suggesting we recovered high quality, accurate sequencing data. Next, we trimmed the adapter sequence “CTGTCTCTTATACACATCT” from the 3’ end using default parameters, before trimming bases from the 5’ end. We selected an end minimum quality value (Phred) score of 32, and a minimum read length of 65 bases. Consequently, we trimmed an average of 1.14–1.15 bases. We aligned reads using default parameters and the STAR-2.6.1d algorithm, together with the Lawson Lab zebrafish transcriptome annotation model V4.3.2 [50]. We aligned a minimum of 95.54% of all reads, with a minimum of 92.31% of reads aligning uniquely to the genome. Of these, a minimum 81.28% of reads aligned fully within an exon, a maximum 6.59% of reads aligned partly within an exon, a maximum 4.34% of reads aligned fully within an intron, and a maximum of 7.79% of reads were fully intergenic. We normalized the log expression ratios using a Trimmed Means of M-values (TMM) weighted algorithm [57]. We performed differential expression analysis using the Gene-Specific Analysis (GSA) algorithm in Partek Flow. We used GSA because it makes no assumptions in advance about the data distribution nor the model choice necessary to deal with any nuisance factors present in the data. Rather, GSA describes transcript expression by calculating the data distribution and appropriate statistical model for each transcript in turn. As such, GSA can yield more accurate and reproducible expression data across the entire dataset, rather than just for the most pronounced expression outliers, as may be obtained with more common differential expression tools such as DEseq2 and limma (see Partek Flow Gene-Specific Analysis white paper: https://documentation.partek.com/display/FLOWDOC/Gene-specific+Analysis). The outcome of GSA was assessed by hierarchical clustering (heatmap) plotting, clustering by features, using average linkage and Euclidean cluster distance and point distance metrics respectively.

### qRT-PCR Analyses

EGFP-positive and EGFP-negative cells were FAC-sorted from 27 h *Tg(hmx CNEIII:cfos:GAL4-VP16,UAS:EGFP)*^*SU41*^ embryos as described above. Total RNA was extracted as per the protocol used for our bulk RNA-seq experiments, with the exception that the final eluted total RNA was divided in to 2–3 µl RNA aliquots in sterile PCR tubes and stored at -80°C. cDNA was synthesized using the Bio-Rad iScript Reverse Transcription Supermix kit (Bio-Rad, 170–8891) and 3 µl of purified total RNA. We also included controls lacking Reverse-Transcriptase to assay for the presence of genomic DNA contamination. qRT-PCR was performed in triplicate for each sample using iTaq Universal SYBR Green Supermix (1725121, Bio-Rad) and a BioRad CFX96 real-time PCR machine. The following qPCR primers were used:

*hmx2*-qPCR-FW: CCCATTTCAAGTTTCACGATCCAGTC,

*hmx2*-qPCR-RV: TGCTCCTCTTTGTAATCCGGTAG,

*hmx3a*-qPCR-FW: TTGATGGCAGCTTCTCCCTTTC,

*hmx3a*-qPCR-RV: ACTCTTCTTCCAGTCGTCTATGC,

*slc17a6b*-qPCR-FW: GGTGTGTCCTCTTATTGTCGGAG,

*slc17a6b*-qPCR-RV: GCCAGCTCGTCTTCATCAATG,

*slc32a1*-qPCR-FW: AACCCGGACAAGCCCAGAATC,

*slc32a1-*qPCR-RV: GTCTCTCACTCGCACCAACTG,

*actb2*-qPCR-FW: GCAGAAGGAGATCACATCCCTGGC,

*actb2*-qPCR-RV: CATTGCCGTCACCTTCACCGTTC,

The *slc17a6b* and *slc32a1* primers were generated in this study. We generated the *hmx2* and *hmx3a* primers in a previous study [12]. The *actb2* primers were generated by Hu and colleagues [58]. To generate amplification data the program used was: 95.0°C for 30 s, 40 cycles of: 95.0°C for 5 s, 63.3°C (*hmx2*)/64.5°C (*hmx3a*)/65.0°C (*slc17a6b*)/55.7°C (*slc32a1*)/60.0°C (*actb2*) for 30 s, with imaging after each cycle. To assay amplification specificity and exclude false positives from primer dimers we then generated melt data using: 65.0°C for 30 s, 40 cycles of: 65.0°C-95.0°C, + 0.5°C/second increment, with each increment held for 5 s prior to imaging, 95.0°C for 15 s.

### Imaging

Embryos from single NBT/BCIP in situ hybridization experiments were mounted in 70% glycerol:30% distilled water between coverslip sandwiches (24 mm × 60 mm coverslips; VWR, 48393-106), with 2–4 coverslips (22 mm × 22 mm; VWR, 16004-094) on either side of the sample to avoid sample compression. Differential Interference Contrast (DIC) pictures were taken using an AxioCam MRc5 camera mounted on a Zeiss Axio Imager M1 compound microscope. Embryos from fluorescent in situ hybridization + immunohistochemistry experiments were mounted in VECTASHIELD Antifade Mounting Medium (Vector Laboratories, H-1000–10) between coverslip sandwiches. Fluorescent images were taken on a Zeiss LSM 710 confocal microscope. Embryos from NBT/BCIP in situ hybridization + fluorescent immunohistochemistry experiments were also mounted in VECTASHIELD Antifade Mounting Medium (Vector Laboratories, H-1000–10) between coverslip sandwiches. NBT/BCIP and fluorescent images were captured using the T-PMT and 488 nm channels respectively on a Zeiss LSM 710 confocal microscope. Images were processed using Adobe Photoshop software (Adobe, Inc) and Image J software [59]. NBT/BCIP confocal images (captured from in situ hybridization + immunohistochemistry experiments) are grayscale and were subsequently pseudo-colored in Photoshop by converting the image mode from Grayscale to Duotone. A custom purple ink tone (*R* = 48, G = 5, B = 107) was then applied and the image mode switched once more to RGB. The coloring now reproduces that of endogenous NBT/BCIP staining. In some cases, different focal planes were merged to show labelled cells at different medial–lateral positions in the spinal cord. All images were processed for brightness-contrast and color balance using Adobe Photoshop software (Adobe, Inc.). Images of control and mutant embryos from the same experiment were processed identically. Figures were assembled using Adobe Photoshop (Adobe, Inc.).

### Cell counts and statistics

In all cases, cell counts are for both sides of a five-somite length of spinal cord adjacent to somites 6–10. Embryos were mounted laterally with the somite boundaries on each side of the embryo exactly aligned and the apex of the somite over the middle of the notochord. This ensures that the spinal cord is straight along its dorsal–ventral axis and that cells in the same dorsal–ventral position on opposite sides of the spinal cord will be directly above and below each other. Embryos from mutant crosses were counted blind to genotype. Labelled cells in embryos analyzed by Differential Interference Contrast (DIC) microscopy were counted while examining embryos on a Zeiss Axio Imager M1 compound microscope. We adjusted the focal plane as we examined the embryo to count cells at all medial–lateral positions (both sides of the spinal cord; also see [6, 13, 14, 37, 60]).

In some cases, cell count data were pooled from different experiments. Prior to pooling, all pairwise combinations of data sets were tested to determine if there were any statistically significant differences between them as described below. Data were only pooled if none of the pairwise comparisons were statistically significantly different from each other. In addition, as in situ hybridization staining can vary slightly between experiments, we only compared different mutant results when the counts from their corresponding WT sibling embryos were not statistically significantly different from each other.

To determine whether differences in values are statistically significant, data were first analyzed for normality using the Shapiro–Wilk test. Data sets with non-normal distributions were subsequently analyzed using the Wilcoxon-Mann–Whitney test (also called the Mann Whitney U test). For data sets with normal distributions, the F-test for equal variances was performed, prior to conducting a type 2 (for equal variances) student’s *t*-test. *P*-values generated by Wilcoxon-Mann–Whitney test and type 2 student’s *t*-tests are indicated by ^^^ and ^+^. Data are depicted as individual value plots and the *n*-values for each experimental group are also shown. For each plot, the wider red horizontal bar depicts the mean, and the red vertical bars depict the standard error of the mean (standard error of the mean (S.E.M.) values are listed in Table 1). Individual data value plots were generated using Prism version 9.4.0 (GraphPad Software, San Diego, California USA, www.graphpad.com). To assess whether mutant phenotypes occurred at Mendelian frequencies, we performed Chi-squared tests. Shapiro–Wilk and Wilcoxon-Mann–Whitney testing was performed in R version 3.5.1 [61]. The F-test, student’s *t*-test, and Chi-squared test were performed in Microsoft Excel version 16.62.

### Data and reagent availability

Plasmids and zebrafish strains are available upon request. Microarray data were previously deposited in the NCBI Gene Expression Omnibus with accession number GSE145916. Single-cell and bulk RNA-Seq data have been deposited in the NCBI Gene Expression Omnibus with accession numbers GSE240239 and GSE240238 respectively.

## Results

### *skor1a, skor1b, skor2, ebf3a, uncx, nefma, nefmb, neff1 *and* inab* are all expressed in the V0v spinal cord region

**Fig. 1:**
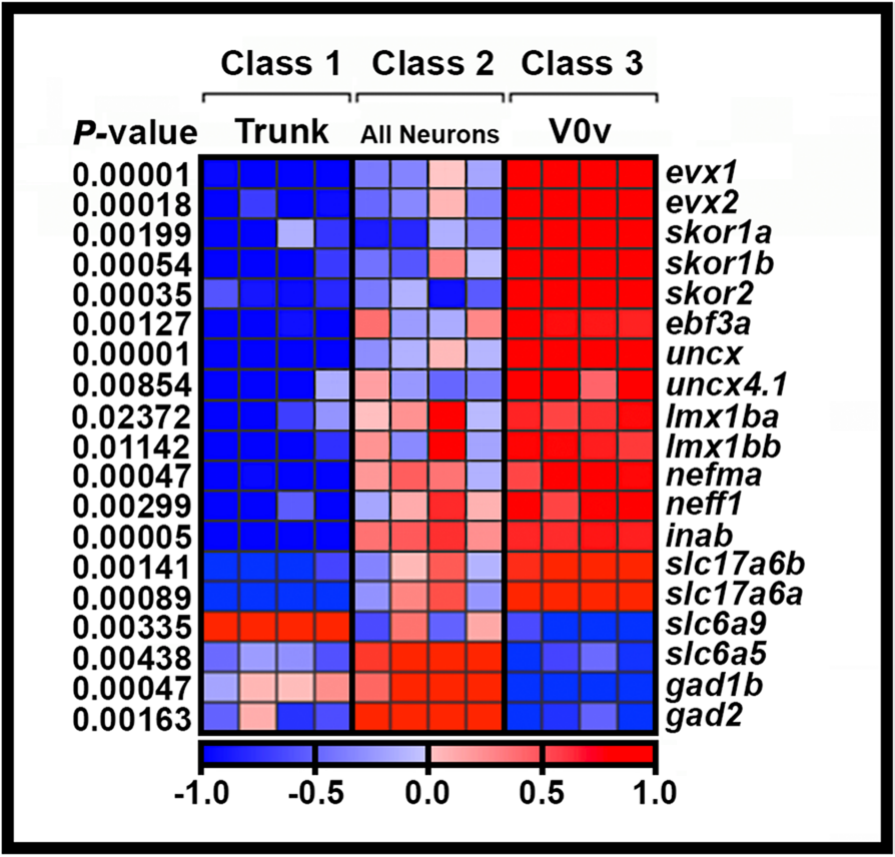
Transcriptional profiling of V0v spinal interneurons. Heatmap analysis of gene-expression profiling of 27 h V0v spinal cord interneurons. A three-class ANOVA analysis of differential expression was performed on different FAC-sorted populations of cells. Class 1: All trunk cells. Class 2: All post-mitotic spinal neurons. Class 3: V0v interneurons. Each column is a different biological replicate. Rows show relative expression levels for a single gene as normalized data transformed to a mean of 0, with standard deviation of + 1 (highly expressed, red) or -1 (weakly/not expressed, blue) sigma units. Adjusted *P-*values corrected for multiple testing are shown on the left-hand side. Expression profiles for positive control genes *evx1* and *evx2*, whose spinal cord expression is exclusive to V0v interneurons, are shown. The high level of expression of these genes in our V0v samples, compared to the other samples, confirms that we have successfully isolated V0v interneurons. Additional positive control genes *slc17a6a* and *slc17a6b*, confirm that V0v interneurons are excitatory (glutamatergic), whereas negative control genes *slc6a9, slc6a5, gad1b* and *gad2* show that V0v interneurons do not express either glycinergic or GABAergic inhibitory neurotransmitter pathway genes and that there is no contamination of our V0v samples with inhibitory neurons. The expression profiles for *slc17a6a, slc17a6b, slc6a9, slc6a5, gad1b* and *gad2* are reproduced from [14] as per the Creative Commons Attribution (CC BY) license at *Neural Development*

To identify additional transcriptional regulators that might be members of GRNs that specify V0v spinal interneuron neurotransmitter phenotypes, we FAC-sorted a pure population of these cells from 27 h post fertilization (h) *Tg(evx1:EGFP)*^*SU1*^ dissociated trunks, and compared the expression profiles of these cells with the profiles of all post-mitotic spinal neurons (isolated using *Tg(elavl3:EGFP*)) and all trunk cells, using a custom-designed microarray (Fig. 1). The microarray contained probes for all zebrafish genes that encode proteins containing at least one of the 483 InterPro transcriptional regulator domains identified by Armant and colleagues [13, 62]. It also contained probes for neurotransmitter synthesis and transporter genes that are often used to identify neurotransmitter phenotypes in neurons (see Methods and [13] for more details). From these analyses, we identified 11 transcriptional regulator genes enriched in V0v interneurons: *skor1a, skor1b, skor2*, *ebf3a*, *uncx*, *uncx4.1*, *lmx1ba*, *lmx1bb*, *nefma, neff1* and *inab* (Fig. 1; [13, 14]). *skor1a, skor1b, skor2*, *ebf3a*, *uncx, uncx4.1, lmx1ba* and *lmx1bb*, all encode transcription factors [13, 63–68]. In contrast, *nefma*, *neff1* and *inab* encode Neuronal Intermediate Filament (NIF) proteins [69, 70]. NIF proteins are not considered classical transcription factors, but they contain an InterPro transcriptional regulator domain and, thus, could function as transcriptional regulators in GRNs.

We have previously analyzed *lmx1ba* and *lmx1bb* expression and function in V0v interneurons [13]. To investigate the other genes, we performed an in situ hybridization time-course of *skor1a, skor1b, skor2*, *ebf3a, uncx, uncx4.1, nefma, neff1* and *inab* expression in WT embryos to further confirm that they are expressed in the V0v region of the spinal cord. We analyzed 17, 20, 24, 36 and 48 h, as, within the spinal cord, *evx1* and *evx2* are expressed exclusively by V0v interneurons at all these time points, and all the data so far suggest that transcription factor genes important for specifying functional characteristics of spinal interneurons are expressed during these key stages of development (e.g. [5, 12–14, 37, 71, 72]). Given that duplicated genes retained from whole genome duplication events (known as ohnologs) are often expressed in similar domains and may be functionally redundant, we also analyzed the spinal cord expression of *inaa, ebf3b* and *nefmb,* ohnologs of *inab*, *ebf3a* and *nefma* respectively. *inaa* did not show statistically significant differential expression in V0v interneurons in our microarray analysis (*P* > 0.17; Supp. Figure 1A’), and probes for *nefmb* and *ebf3b* were not present on the microarray because these genes were not accurately annotated in the Zv8 version of the zebrafish genome used for microarray construction. *neff1* does not have an ohnolog in zebrafish (http://ohnologs.curie.fr/).

**Fig. 2:**
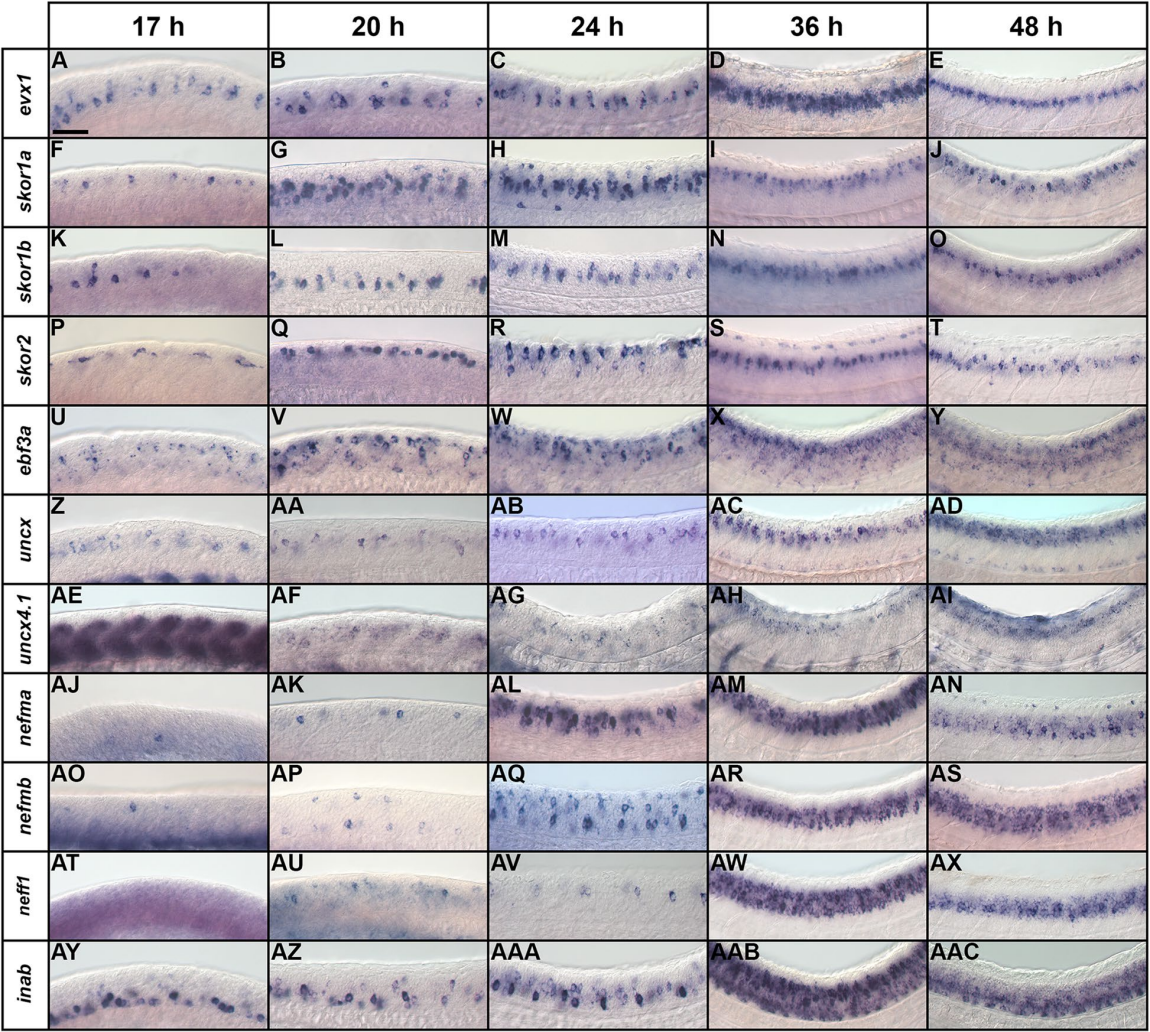
Temporal expression profiles of V0v candidate genes in zebrafish spinal cord. (**A**-**AAC**) Lateral views of (**A**-**E**) *evx1*, (**F**-**J**) *skor1a,* (**K**–**O**) *skor1b*, (**P**–**T**) *skor2*, (**U**-**Y**) *ebf3a*, (**Z**-**AD**) *uncx*, (**AE**-**AI**) *uncx4.1,* (**AJ**-**AN**) *nefma,* (**AO**-**AS**) *nefmb*, (**AT**-**AX**) *neff1,* and (**AY**-**AAC**) *inab* expression in WT spinal cord at (**A**, **F**, **K**, **P**, **U**, **Z**, **AE**, **AJ**, **AO**, **AT**, **AY**) 17 h, (**B**, **G**, **L**, **Q**, **V**, **AA**, **AF**, **AK**, **AP**, **AU**, **AZ**) 20 h, (**C**, **H**, **M**, **R**, **W**, **AB**, **AG**, **AL**, **AQ**, **AV**, **AAA**) 24 h, (**D**, **I**, **N**, **S**, **X**, **AC**, **AH**, **AM**, **AR**, **AW**, **AAB**) 36 h, and (**E**, **J**, **O**, **T**, **Y**, **AD**, **AI**, **AN**, **AS**, **AX**, **AAC**) 48 h. Rostral, left. Dorsal, up. (**A**-**E**) *evx1* is exclusively expressed in V0v spinal interneurons at all developmental stages analyzed and is shown here as a reference. Scale bar: 50 µm

**Fig. 3:**
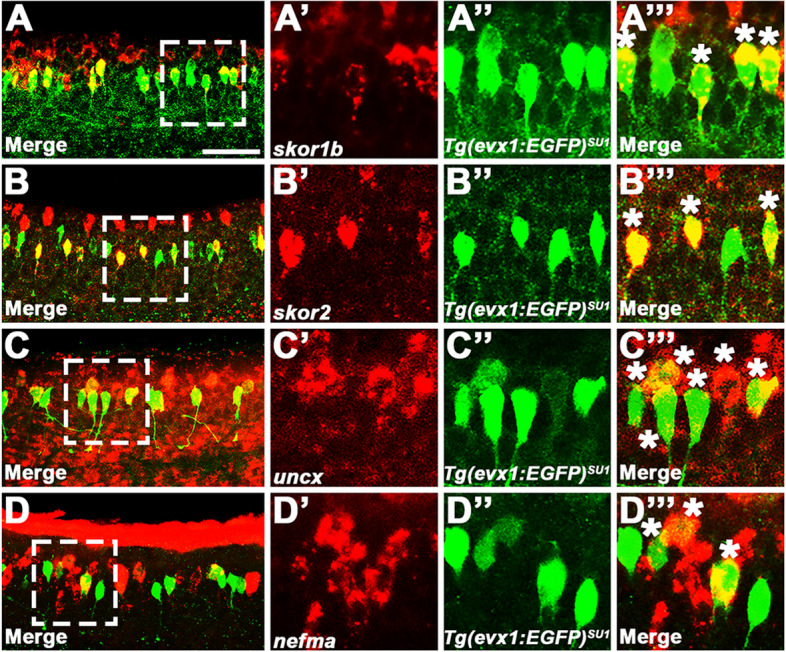
V0v candidate genes are co-expressed in subsets of V0v spinal interneurons. (**A**-**D’’’**) Lateral views of WT spinal cord at 27 h. Rostral, left. Dorsal, up. in situ hybridization for (**A’**) *skor1b*, (**B’**), *skor2*, (**C’**), *uncx*, and (**D’**) *nefma* genes is shown in red. (**A’’**, **B’’**, **C’’**, **D’’**) Immunohistochemistry for *Tg(evx1:EGFP)*^*SU1*^*,* which exclusively labels V0v spinal interneurons, is shown in green. (**A**, **A’’**’, **B**, **B’’’**, **C**, **C’’’**, **D**, **D’’’**) Merged images. (**A**, **B**, **C**, **D**) Maximum intensity projection images. (**A’**-**A’’’**, **B’**-**B’’’**, **C’**-**C’’’**, **D’**-**D’’’**) High-magnification single confocal planes of the region indicated by white dotted boxes in **A**, **B**, **C** and **D**. Similar *skor2* results were also reported in [14]. We are showing additional *skor2* data here to demonstrate reproducibility of our co-expression experiments, and for ease of comparison with the *skor1b, uncx* and *nefma* data. White asterisks indicate double-labelled V0v interneurons. Cells that are green and not red could be V0v interneurons that do not express the gene in question, or V0v interneurons with low expression, not revealed in these experiments, of the gene detected in red. We often detect fewer cells expressing a particular gene in double-labelling experiments where the mRNA is detected with a red fluorophore, than in single in situ hybridization experiments where the mRNA is detected with NBT/BCIP (viewed as an opaque blue stain under visible light), suggesting that the weakest-expressing cells may not be detected in the former, probably due to the prolonged processing of samples necessitated by fluorescent double-labelling experiments, which can affect the stability of target mRNA molecules, and the lower sensitivity of the red label. Therefore, we cannot conclude for certain that single-labelled EGFP-positive cells, do not express the gene detected in red. Scale bar: (**A**,** B**,** C**,** D**) 50 µm, (**A’-A’’’**, **B’-B’’’**,** C’-C’’’**,** D’-D’’’**) 20 µm

We found that *inaa* and *ebf3b* are not expressed in the spinal cord at any of the stages examined (Supp. Figure 1A, C and data not shown). In contrast, all the other genes are expressed in the region where V0v cells are located (middle of the dorsal–ventral spinal cord axis, see *evx1* expression Fig. 2A-E), during at least some of these crucial developmental stages. However, unlike *evx1* and *evx2*, all of these genes are also sometimes expressed in other dorsal–ventral regions of the spinal cord, demonstrating that they are also expressed by at least one additional spinal cord cell type (Fig. 2). Even *skor1b*, which is mainly expressed in the V0v domain, is also expressed by a few cells dorsal to V0v interneurons (Fig. 3A).

With respect to the three *skor* genes, only *skor1b* is expressed in the V0v spinal cord region at 17 h (Fig. 2A & K). *skor1a* (Fig. 2F) and *skor2* (Fig. 2P) are both expressed in the spinal cord at this stage but in a more dorsal location. *skor1b* continues to be expressed in a similar dorsal–ventral spinal cord region to *evx1* at 20 h, 24 h, 36 h and 48 h (Fig. 2B-E & L-O). By 20 h, *skor1a* is also expressed in the V0v spinal cord region*,* although it is still expressed in additional regions (Fig. 2G). In contrast, *skor2* is still only expressed in the dorsal spinal cord (Fig. 2Q). By 24 h, all three *skor* genes are expressed in a similar spinal cord region to *evx1* and this expression persists through 48 h (Fig. 2C-E, H-J, M–O & R-T). At 24 h, *skor1a* is still expressed in the dorsal spinal cord and it is also transiently expressed in a subset of ventral spinal cord cells (Fig. 2H). Expression in both these domains is lost by 36 h (Fig. 2I). *skor2* is also still expressed in the dorsal spinal cord at 24 h (Fig. 2R) but this dorsal expression begins to diminish by 36 h (Fig. 2S-T). Taken together, these data suggest that these *skor* genes have distinct temporal patterns of expression in the V0v domain, with *skor1b* expression preceding *skor1a* and *skor2* expression by 3 h and 7 h respectively. This raises the possibility that these genes have epistatic relationships with each other and/or function in different aspects of V0v cell development.

*ebf3a* and *uncx* are expressed in a similar dorsal–ventral region of the spinal cord to *evx1* at all the stages that we examined (Fig. 2A-E, U-Y & Z-AD). However, *ebf3a* is consistently expressed in more cells than *evx1*, suggesting that it is expressed by other cell types, in addition to V0v interneurons (Fig. 2A-E & U-Y). In contrast, *uncx* is only expressed by more cells than *evx1*, including cells in the ventral-most spinal cord, from 36 h onward (Fig. 2D-E & AC-AD). As previously described, *uncx4.1* is strongly expressed in the somites at 17 h (Fig. 2AE, [68]). Nittoli and colleagues documented *uncx4.1* expression in somites and brain at several different developmental stages but did not report spinal cord expression at any of the stages that they examined [68]. Initially, we also did not detect spinal cord expression in our in situ hybridization experiments. However, when we used the molecular crowding agent Dextran Sulphate (see Methods), we were able to detect weak spinal cord expression in the V0v domain of the spinal cord at 20 h and 24 h (Fig. 2AF-AG). At 36 h we no longer detect expression in this domain, but instead there is weak expression in the dorsal spinal cord, which persists at 48 h (Fig. 2AH-AI). While we cannot rule out the possibility that *uncx4.1* may have important functions in V0v interneuron specification, it seems unlikely, given this limited temporal expression in the V0v domain. On the other hand, *ebf3a* and *uncx,* like *skor1b,* are expressed in the V0v domain at all of the stages that we examined, suggesting that these three genes may have important roles in the same aspects of V0v development.

The NIF genes, *nefma*, *nefmb*, *neff1* and *inab* are all expressed in a similar dorsal–ventral region of the spinal cord to *evx1* during at least some stages of development, as well as additional spinal cord domains. At 17 h, *nefma* and *nefmb* are expressed in only a very small number of spinal cord cells, and *neff1* is not expressed at all (Fig. 2AJ, AO & AT). By 20 h*, neff1* is expressed in a few dorsal spinal cord cells (Fig. 2AU), which is very similar to both *nefma* (Fig. 2AK) and *nefmb,* although *nefmb* is also expressed in some ventral spinal cord cells (Fig. 2AP). By 24 h, *nefma*, *nefmb* and *neff1* are expressed in the V0v spinal cord domain, although *neff1* is still only expressed by a few cells (Fig. 2C, AL, AQ & AV)*.* All three of these genes continue to be expressed in this domain at 36 h and 48 h, although at these stages they are clearly expressed by more cells than *evx1* (Fig. 2D-E, AM-AN, AR-AS & AW-AX), suggesting that they are expressed in additional spinal cell types. In contrast to these other NIF genes, *inab* is expressed in a similar dorsal–ventral region of the spinal cord to *evx1* at all the developmental stages that we analyzed (Fig. 2A-E & AY-AAC). However, from 36 h onwards, *inab* is very broadly expressed in the spinal cord suggesting that while its earlier expression may be more specific, at 36 and 48 h it is expressed by the majority of post-mitotic spinal cells (Fig. 2AAB-AAC). The temporal expression pattern of *inab* in the V0v domain is, therefore, similar to that of *skor1b, ebf3a* and *uncx*. In contrast, the delayed onset of *neff1, nefma* and *nefmb* expression in the V0v domain, some 7 h later, is similar to that of *skor2.*

We also performed fluorescence in situ hybridization, for a subset of the genes that had the strongest expression in the single in situ hybridization experiments, in *Tg(evx1:EGFP)*^*SU1*^ embryos, in which EGFP spinal cord expression is exclusively in V0v cells [14]. These double-staining data confirm that *skor1b*, *skor2*, *uncx* and *nefma* are expressed by V0v interneurons (double-labelled cells) as well as non-V0v spinal cells (red but not green cells; Fig. 3 and also see [14] for different complementary data for *skor2*).

### *skor1a*,* skor1b*, *skor2* and *ebf3a* require Evx1/2 for their expression in the V0v spinal cord domain

**Fig. 4:**
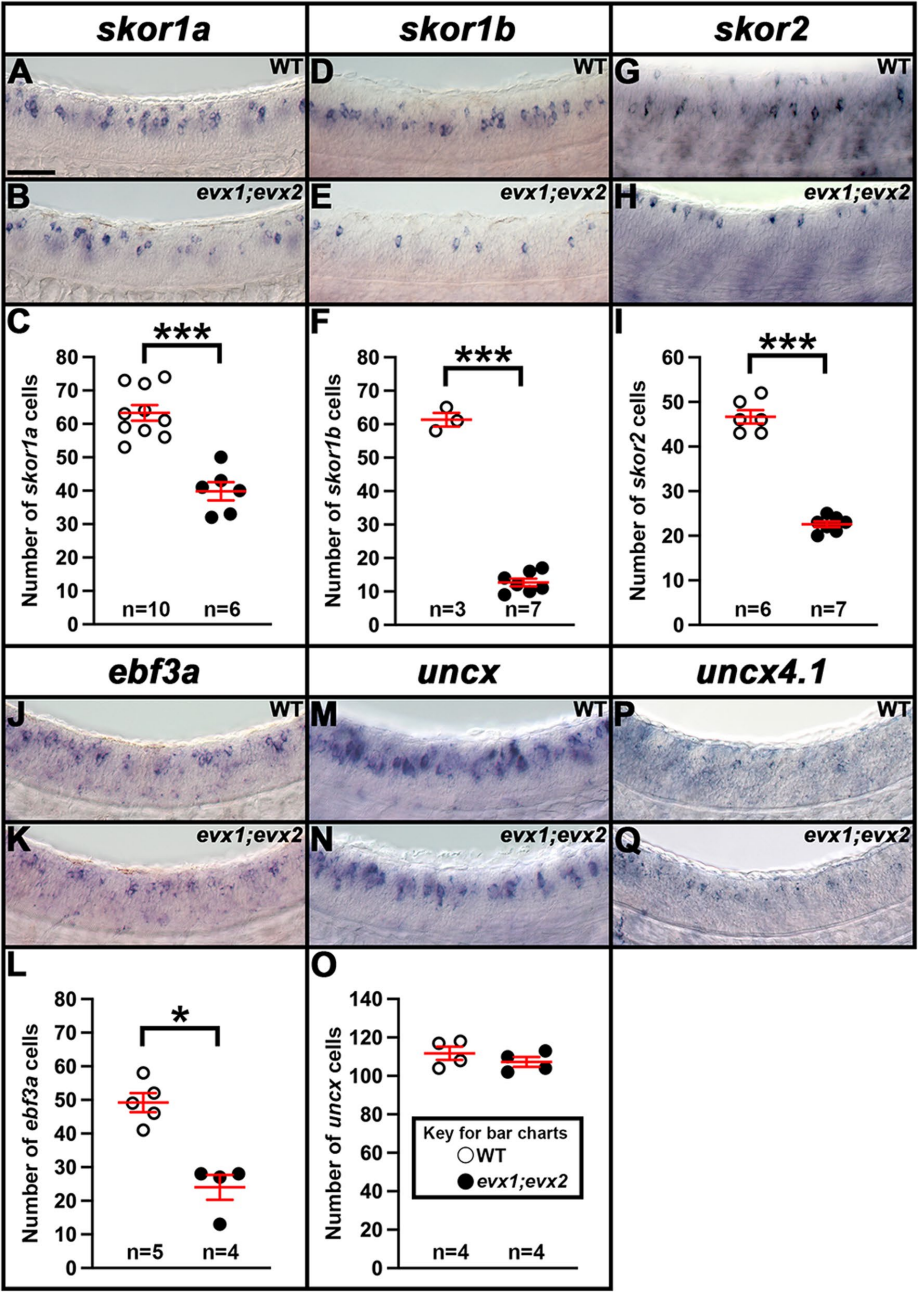
Expression of *skor1a, skor1b, skor2, ebf3a*, *uncx* and *uncx4.1* in Zebrafish *evx1;evx2* double mutant and WT embryos. (**A**,** B**,** D**,** E**,** G**,** H**,** J**, **K**,** M**,** N**, **P**, **Q**) Lateral views of (**A**,** D**,** G**,** J**,** M**, **P**) WT and (**B**, **E**,** H**,** K**,** N**,** Q**) *evx1*^*i232;i232*^*;evx2*^*sa140;sa140*^ double mutant embryos (labeled *evx1;evx2*) at 30 h. Rostral, left. Dorsal, up. (**C**,** F**,** I**,** L**,** O**) Number of cells expressing (**C**) *skor1a*, (**F**) *skor1b*, (**I**) *skor2*, (**L**) *ebf3a*, and (**O**) *uncx* in a precisely-defined spinal cord region adjacent to somites 6–10 at 30 h. We could not reliably count the number of cells expressing *uncx4.1*, due to the weak, punctate nature of the expression. Data are depicted as individual value plots with the *n-*values shown below. For each plot, the wider red horizontal bar indicates the mean number of cells, and the red vertical bar depicts the S.E.M. (both values are also listed in Table 1). All counts are an average of at least three embryos. Statistically significant comparisons are indicated with brackets and asterisks. *** *P* < 0.001. * *P* < 0.05. White circles indicate WT data and black circles indicate *evx1;evx2* double mutant data. All data were analyzed for normality using the Shapiro–Wilk test. Data in L is not normally distributed and so a Wilcoxon-Mann–Whitney test was performed. Data sets in **C**,** F**,** I** and **O** are normally distributed and so the F-test for equal variances was performed, followed by a type 2 Student’s *t*-test (for equal variances). *P*-values are provided in Table 1. (**C**,** F**,** I**,** L**,** O**) There is a statistically significant reduction in the number of spinal interneurons expressing *skor1a, skor1b, skor2* and *ebf3a*, but not *uncx,* in *evx1;evx2* double mutant embryos. (**A**,** B**) *skor1a*, (**D**,** E**) *skor1b* and (**P**, **Q**) *uncx4.1 *in situ hybridization experiments were performed with the molecular crowding reagent Dextran Sulfate. This was omitted for the (**G**,** H**) *skor2*, (**J**,** K**), *ebf3a* and (**M**,** N**) *uncx *in situ hybridization experiments. Scale bar: 50 µm

To investigate whether any of the other transcription factor genes expressed in the V0v spinal cord domain (Figs. 1, 2 and 3) are, like *lmx1ba* and *lmx1bb*, also downstream of Evx1/2 in V0v spinal interneurons [13], we examined the expression of these genes in *evx1;evx2* double mutants at 30 h (Fig. 4). Compared to WT siblings, we observed a statistically significant reduction in the number of cells expressing *skor1a* (Fig. 4A-C), *skor1b* (Fig. 4D-F), *skor2* (Fig. 4G-I), and *ebf3a* (Fig. 4J-L) in the V0v region of the spinal cord in *evx1;evx2* double mutants, suggesting that these genes require Evx1/2 for their expression in V0v interneurons (Table 1). We didn’t observe a complete loss of spinal expression of any of these genes, which is not surprising as they are all expressed by other spinal cells in additional to V0v interneurons (Figs. 2, 3 and discussion above). In contrast, despite being expressed in V0v interneurons (Fig. 3C-C’”), we did not detect any significant difference in the number of *uncx*-expressing spinal cells in *evx1;evx2* double mutants compared to WT siblings (Fig. 4M-O, Table 1). We were unable to reliably count spinal cells expressing *uncx4.1*, because the expression was so weak and punctate, even after using the molecular crowding reagent Dextran Sulfate (see Methods), and prolonged staining. However, we did not observe any differences in the spinal cord expression of this gene between WT and *evx1;evx2* double mutant embryos (Fig. 4P-Q).

### *nefma* and *neff1* are downstream of Evx1/2 in V0v interneurons at 30 h

**Fig. 5:**
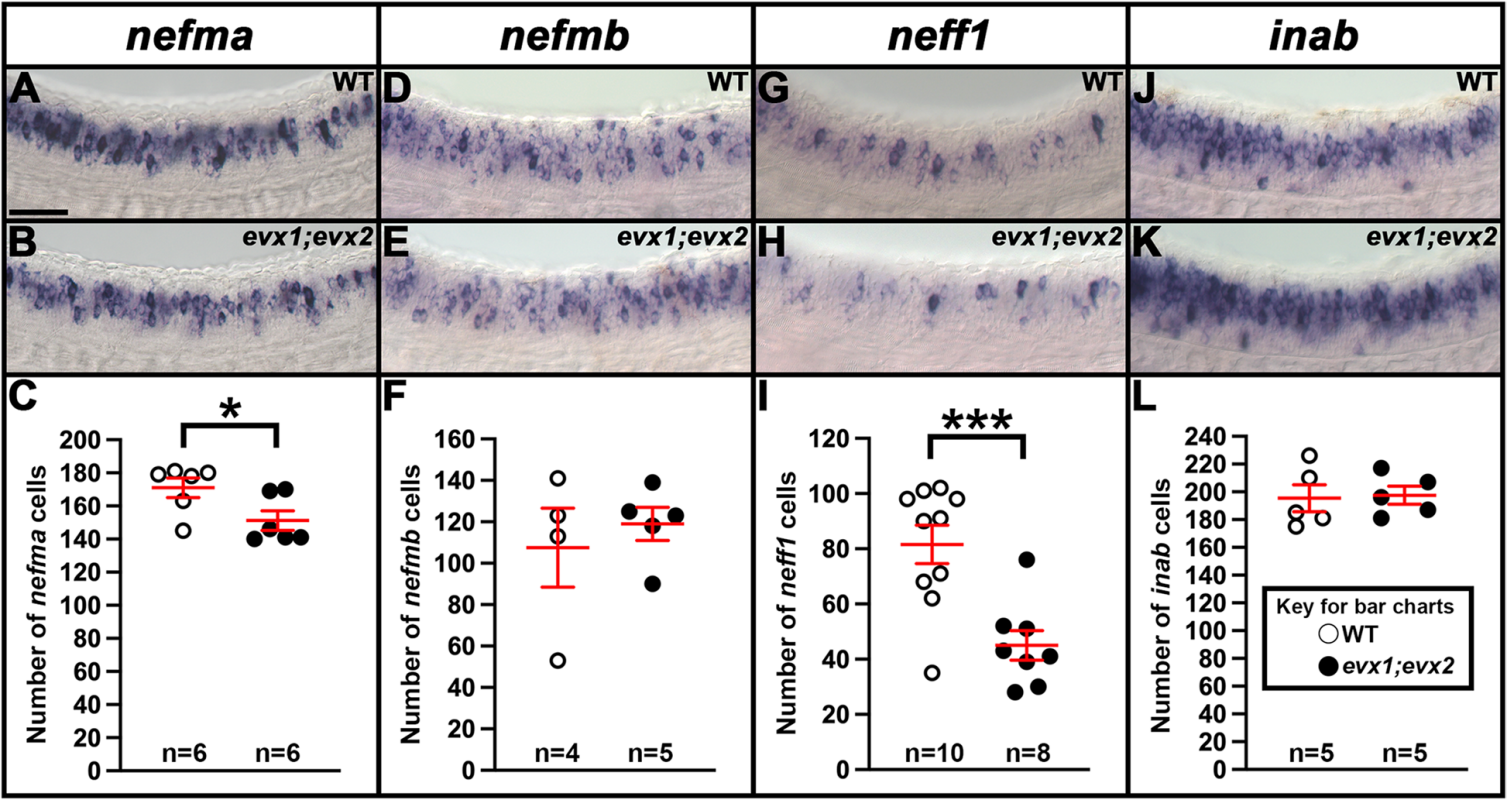
Expression of *nefma, nefmb, neff1* and *inab* in zebrafish *evx1;evx2* double mutant and WT embryos. (**A**,** B**,** D**,** E**,** G**,** H**,** J**,** K**) Lateral views of (**A**,** D**,** G**,** J**) WT and (**B**,** E**,** H**,** K**) *evx1*^*i232;i232*^*;evx2*^*sa140;sa140*^ double mutant embryos (labeled *evx1;evx2*) at 30 h. Rostral, left. Dorsal, up. (**C**,** F**,** I**,** L**) Number of cells expressing (**C**) *nefma*, (**F**) *nefmb*, (**I**) *neff1* and (**L**) *inab* in a precisely-defined spinal cord region adjacent to somites 6–10 at 30 h. Data are depicted as individual value plots and the *n-*values for each genotype are shown below. For each plot, the wider red horizontal bar depicts the mean number of cells, and the red vertical bar depicts the S.E.M. (mean numbers and S.E.M. values are listed in Table 1). All counts are an average of at least four embryos. Statistically significant comparisons are indicated with brackets and asterisks. *** *P* < 0.001. * *P* < 0.05. White circles indicate WT data and black circles indicate *evx1;evx2* double mutant data. All data were analyzed for normality using the Shapiro–Wilk test. Data in **C** is not normally distributed and so a Wilcoxon-Mann–Whitney test was performed. Data sets in **F**,** I** and **L** are normally distributed and so the F-test for equal variances was performed, followed by a type 2 Student’s *t*-test (for equal variances). *P*-values are provided in Table 1. (**C**, **I**) There is a statistically significant reduction in the number of spinal interneurons expressing *nefma* and *neff1*, but not (**F**, **L**) *nefmb* and *inab,* in *evx1;evx2* double mutant embryos. (**A**, **B**) *nefma* and (**G**, **H**) *neff1 *in situ hybridization experiments were performed with the molecular crowding reagent Dextran Sulfate. This was omitted for the (**D**, **E**) *nefmb* and (**J**, **K**) *inab *in situ hybridization experiments. Scale bar: 50 µm

We also investigated whether any of the NIF genes that are expressed in the V0v spinal cord domain (Figs. 1, 2 and 3) are downstream of Evx1/2 in V0v spinal interneurons. Compared to WT embryos, we detected a statistically significant reduction in the number of both *nefma*-expressing cells and *neff1*-expressing cells in 30 h *evx1;evx2* double mutants compared to WT embryos (Fig. 5A-C, G-I, Table 1). In contrast, we did not find statistically significant differences in the number of cells expressing *nefmb* or *inab* (Fig. 5D-F, J-L, Table 1).

As we had identified three NIF genes in our microarray analyses (*nefma, neff1* and *inab*), for completeness, we decided to also examine the expression of the remaining two zebrafish NIF genes, *nefla* and *neflb*. Our microarray data suggested that these were both expressed in the spinal cord but not in V0v interneurons (Supp. Figure 2A). Consistent with this, we found no change in the number of cells expressing either *nefla* or *neflb* in *evx1;evx2* double mutants compared to WT siblings (Supp. Figure 2B-G, Table 1).

### Single-cell RNA-sequencing (scRNA-seq) analysis identifies distinct V0v sub-populations in WT embryos and *evx1/2* mutants

While we would expect Evx1 and Evx2 to act cell autonomously, as they are both transcription factors, it is still important to confirm that the spinal cells that lose expression of particular genes in *evx1;evx2* double mutants are, indeed, V0v interneurons. Therefore, we FAC-sorted EGFP-positive V0v spinal interneurons from the progeny of an incross of heterozygous *evx1;evx2* double mutant fish that were homozygous for *Tg(evx1:EGFP)*^*SU2*^ and performed single cell RNA sequencing (scRNA-seq). We cannot distinguish *evx1;evx2* single or double mutant embryos from WT siblings morphologically, so our V0v interneurons were collected from all of the different genotypes generated from this cross. However, we were able to distinguish mutant cells from WT cells in our data analyses, based on each cell’s individual gene expression profile (see Methods). We performed these analyses at 48 h because EGFP is expressed in significantly more V0v interneurons in 48 h *Tg(evx1:EGFP*)^*SU2*^ embryos than at earlier developmental stages [14], enabling us to capture a larger number of rare double mutant cells. (Only 1/16 embryos from an incross of *evx1;evx2* heterozygous parents will be double mutants, and, in order to maintain mRNA integrity, we can only dissect trunks for a limited amount of time for each experiment. See Methods for more details). This also enabled us to investigate the phenotype of *evx1;evx2* mutant cells at an additional stage of development.

**Fig. 6:**
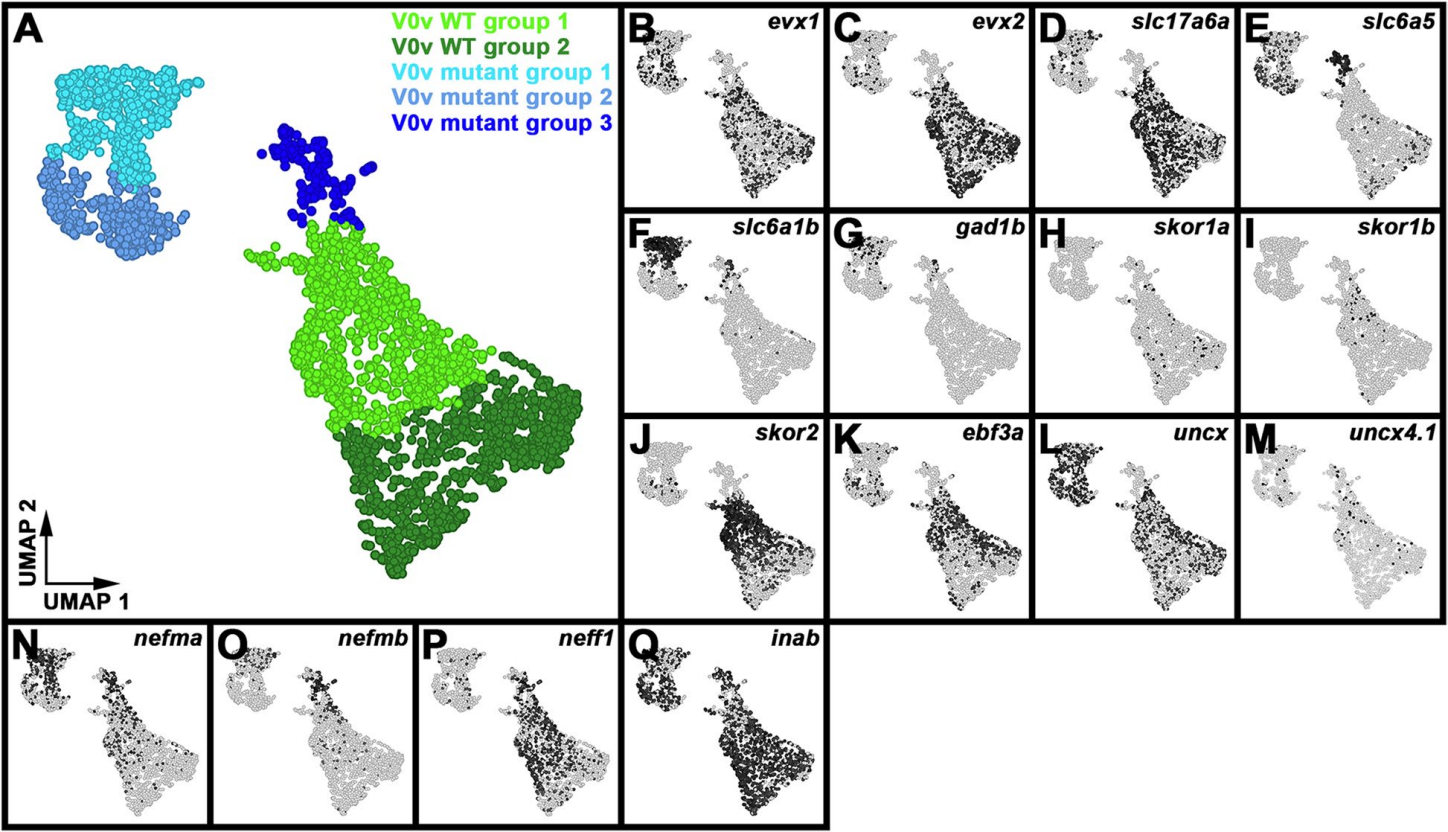
Single-cell RNA-seq analysis of WT and *evx1*/2 mutant V0v interneurons identifies five distinct clusters of cells. (**A**) 2D UMAP plot of 48 h post-mitotic V0v spinal interneuron single-cell RNA-seq atlas (2860 cells). Cells were obtained from 48 h embryos produced from an incross of *evx1*^*i232/*+^*;evx2*^*sa140/*+^ heterozygous parents homozygous for *Tg(evx1:EGFP)*^*SU2*^. Clusters are color-coded by cell identity: V0v WT Group 1 (light green), V0v WT Group 2 (dark green), V0v Mutant Group 1 (turquoise), V0v Mutant Group 2 (light blue), and V0v Mutant Group 3 (dark blue). Cell fate assignments were deduced and extrapolated by comparing expression profiles of 48 h single-cell clusters with the molecular phenotypes of V0v spinal interneurons in WT and *evx1* and *evx2* single and double mutant embryos [14]. (**B**-**Q**) 2D UMAP plots of differential gene expression between cell clusters. Black shows high levels of expression, light grey shows low levels of expression. All expression data have been normalized (see Methods). (**B**-**D**) Many of the cells in both WT clusters express (**B**) *evx1* and/or (**C**) *evx2,* as well as the glutamatergic marker (**D**) *slc17a6a*. (**B**-**G**) *evx1, evx2* and *slc17a6a* are all detected in fewer cells in Mutant Groups 1 and 2 and hardly any cells in Mutant Group 3. Many cells in the mutant clusters upregulate inhibitory markers, including (**E**) *slc6a5,* (**F**) *slc6a1b*, and (**G**) *gad1b*. (**H**) *skor1a* and (**I**) *skor1b* are not detected in many cells in this data set. (**H**) *skor1a* is expressed in a few WT Group 1 and 2 cells, as well as a couple of Mutant Group 3 cells and a Mutant Group 1 cell. (**I**) *skor1b* is predominantly detected in a few WT Group 1 cells. (**J**) In contrast, *skor2* is expressed at high levels in most V0v WT Group 1 cells, and it is also detected in multiple WT Group 2 cells and a small number of Mutant Group 2 cells. (**K**) *ebf3a* has a similar expression profile to *skor2*, except that its expression is also detected in a few Mutant Group 1 cells and slightly more Mutant Group 2 cells. (**L**) *uncx* is expressed by many cells in all the clusters except the Mutant Group 3 cluster. The highest proportions of *uncx*-expressing cells are in Mutant Groups 1 and 2 (56.58% (245/433) and 58.81% (217/369) respectively, compared to 42.44% (396/933) WT Group 1 cells, 32.79% (303/924) WT Group 2 cells, and 12.44% (25/201) Mutant Group 3 cells). (**M**) In contrast to *uncx*, *uncx4.1* is only expressed by several cells in each of the clusters. (**N**-**Q**) Of the neuronal intermediate filament genes, *inab* is expressed in all five clusters, but it is detected in slightly fewer cells in the mutant clusters. (**N**–**O**) *nefma* and *nefmb* are predominantly expressed by cells in WT and Mutant Group 1 clusters and the Mutant Group 3 cluster. (**P**) *neff1* is detected in most WT Group 1 cells, some WT Group 2 cells, several Mutant Group 3 cells, but hardly any Mutant Group 1 or 2 cells

Following FAC-sorting and scRNA-seq, we generated a dataset of 2860 V0v cells that passed stringent quality controls (see Methods). For improved visualization and interpretation of this single-cell atlas, we used Uniform Manifold Approximation and Projection (UMAP) plotting, since this preserves the global structure of the expression data. We manually inspected UMAP plots to assess clustering quality based on expression of known V0v spinal interneuron markers. Using these methods, we identified five distinct clusters of V0v interneurons (Fig. 6A). We visually compared the expression of different genes in these clusters and performed gene-specific analyses of differential expression using both Hurdle model and ANOVA statistical comparisons (see Methods for an explanation of why we used these statistical methods). Comparing the expression profiles of *evx1, evx2* and neurotransmitter phenotype genes in these five distinct clusters with our previously published data [14, 73], suggested that the two clusters with the highest expression levels of *evx1, evx2* and the excitatory (glutamatergic) transmembrane transporter gene *slc17a6a*, and the lowest expression levels of inhibitory neurotransmitter genes *slc6a5, slc6a1b* and *gad1b,* are WT V0v cells (Fig. 6B-G, Table 2). In contrast, the clusters with lower expression levels of *evx1, evx2* and *slc17a6a*, and higher expression levels of *slc6a5, slc6a1b* and *gad1b*, are most likely to be mutant V0v cells (Fig. 6B-G, Table 2). Mutant Group 3 is the smallest cluster of profiled cells, and the cluster with the most severe reduction in *evx1, evx2* and *slc17a6a* expression (Fig. 6A-D, Table 2) and the highest level of expression of the inhibitory (glycinergic) gene *slc6a5* (Fig. 6E*,* Table 2). This suggests that it contains double mutant cells, as we would expect these cells to have the most severe phenotype.

**Table 2:** Differential expression analysis of V0v candidate genes between WT and *evx1;evx2* mutant cell groups

**A**							
**Gene Symbol**	**Hurdle Model Fold-Change**						
	**Mutant 1 + 2 vs WT 1 + 2**	**Mutant 3 vs Mutant 1 + 2**	**Mutant 1 vs WT 1**	**Mutant 2 vs WT 2**	**Mutant 3 vs WT 1 + 2**	**WT 1 vs** **WT 2**	**Mutant 1 vs Mutant 2**
*evx1*	↓1.77***	↓2.45***	↓1.67*	↓1.86***	↓4.32***	↓1.03***	↑1.08
*evx2*	↓17.73***	↓1.50*	↓15.10***	↓20.31***	↓26.41***	↓1.61***	↓1.19**
*slc17a6a*	↓15.87***	↓1.04	↓14.50***	↓15.50***	↓16.51***	↑1.75***	↑1.87***
*slc6a5*	↑6.87***	↑28.60***	↑4.79***	↑8.39***	↑195.57***	↓1.91***	↓3.33***
*slc6a1b*	↑7.71***	↓1.54	↑201.72***	↑1.41***	↑5.01***	↑1.03	↑147.81***
*gad1b*	↑1.44***	↑1.50	↑3.72***	↑1.08**	↑2.17***	↓1.01	↑3.41***
*skor1a*	↓1.18**	↑1.17*	↓1.14	↓1.24*	↓1.00*	↓1.10*	N.C.
*skor1b*	↓1.09***	N.C.	N.C.	N.C.	↑1.05	↑1.37***	N.C.
*skor2*	↓43.94***	↓1.12	↓434.75***	↓3.12***	↓49.21***	↑90.90***	↓1.53***
*ebf3a*	↓4.24***	↓1.25	↓18.44***	↓1.18***	↓5.28***	↑9.15***	↓1.71**
*uncx*	↑3.94***	↓16.53***	↑2.56***	↑5.95***	↓4.21***	↑1.78***	↓1.31***
*uncx4.1*	↑1.10	↓1.02	↓1.05	↑1.18***	↑1.07	↑1.29***	↑1.04
*nefma*	↑7.23***	↑3.12***	↑10.66***	↑3.63***	↑22.47***	↑3.19***	↑9.34***
*nefmb*	↑1.27*	↑40.64***	↑1.96***	↑1.07	↑51.55***	↑1.98***	↑3.61***
*neff1*	↓8.12***	↑21.65***	↓22.08***	↓3.53***	↓2.68***	↑7.27***	↑1.16
*inab*	↓3.65***	↑1.25	↓6.43***	↓2.03***	↓2.92***	↓1.14***	↓3.61***
**B**							
**Gene Symbol**	**ANOVA Fold-Change**						
	**Mutant 1 + 2 vs WT 1 + 2**	**Mutant 3 vs Mutant 1 + 2**	**Mutant 1 vs WT 1**	**Mutant 2 vs WT 2**	**Mutant 3 vs WT 1 + 2**	**WT 1 vs** **WT 2**	**Mutant 1 vs Mutant 2**
*evx1*	↓1.07***	↓2.37***	↓1.24*	↑1.09**	↓2.54***	↑1.32	↓1.02
*evx2*	↓6.09***	↓2.39*	↓7.96***	↓4.98***	↓14.59***	↓1.05**	↓1.68
*slc17a6a*	↓5.33***	↑1.20	↓4.47***	↓7.53***	↓4.46***	↑1.52***	↑2.57***
*slc6a5*	↑5.39***	↑6.94***	↑6.21***	↑5.11***	↑37.44***	↓2.94***	↓2.42***
*slc6a1b*	↑138.60***	↓2.52***	↑186.10***	↑13.82***	↑54.94***	↑2.63	↑35.38***
*gad1b*	↑34.98***	↑1.17	↑74.42***	↑4.31*	↑41.08***	↓1.29	↑13.44***
*skor1a*	↓6.35***	↑9.00**	↓5.28*	↓7.44**	↑1.42	↓1.38	↑1.02
*skor1b*	↓12.24***	↑10.27***	↓22.80***	↓1.68	↓1.19	↑13.58***	N.C.
*skor2*	↓16.64***	↓10.94	↓141.98***	↓1.63***	↓182.04***	↑10.43***	↓8.35***
*ebf3a*	↓3.15***	↓2.16	↓8.64***	↑1.13	↓6.80***	↑4.03***	↓2.43**
*uncx*	↑2.25***	↓4.51***	↑1.63***	↑3.18***	↓2.00***	↑1.49***	↓1.31
*uncx4.1*	↑1.26	↑1.18	↓1.24	↑2.85*	↑1.49	↑3.51***	↓1.01
*nefma*	↑3.94***	↑2.06***	↑3.16***	↑11.32***	↑8.11***	↑9.38***	↑2.62***
*nefmb*	↑1.64***	↑18.45***	↑1.66***	↑1.42	↑30.22***	↑10.09***	↑11.80***
*neff1*	↓9.56***	↑19.31***	↓10.92***	↓6.68***	↑2.02***	↑3.46***	↑2.12
*inab*	↓1.43***	↑1.21	↓2.22***	↓1.02***	↓1.19***	↑1.13	↓1.92***

Gene-specific analyses of differential expression, created through (**A**) Hurdle model and (**B**) ANOVA statistical comparisons between distinct cell clusters in our 48 h *evx1*^*i232/*+^*;evx2*^*sa140/*+^ heterozygote incross single-cell atlas (see Fig. 6A and also Methods for experimental details and rationale for using both statistical methods). The Hurdle model is generally the most statistically robust method for these analyses, if there are enough cells in each group. In contrast, ANOVA usually performs better when the numbers of cells being compared are very small (see Methods for more information). We are providing the data for both methods for completeness and comparison. Column 1 shows the gene symbol. Columns 2–8 show fold-change values. ↑ = fold-change increase, ↓ = fold-change decrease in the antecedent (first) population compared to the consequent (second) population in each comparison. Statistically significant (*P* < 0.05) values are indicated in bold. *** *P* < 0.001, ** *P* < 0.01, * *P* < 0.05*.* N.C. = Not Calculated. The differential expression analysis could not be calculated. In the case of *skor1b*, this is because there is no expression of this gene in some of the clusters. Mutant Groups 1 + 2 combined, Mutant Group 3, Mutant Group 1, Mutant Group 2, Mutant Group 3, WT Group 1, and Mutant Group 1 are the antecedent populations for columns 2, 3, 4, 5, 6, 7 and 8 respectively. WT Groups 1 + 2 combined, Mutant Groups 1 + 2 combined, WT Group 1, WT Group 2, WT Groups 1 + 2 combined, WT Group 2 and Mutant Group 2 are the consequent populations for columns 2, 3, 4, 5, 6, 7 and 8 respectively. Additional data for each comparison are available in Supp. Data Tables 2 (Hurdle model data) and 3 (ANOVA data)

Our previously published data suggest that the phenotypes of *evx1* and *evx2* single mutants and *evx1;evx2* double mutants differ only in their severity / penetrance. For example, both single mutants lose expression of *evx2, slc17a6* and *skor2* in some spinal cord cells, whereas in the double mutants, more cells lose expression of these genes, and we also see a statistically significant reduction in the number of spinal cord cells expressing *evx1* [14]. (Due to the molecular nature of the *evx1* and *evx2* mutations, we can still detect mRNA for both these mutated genes). Given the similarity of the single and double mutant phenotypes, before we performed these scRNA-seq analyses, we were not sure whether all mutant cells would cluster together, or whether we would see distinct clusters of less severe and more severe mutant cells. It is theoretically possible that what distinguishes the Mutant Group 1 cluster from the Mutant Group 2 cluster is the severity of the mutant phenotype. However, the data appear to be inconsistent with this hypothesis. We do not see a statistically significant difference in *evx1* expression between Mutant Group 1 and Mutant Group 2, and while *evx2* is expressed at slightly higher levels in Mutant Group 2, this difference is only statistically significant when we use the Hurdle model (Table 2). The most striking difference between the Mutant Group 1 and Mutant Group 2 clusters is the much higher, statistically significant expression of inhibitory marker *slc6a1b* in Mutant Group 1 cells (Table 2). It is not clear though what the functional significance of this difference is. Statistically, Mutant Group 1 cells also have more expression of a different inhibitory marker *gad1,* than Mutant Group 2 cells, but statistically they also have less expression of the inhibitory marker *slc6a5,* and more expression of the excitatory marker *slc17a6a* (Table 2). Based on these data we think that it is more likely that, as discussed below, the Mutant Group 1 and 2 clusters are mutant versions of the two molecularly distinct WT clusters that we have identified.

The two WT clusters contain 933 cells (Group 1) and 924 cells (Group 2) respectively, whereas the mutant clusters contain 433 cells (Group 1), 369 cells (Group 2), and 201 cells (Group 3) respectively. Assuming that WT and mutant cells are equally likely to survive cell dissociation and FAC-sorting and end up in our dataset, we would expect a ratio of 1609:1251 cells (9:7) for WT cells compared to mutant cells, whereas what we observe is 1857:1003. A Chi-squared test shows that there is a statistically significant difference between these frequencies (*P* < 0.001). This suggests that either mutant cells were more fragile and, therefore, had a higher probability of not making it into our data set, or some of the mutant cells are contained in what we have defined as the WT clusters. Given that, as discussed above, the phenotypes of *evx1* and *evx2* single mutants are not completely penetrant [14], the latter explanation would not be surprising. Consistent with this, we detected expression of the inhibitory marker *slc6a5* in a few cells in both WT clusters (Fig. 6E). *slc6a1b* is also detected in a very small number of both WT cell types, although interestingly, *gad1b* is not (Fig, 6F-G). Three-way differential gene expression shows that a few of the cells in WT Groups 1 and 2 that express *slc6a5* also express either *evx1*, *evx2* (white/grey cells, Supp. Figure 3C) or the excitatory marker *slc17a6a* (turquoise cells, Supp. Figure 3D), suggesting that they may be cells with a partial mutant phenotype. Although our scRNA-seq experiments were performed with high sequencing depth and transcriptome coverage (see Methods), considering the parameters of scRNA-seq, it should be noted that for all of these analyses we cannot rule out the small possibility that additional cells may express low, undetected, levels of a particular gene.

Similarly, if the Mutant Group 3 cells are double mutant cells, we would expect them to be 1/16^th^ of the total (179 cells), whereas there are 201 cells in this cluster. In this case though, a Chi-squared test does not find a statistically significant difference between the expected and observed frequencies (*P* = 0.09). Therefore, the numbers of cells that we observe in Mutant Group 3 is consistent with the hypothesis that this cluster contains double mutant cells. It is also possible that the number of cells in this group is slightly higher than expected, because some single mutant cells with a severe phenotype (for example a subset of the cells that have 3 out of 4 mutant alleles) are included in this group.

### *skor1a*,* skor1b*,* skor2*,* ebf3a*,* uncx*,* uncx4.1*,* nefma*,* neff1* and *inab* are expressed in V0v spinal interneurons at 48 h

All nine of the genes that we identified as being expressed in 27 h V0v spinal interneurons using microarray expression-profiling (Fig. 1), and in situ hybridization (Figs. 2–5), are still expressed in WT V0v interneurons at 48 h (Fig. 6H-N, P-Q). However, we only detected *skor1a*, *skor1b* and *uncx4.1* expression in a few V0v cells at this stage (Fig. 6H-I, M, Table 2), suggesting that these genes may be expressed by fewer V0v interneurons at later stages of embryogenesis, or that they may be expressed at low levels, and hence drop out of the profiles of some cells. *nefmb* was also only detected in a small number of cells in the two WT Groups, although it was expressed by more cells in Mutant Groups 1 and 3 (Fig. 6O, Table 2). In contrast, *skor2*, *ebf3a*, *uncx*, *nefma*, *neff1* and *inab* (Fig. 6J-L, N, P-Q, Table 2) expression was detected in many V0v cells. Interestingly, we detected *skor1b*, *skor2*, *ebf3a*, *uncx*, *uncx4.1*, *nefma*, *nefmb* and *neff1* expression in more cells in the UMAP plots, and at statistically-significantly higher levels in our gene-specific analyses of differential expression, in WT Group 1 compared to WT Group 2 (Fig. 6I-P, Table 2A). Using the Hurdle model (as both WT clusters contain a relatively large number of cells, and so the variance can be effectively modelled (please see Methods for further explanation)) this increase is most dramatic for *skor2*, but also substantial for *neff1* and *ebf3a*. The other increases are more modest, although still statistically significant. In contrast, *skor1a* and *inab* were expressed at slightly higher, statistically significant, levels in WT Group 2 cells than in WT Group 1 cells (Fig. 6H, Q, Table 2A).

Consistent with our in situ hybridization data at 30 h (Figs. 4–5, Table 1), our 48 h scRNA-seq data suggest that expression of *skor1a*, *skor1b*, *skor2*, *ebf3a* and *neff1* in V0v interneurons requires Evx1/2. We detected fewer V0v cells expressing *skor1a*, *skor1b*, *skor2*, *ebf3a* and *neff1* in the three mutant clusters, with the exception that *neff1* is still expressed in many Mutant Group 3 cells (Fig. 6H-K, P). These results are also confirmed by our gene-specific analyses of differential expression, although no statistical analyses could be performed for some of the *skor1b* comparisons as no cells expressing it were detected in Mutant Groups 1 or 2. In addition, the difference between Mutant Group 1 and WT Group 1 is not statistically significant for *skor1a,* using the Hurdle model (Table 2A). In general, statistical analyses using both Hurdle and ANOVA models give similar results, although there are some subtle differences and in most of these comparisons, the Hurdle model is probably more reliable because of the increased efficiency of variance modelling over ANOVA when there are large cell numbers in the groups under comparison (see Methods).

In contrast, for *uncx, nefma*, *nefmb* and *inab*, we identified differences between our in situ hybridization data at 30 h (Figs. 4 and 5, Table 1) and our 48 h scRNA-seq data (Fig. 6L, N–O, Q, Table 2). For example, while we detected no change in the number of cells expressing *uncx,* in *evx1;evx2* double mutant embryos compared to WT siblings in our 30 h in situ hybridization experiment (Fig. 4M-O, Table 1), in our 48 h scRNA-seq data, we found that *uncx* is expressed in a higher proportion of cells, and at statistically-significant higher levels, in Mutant Groups 1 and 2 than the two WT Groups (Fig. 6L, Table 2)*.* In contrast, there are hardly any cells expressing *uncx* in Mutant Group 3 (Fig. 6L, Table 2)*.* Similarly*,* at 30 h (Fig. 5A-F, Table 1), we detected a slight, but statistically significant, reduction in the number of spinal cells expressing *nefma* in *evx1;evx2* double mutants, but no change in the number of spinal cells expressing *nefmb*, whereas in our 48 h scRNA-seq experiment, we detected a higher proportion of *nefma*- and *nefmb-*expressing cells, and a statistically significant higher level of expression of these genes in all three mutant clusters (Fig. 6N-O and Table 2, the comparison between Mutant Group 2 and WT Group 2 is not statistically significant for *nefmb*). In addition, at 30 h, we did not find a difference in the number of spinal cord cells expressing *inab* in *evx1;evx2* double mutants (Fig. 5J-L, Table 1), whereas in our 48 h data, *inab* is expressed in a lower percentage of cells in the mutant clusters than in the WT clusters (Fig. 6Q, as this result is harder to see from the 2D UMAP plot alone, we confirmed it by quantifying the number of *inab*-expressing cells per cluster in a dotplot showing the number of *inab* reads detected per cell per group (Table 3)) and there is also a statistically significant reduction in its expression in the mutant groups compared to the WT groups (Table 2). Our data for *uncx4.1* is less conclusive than for these other genes as we were not able to count the number of spinal cord cells expressing this gene at 30 h. However, we didn’t see any obvious change in its expression at this stage, which is consistent with our 48 h data, where the only statistically significant change that we see in mutant cells, is for Mutant Group 2 cells compared to WT Group 2 cells. Our scRNA-seq data suggests that very few V0v interneurons express *uncx4.1,* in any of the different clusters at 48 h. Although, based on the UMAP plots and differential gene expression analyses, there is statistically slightly more expression in WT Group 1 than WT Group 2. Taken together, these data suggest that Evx1/2 may repress *uncx4.1* expression slightly in WT Group 2.

**Table 3:** Number of cells expressing particular genes in the different 48 h V0v clusters

V0v Cluster	WT 1	WT 2	Mutant 1	Mutant 2	Mutant 3
Number of cells in cluster	**933**	**924**	**433**	**369**	**201**
% *inab-*expressing cells	68.49% (639)	70.89% (655)	42.96% (186)	58.54% (216)	49.75% (100)
% *gata2a*-expressing cells	0.11% (1)	0.32% (3)	0.23% (1)	0.00% (0)	10.95% (22)
% *gata3*-expressing cells	0.43% (4)	0.11% (1)	0.00% (0)	0.00% (0)	12.94% (26)
% *tal1*-expressing cells	0.75% (7)	0.43% (4)	0.46% (2)	0.54% (2)	15.92% (32)
% *sst1.1*-expressing cells	0.00% (0)	0.00% (0)	0.00%(0)	0.00% (0)	1.49% (3)
% *enlb-*expressing cells	0.11% (1)	0.43% (4)	0.00% (0)	0.27% (1)	28.36% (57)
% *dmrt3a*-expressing cells	0.21% (2)	0.54% (5)	0.23% (1)	0.27% (1)	13.93% (28)
% *lbx1a*-expressing cells	0.43% (4)	0.11% (1)	0.46% (2)	0.00% (0)	10.95% (22)
% *isl1a*-expressing cells	0.11% (1)	0.11% (1)	0.23% (1)	0.00% (0)	4.48% (9)
% *isl2a*-expressing cells	0.11% (1)	0.22% (2)	0.46% (2)	0.27% (1)	5.97% (12)
% *isl2b*-expressing cells	0.00% (0)	0.11% (1)	0.00% (0)	0.00% (0)	3.48% (7)
% *mnx1*-expressing cells	0.11% (1)	0.11% (1)	0.00% (0)	0.00% (0)	5.97% (12)
% *mnx2a*-expressing cells	0.00% (0)	0.00% (0)	0.00% (0)	0.00% (0)	3.48% (7)
% *mnx2b*-expressing cells	0.11% (1)	3.14% (29)	0.23% (1)	0.27% (1)	5.97% (12)
% *sim1a*-expressing cells	0.11% (1)	0.00% (0)	0.46% (2)	0.00% (0)	0.00% (0)
% *vsx2*-expressing cells	0.21% (2)	1.30% (12)	0.00% (0)	0.27% (1)	1.99% (4)
% *tlx3b*-expressing cells	0.00% (0)	0.00% (0)	0.00% (0)	0.00% (0)	0.50% (1)
% *foxp2*-expressing cells	2.36% (22)	1.30% (12)	3.23% (14)	4.88% (18)	2.99% (6)
% *barhl2*-expressing cells	0.00% (0)	0.00% (0)	0.00% (0)	0.27% (1)	0.00% (0)

Quantification of the number of cells expressing particular genes in WT Group 1 (column 2), WT Group 2 (column 3), Mutant Group 1 (column 4), Mutant Group 2 (column 5) and Mutant Group 3 (column 6) from dotplots showing the logarithmic number of reads for each gene detected per cell per group. First row shows the total number of cells per cluster. Additional rows indicate the percentage of these cells expressing each gene, with the number in parentheses

### scRNA-seq analysis identifies two distinct V0v WT clusters, each of which is most similar to a different mutant cluster

**Fig. 7:**
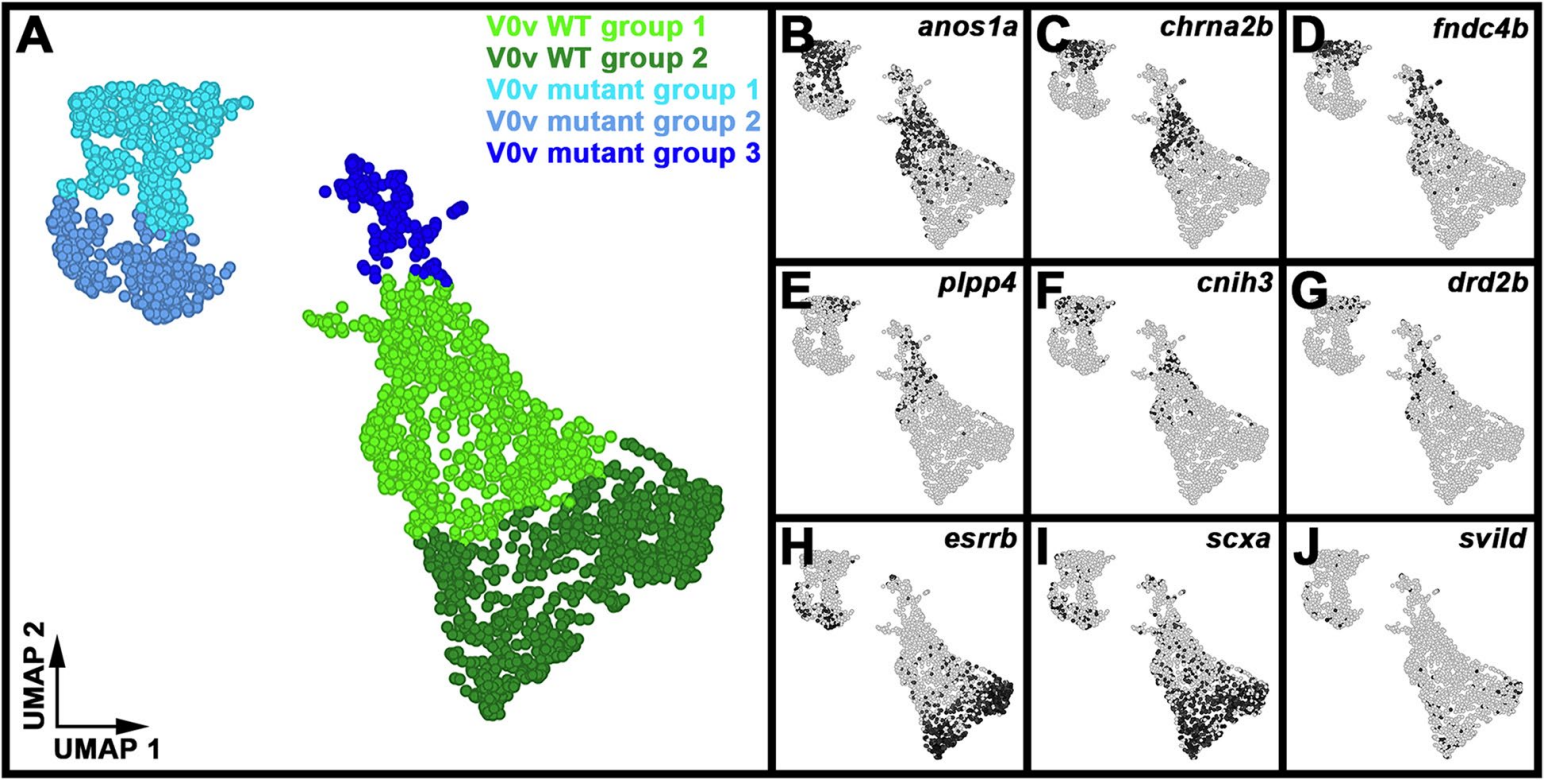
Differential gene expression identifies two distinct subsets of WT V0v spinal interneurons. (**A**) 2D UMAP plot of 48 h post-mitotic V0v spinal interneuron single-cell RNA-seq atlas (2860 cells). Cells were obtained from 48 h embryos produced from an incross of *evx1*^*i232/*+^*;evx2*^*sa140/*+^ heterozygous parents homozygous for *Tg(evx1:EGFP)*^*SU2*^. Clusters are color-coded by cell identity: V0v WT Group 1 (light green), V0v WT Group 2 (dark green), V0v Mutant Group 1 (turquoise), V0v Mutant Group 2 (light blue), and V0v Mutant Group 3 (dark blue). For ease of cell type comparison, panel 7A has been reproduced from Fig. 6A. (**B**-**J**) 2D UMAP plots of differential gene expression between cell clusters. Black shows high levels of expression, light grey shows low levels of expression. All expression data have been normalized (see Methods). (**B**) *anos1a,* (**C**) *chrna2b,* (**D**) *fndc4b,* (**E**) *plpp4,* (**F**) *cnih3*, and (**G**) *drd2b* are all expressed in more cells in WT and Mutant Group 1 clusters than WT and Mutant Group 2 clusters. In contrast, (**H**) *esrrb,* (**I**) *scxa*, and (**J**) *svild* are all expressed in more cells in WT and Mutant Group 2 clusters than WT and Mutant Group 1 clusters

Our analyses of differential gene expression in the five distinct clusters of V0v cells, suggest that Mutant Group 1 is more similar to WT Group 1 than WT Group 2 and, conversely, Mutant Group 2 is more similar to WT Group 2 than WT Group 1 (Figs. 6 & 7, Tables 2 & 4). For example, there is a statistically significant increase in expression of *nefma* and *nefmb* in WT and Mutant Group 1 cells compared to WT and Mutant Group 2 cells (Fig. 6A, N–O; Table 2). Many other genes, including *neff1*, *anos1a, chrna2b, fndc4b, plpp4, cnih3* and *drd2b* are also expressed at higher levels in WT and Mutant Group 1 cells compared to WT and Mutant Group 2 cells (Fig. 6P, Fig. 7A-G, Tables 2 & 4, Supp. Tables 2 & 3). Similarly, several genes, including *esrrb, scxa* and *svild*, are expressed at statistically significant higher levels in WT and Mutant Group 2 cells, compared to WT and Mutant Group 1 cells (Fig. 7A, H-J, Tables 2 & 4, Supp. Tables 2 & 3). Taken together, these data suggest that there are two molecularly distinct subsets of WT V0v cells and a mutant version of each.

**Table 4:** Differential expression analysis between V0v Group 1 and Group 2 WT and *evx1;evx2* mutant spinal interneurons

Gene Symbol	Gene Name	Hurdle Model Fold-Change		ANOVA Fold-Change	
		**Mutant 1 vs Mutant 2**	**WT 1 vs WT 2**	**Mutant 1 vs Mutant 2**	**WT 1 vs WT 2**
*anos1a*	*anosmin 1a*	↑13.57***	↑7.77***	↑3.79***	↑17.10***
*chrna2b*	*cholinergic receptor, nicotinic, alpha 2b (neuronal)*	↑10.27***	↑5.75***	↑83.25***	↑45.56***
*fndc4b*	*fibronectin type III domain containing 4b*	↑7.96***	↑2.29***	↑33.76***	↑24.27***
*plpp4*	*phospholipid phosphatase 4*	N.C.	↑1.67***	↑54.02***	↑31.39***
*cnih3*	*cornichon family AMPA receptor auxiliary protein 3*	↑2.58***	↑1.56***	↑24.54***	↑19.16***
*drd2b*	*dopamine receptor D2b*	N.C.	↑1.41***	↑30.07***	↑19.48***
*esrrb*	*estrogen-related receptor beta*	↓5.49***	↓72.83***	↓16.07***	↓17.79***
*scxa*	*scleraxis bHLH transcription factor a*	↓1.92***	↓25.39***	↓3.54***	↓4.53***
*svild*	*supervillin d*	↓1.35***	↓1.62***	↓7.24***	↓6.94***

Gene-specific analyses of differential expression, created through Hurdle model (columns 3–4) and ANOVA (columns 5–6) statistical comparisons between distinct cell clusters in our 48 h *evx1*^*i232/*+^*;evx2*^*sa140/*+^ heterozygote incross single-cell atlas (see Fig. 7A and also Methods for experimental details and rationale for using both statistical methods). For these comparisons, the Hurdle model is probably the most statistically robust method as there are sufficient cell numbers in each group to effectively model the variance (see Methods for more information). We also provide the ANOVA data for completeness and comparison. Columns 1 and 2 show the gene symbol and the full gene name respectively. Columns 3–6 show fold-change values. ↑ = fold-change increase, ↓ = fold-change decrease in the antecedent (first) population compared to the consequent (second) population in each comparison. Statistically significant (*P* < 0.05) values are indicated in bold. *** *P* < 0.001*.* N.C. = Not Calculated. Differential expression cannot be calculated, usually because there is no expression in one population in the comparison. Mutant Group 1 is the antecedent population for columns 3 and 5 respectively. WT Group 1 is the antecedent population for columns 4 and 6 respectively. Mutant Group 2 is the consequent population for columns 3 and 5 respectively. WT Group 2 is the consequent population for columns 4 and 6 respectively. Additional data for each comparison are available in Supp. Data Tables 2 (Hurdle model data) and 3 (ANOVA data)

### Mutant group 3 cells express genes normally expressed in distinct populations of inhibitory spinal interneurons, or motoneurons

**Fig. 8:**
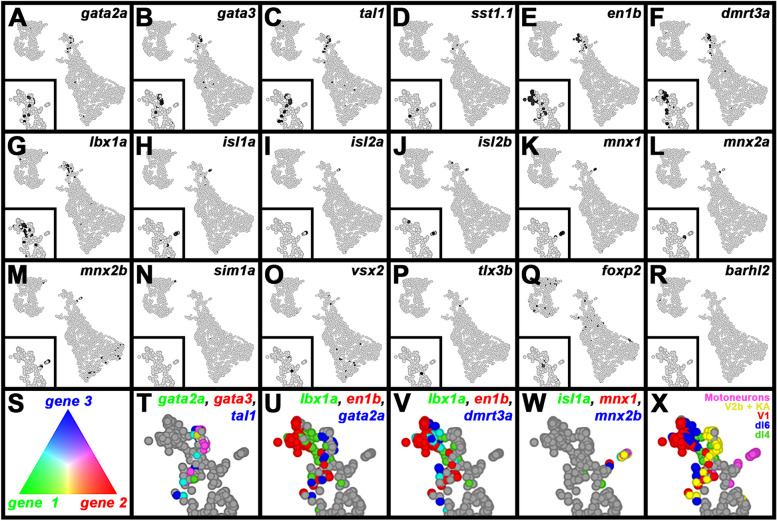
*evx1;evx2* Mutant Group 3 cells mis-express inhibitory spinal interneuron, or motoneuron genes. (**A**-**R**) 2D UMAP plots of differential gene expression between cell clusters in the 48 h post-mitotic V0v spinal interneuron single-cell RNA-seq atlas. For cell cluster identities, see Fig. 6A. Black shows high levels of expression, light grey shows low levels of expression. Inset panels in **A**-**R** show high-magnification views of Mutant Group 3 cells. For the number of cells expressing each gene see Table 3. (**T-X**) High magnification views of Mutant Group 3 cells showing three-way differential gene expression (**T-W**) or different cell fates (**X**). Panel (**S**) indicates the color-coding for panels (**T-W**). (**T-W**) Cells expressing only gene 1 are green. Cells expressing only gene 2 are red. Cells expressing only gene 3 are blue. Cells are yellow, pink, or turquoise if they co-express genes 1 and 2, genes 2 and 3, and genes 1 and 3 respectively. Cells expressing all three genes are white. All expression data have been normalized (see Methods). (**A-G**) Distinct subsets of Mutant Group 3 cells express markers of inhibitory spinal neurons, including (**A**) *gata2a,* (**B**) *gata3*, and (**C**) *tal1* (usually expressed by KA’, KA’’ and V2b inhibitory interneurons), (**D**) *sst1.1* (usually expressed by KA’ inhibitory interneurons), (**E**) *en1b* (usually expressed by V1 inhibitory interneurons), (**F**) *dmrt3a* (usually expressed by dI6 inhibitory interneurons), and (**G**) *lbx1a* (usually expressed by dI4 and dI6 inhibitory interneurons, although it is also expressed in dI5 excitatory interneurons). (**H-M**) A further subset of Mutant Group 3 cells co-express markers of acetylcholinergic motoneuron cells, including (**H**) *isl1a,* (**I**) *isl2a,* (**J**) *isl2b,* (**K**) *mnx1,* (**L**) *mnx2a*, and (**M**) *mnx2b*. (**N-R**) In contrast, Mutant Group 3 cells do not strongly express markers of other excitatory spinal neurons, such as (**N**) *sim1a* (usually expressed by V3 excitatory interneurons), (**O**) *vsx2* (usually expressed by V2a excitatory interneurons), (**P**) *tlx3b* (usually expressed by dI3 and dI5 excitatory interneurons), (**Q**) *foxp2* (usually expressed by dI2 excitatory interneurons, although it is also expressed in V1 inhibitory interneurons), and (**R**) *barhl2* (usually expressed by dI1 excitatory interneurons). (**T**) KA’, KA’’ and V2b genes, *gata2a, gata3* and *tal1* are co-expressed in a distinct subset of Mutant Group 3 cells. (**U-V**) Adjacent and to the left of this KA/V2b-like subset are two distinct subsets of cells expressing *lbx1a* (green cells in U and V) and/or *dmrt3a* (blue and turquoise cells in V), or *en1b* (red cells in U-V), which are expressed by dI4 (*lbx1a*), dI6 (*lbx1a* + *dmrt3a)* and V1 (*en1b*) interneurons respectively. (**W**) Adjacent and to the right of the KA/V2b-like subset of Mutant Group 3 cells shown in T, is a subset of cells co-expressing the motoneuron genes *isl1a, mnx1*, and *mnx2b*. (**X**) Sub-clusters are color-coded by cell identity assigned based on the differential expression profiles shown in A-M and T-W: Motoneurons (pink), V2b + KA neurons (yellow), V1 neurons (red), dI6 neurons (blue), and dI4 neurons (green)

Cells in Mutant Group 3 appear to represent the most severely affected *evx1/2* mutant cells, based on several key criteria. These cells have the lowest levels of *evx1* and *evx2* expression, almost none of them express the glutamatergic gene, *slc17a6a*, most of them express the inhibitory glycinergic gene, *slc6a5,* and some cells also express inhibitory GABAergic markers, including *slc6a1b* and *gad1b* (Fig. 6A-G, Table 2). To our surprise, differential expression analyses between cells in this cluster and all the other clusters, identified several transcription factor genes that are usually expressed by either distinct populations of inhibitory spinal cord interneurons, or motoneurons, upregulated in Mutant Group 3 cells (Fig. 8, Table 3, Table 5, Supp. Tables 2 & 3). For example, there was a statistically significant increase in the expression of *gata2a, gata3, tal1* and *sst1.1* (expressed by KA and V2b cells [37, 72, 74, 75]), *en1b* (expressed by V1 cells [6, 71]), *dmrt3a* (expressed by dI6 cells [56, 76, 77]), *lbx1a* (expressed by dI4 and dI6 cells, [4, 7, 78–81]) (Fig. 8A-G, Table 5) and *isl1a, isl2a, isl2b, mnx1, mnx2a* and *mnx2b* (expressed by motoneurons [82–85] (Fig. 8H-M, Table 5)). Interestingly, UMAP analysis suggests that these markers of different cell types are expressed by small distinct groups of Mutant Group 3 cells (Fig. 8 & Table 3). For example, the Mutant Group 3 cells that express the KA and V2b genes *gata2a*, *gata3, tal1,* or *sst1.1* overlap with each other (Fig. 8A-D), but these 42 cells (42/201 (20.90%), of which, 29/42 (69.05%) co-express two or more of these genes) are spatially distinct from those that express markers of motoneurons, V1 interneurons or more dorsal inhibitory interneurons (Fig. 8E-M & S-X). Similarly, the 20/201 (9.95%) Mutant Group 3 cells that express at least one of the motoneuron genes *isl1a, isl2a, isl2b, mnx1, mnx2a* and *mnx2b* do not co-express interneuron markers (Fig. 8A–M & W-X)*.* In addition, *en1b, dmrt3a* and *lbx1a* are each expressed by distinct subclusters of 57 (28.36%), 28 (13.93%) and 22 (10.95%) Mutant Group 3 cells respectively, that do not overlap with any of the cells expressing other markers of inhibitory interneuron fates (Fig. 8E-G, U-V & X, Table 3).

**Table 5:** Differential expression analysis of *evx1;evx2* Mutant Group 3 V0v spinal interneurons

Gene Symbol	Interneuron Marker	Interneuron Neurotransmitter Phenotype	Hurdle Model Statistical Data			ANOVA Statistical Data		
			**Least Squares Mean Reads**		**Fold-Change: Mutant 3 vs All Other Groups**	**Least Squares Mean Reads**		**Fold-Change: Mutant 3 vs All Other Groups**
			**Mutant Group 3**	**All Other Groups**		**Mutant Group 3**	**All Other Groups**	
*gata2a*	KA’, KA”, and V2b	Inhibitory	1.95	1.01	↑1.94***	70.63	1.40	↑50.50***
*gata3*	KA’, KA”, and V2b	Inhibitory	2.34	1.01	↑2.32***	154.55	1.80	↑85.98***
*tal1*	KA’, KA”, and V2b	Inhibitory	2.74	1.03	↑2.65***	138.08	2.68	↑51.44***
*sst1.1*	KA”	Inhibitory	N.C.	N.C.	N.C.	166.67	1.00	↑166.67***
*en1b*	V1	Inhibitory	8.36	1.01	↑8.26***	457.91	1.56	↑294.43***
*dmrt3a*	dI6	Inhibitory	2.77	1.02	↑2.71***	275.63	2.71	↑101.77***
*lbx1a*	dI4, dI5, and dI6	Inhibitory (dI4, dI6) Excitatory (dI5)	1.93	1.01	↑1.91***	59.87	1.72	↑34.78***
*islet1a*	Motoneurons	Acetylcholinergic	1.29	1.01	↑1.28***	21.25	1.69	↑12.58***
*islet2a*	Motoneurons	Acetylcholinergic	1.46	1.01	↑1.45***	54.58	1.56	↑35.06***
*islet2b*	Motoneurons	Acetylcholinergic	1.21	1.00	↑1.21***	18.15	1.32	↑13.73***
*mnx1*	Motoneurons	Acetylcholinergic	1.42	1.00	↑1.42***	36.21	1.16	↑31.19***
*mnx2a*	Motoneurons	Acetylcholinergic	N.C.	N.C.	N.C.	15.16	1.00	↑15.16***
*mnx2b*	Motoneurons	Acetylcholinergic	1.41	1.03	↑1.37***	25.61	3.66	↑6.99***
*sim1a*	V3	Excitatory	1.00	1.00	↓1.00	1.00	1.30	↓1.30
*vsx2*	V2a	Excitatory	1.12	1.02	↑1.10	15.06	3.26	↑4.62
*tlx3b*	dI3 and dI5	Excitatory	N.C	N.C	N.C	2.11	1.00	↑2.11**
*foxp2*	dI2	Excitatory	1.19	1.16	↑1.03	19.54	10.47	↑1.87
*barhl2*	dI1	Excitatory	N.C.	N.C.	N.C.	1.00	1.73	↓1.73

Gene-specific analyses of differential expression, created through Hurdle model (columns 4–6) and ANOVA (columns 7–9) statistical comparisons between distinct cell clusters in our 48 h *evx1*^*i232/*+^*;evx2*^*sa140/*+^ heterozygote incross single-cell atlas (see Fig. 6A and also Methods for experimental details and rationale for using both statistical methods). For these comparisons, the ANOVA data is probably the most robust, as the number of cells in each sub-cluster is relatively small (see Methods for more information). We also include the Hurdle model data for completeness. Column 1 shows the gene symbol. Column 2 indicates the spinal neuron types that normally express the gene. Column 3 indicates the neurotransmitter phenotype for these neurons. Columns 4 and 7 show least squares mean read counts for cells in Mutant Group 3, and columns 5 and 8 show least squares mean read counts for cells in all the other clusters combined, respectively. Columns 6 and 9 show fold-change values. ↑ = fold-change increase, ↓ = fold-change decrease in Mutant Group 3 (the antecedent (first) population) compared to all other populations combined (the consequent (second) population). Statistically significant (*P* < 0.05) values are indicated in bold. *** *P* < 0.001. ** P < 0.01. N.C. = Not Calculated. Hurdle model of differential expression analysis cannot be calculated, usually because expression was too low or not present in one of the groups being compared. Additional data for this comparison is available in Supp. Data Tables 2 (Hurdle model data) and 3 (ANOVA data)

In contrast, with the exception that there may be an occasional cell at the boundary between Mutant Group 3 and WT Group 1 that expresses *slc17a6a* and either *vsx2* or *tlx3b* (Fig. 6D & Fig. 8O-P), we do not observe upregulation of genes expressed by spinal excitatory interneuron populations in the UMAP analysis. With the exception of *tlx3b*, there is also no statistically significant change in the expression of genes expressed by spinal excitatory interneuron populations, in Mutant Group 3 cells compared to the other clusters (Table 5). Specifically, we examined *sim1a* (V3 interneurons [86, 87]), *vsx2* (V2a interneurons, [37, 88]), *tlx3b* (dI3 and dI5 interneurons [4, 11, 89]), *foxp2* (dI2 interneurons [90, 91]) and *barhl2* (dI1 interneurons [92]) expression (Fig. 8N-R, Table 5). Taken together, these data suggest that distinct subsets of Mutant Group 3 cells have started to express markers of distinct inhibitory spinal neurons (V1, V2b, KA, dI4 or dI6 neurons), or cholinergic motoneurons.

### Additional transcription factor genes are either downregulated or upregulated in V0v interneurons that lack Evx1 and/or Evx2 function

**Fig. 9:**
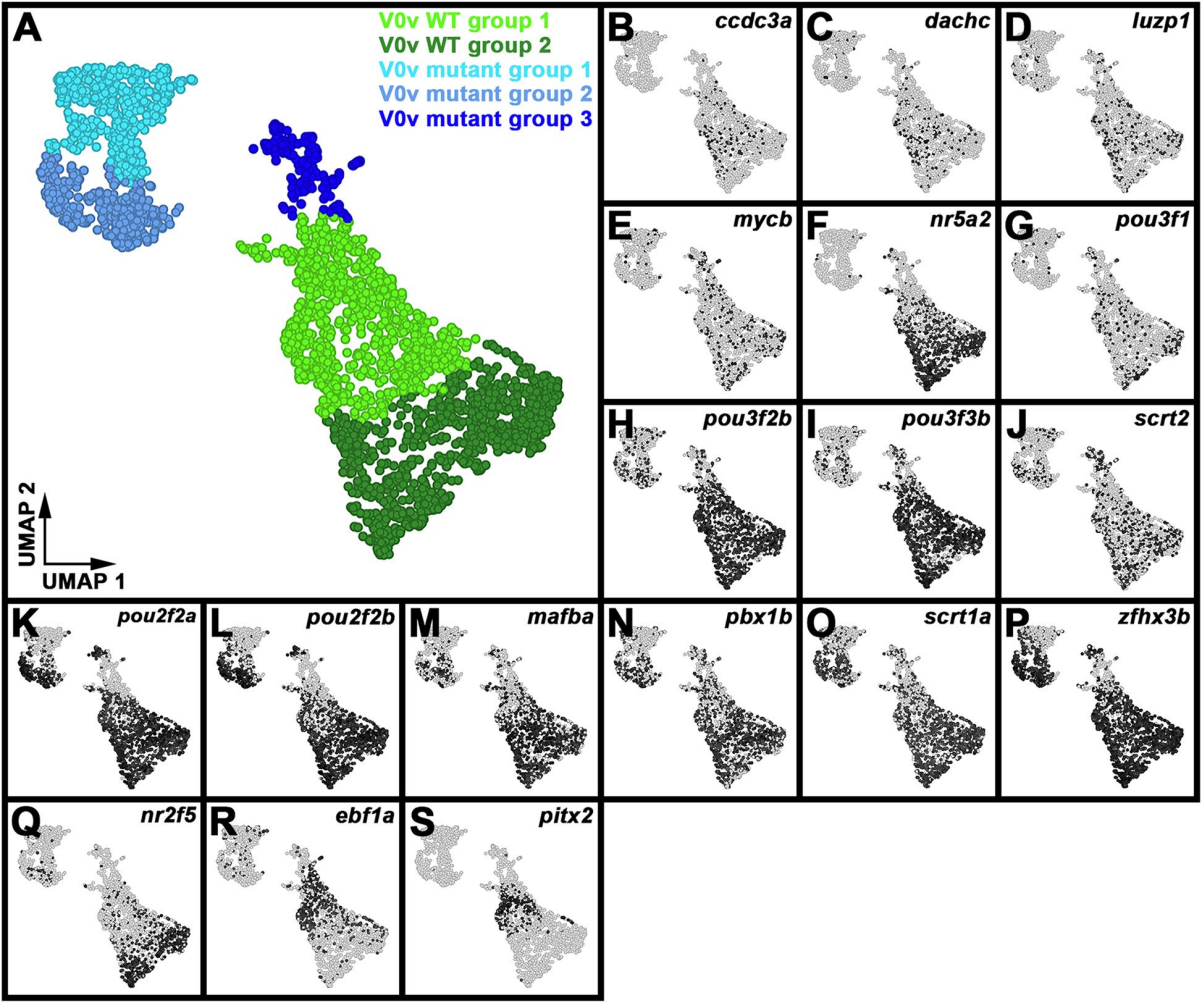
Genes downregulated in *evx1;evx2* Mutant Group 1 and 2 V0v spinal interneurons. (**A**) 2D UMAP plot of the 48 h post-mitotic V0v spinal interneuron single-cell RNA-seq atlas (2860 cells). Cells were obtained from 48 h embryos produced from an incross of *evx1*^*i232/*+^*;evx2*^*sa140/*+^ heterozygous parents homozygous for *Tg(evx1:EGFP)*^*SU2*^. Clusters are color-coded by cell identity: V0v WT Group 1 (light green), V0v WT Group 2 (dark green), V0v Mutant Group 1 (turquoise), V0v Mutant Group 2 (light blue), and V0v Mutant Group 3 (dark blue). For ease of cell type comparison, panel 9A has been reproduced from Fig. 6A. (**B**-**S**) 2D UMAP plots of differential gene expression between cell clusters. Black shows high levels of expression, light grey shows low levels of expression. All expression data have been normalized (see Methods). (**B**) *ccdc3a,* (**C**) *dachc,* (**D**) *luzp1,* (**E**) *mycb,* (**F**) *nr5a2,* (**G**) *pou3f1,* (**H**) *pou3f2b,* (**I**) *pou3f3b,* (**J**) *scrt2*, (**K**) *pou2f2a,* (**L**) *pou2f2b,* (**M**) *mafba,* (**N**) *pbx1b,* (**O**) *scrt1a,* (**P**) *zfhx3b,* (**Q**) *nr2f5*, (**R**) *ebf1a* and (**S**) *pitx2*

In addition to the genes that we had already identified, our scRNA-seq data also identified additional transcription factor genes that may be part of V0v GRNs, as they are expressed in at least some V0v WT cells and are downregulated in V0v mutant cells (Fig. 9, Table 6). *ccdc3a, dachc, luzp1, mycb, nr5a2, pou3f1, pou3f2b, pou3f3b* and *scrt2* are expressed in both WT clusters and this expression is reduced in all three Mutant Groups (Fig. 9B-J and Table 6A, with the exceptions that the reduction of expression in the Mutant Group 3 cluster is not statistically significant using the Hurdle model for *dachc* and *luzp1*, and expression of *mycb*, and possibly *scrt2*, is upregulated in the Mutant Group 3 cluster compared to WT cells). There is also a statistically significant reduction of *pou2f2a, pou2f2b, mafba, pbx1b, scrt1a* and *zfhx3b* expression in all three mutant groups, according to the Hurdle model. These genes are all expressed by many cells in WT and Mutant Group 2, as well as a significant number of cells in the WT Group 1 cluster and some cells in the other two mutant groups (Fig. 9K-P, Table 6A). Interestingly, *pou2f2a, pou2f2b* and *zfhx3b* appear to be co-expressed in a subset of Mutant Group 3 cells at the top of the cluster, which also express markers of V1 or dI6 cells (Fig. 8V, X & Fig. 9K-L & P). *nr2f5* has a similar expression pattern to these six genes, except that far fewer cells express this gene in the WT or Mutant Group 1 clusters (Fig. 9Q). In contrast, *ebf1a* and *pitx2* are mainly expressed by cells in the WT Group 1 cluster and there are very few cells that express either of these genes in either the WT Group 2 cluster or any of the mutant clusters (Fig. 9R-S, Table 6).

**Table 6:** Differential expression analysis of genes downregulated in *evx1;evx2* Mutant Group 1 and 2 V0v spinal interneurons

**A**							
**Gene Symbol**	**Hurdle Model Fold-Change**						
	**Mutant 1 + 2 vs WT 1 + 2**	**Mutant 3 vs Mutant 1 + 2**	**Mutant 1 vs WT 1**	**Mutant 2 vs WT 2**	**Mutant 3 vs WT 1 + 2**	**WT 1 vs WT 2**	**Mutant 1 vs Mutant 2**
*ccdc3a*	↓1.71***	↑1.01	↓1.74***	↓1.64***	↓1.68**	↑1.16***	↑1.09
*dachc*	↓1.73***	↑1.15	↓1.83***	↓1.63***	↓1.50	↑1.27***	↑1.13**
*luzp1*	↓1.70***	↑1.60***	↓1.71***	↓1.63***	↓1.06	↓1.25***	↓1.31***
*mycb*	↓1.97***	↑3.76***	↓1.40*	↓2.85***	↑1.92***	↓1.70***	↑1.20
*nr5a2*	↓37.70***	↑1.88***	↓7.47***	↓151.81***	↓19.83***	↓19.58***	↑1.04
*pou3f1*	↓2.43***	↑2.09***	↓1.80***	↓3.47***	↓1.16*	↓1.83***	↑1.05
*pou3f2b*	↓25.86***	↑1.12	↓32.33***	↓17.28***	↓22.90***	↓1.61***	↓3.01***
*pou3f3b*	↓47.31***	↑1.86*	↓45.00***	↓47.63***	↓25.32***	↓1.53**	↓1.44***
*scrt2*	↓2.41***	↑3.38***	↓2.58***	↓1.80***	↑1.40	↓2.34***	↓3.36***
*pou2f2a*	↓8.56***	↓2.15*	↓28.23***	↓1.17***	↓18.39***	↓6.83***	↓163.63***
*pou2f2b*	↓6.78***	↓4.47***	↓13.05***	↓1.50***	↓30.31***	↓12.38***	↓107.28***
*mafba*	↓15.72***	↓1.21	↓16.24***	↓9.56***	↓18.90***	↓3.36***	↓5.69***
*pbx1b*	↓4.70***	↓1.20	↓9.17***	↓1.68***	↓5.65***	↓2.09***	↓11.35***
*scrt1a*	↓3.86***	↓4.17***	↓4.13***	↓2.60***	↓16.10***	↓6.70***	↓10.61***
*zfhx3b*	↓5.43***	↓3.78***	↓17.11***	↓1.78***	↓20.52***	↓3.89***	↓37.40***
*nr2f5*	↓6.23***	↓1.19	↓2.07***	↓20.77***	↓7.42***	↓24.31***	↓2.42***
*ebf1a*	↓2.66***	↑2.76***	↓12.54***	↓1.13***	↑1.04**	↑14.27***	↑1.28
*pitx2*	↓1.64***	↑1.01	↓10.67***	↓1.06*	↓1.63*	↑9.95***	↓1.02
**B**							
**Gene Symbol**	**ANOVA Fold-Change**						
	**Mutant 1 + 2 vs WT 1 + 2**	**Mutant 3 vs Mutant 1 + 2**	**Mutant 1 vs WT 1**	**Mutant 2 vs WT 2**	**Mutant 3 vs WT 1 + 2**	**WT 1 vs WT 2**	**Mutant 1 vs Mutant 2**
*ccdc3a*	↓6.93***	↓1.03	↓5.59***	↓11.62***	↓7.16***	↑1.69	↑3.50
*dachc*	↓4.18***	↓1.61	↓4.44***	↓3.77***	↓2.59*	↑1.88*	↑1.60
*luzp1*	↓1.89***	↑1.62**	↓3.04***	↓1.40***	↓1.17	↓1.06	↓2.31
*mycb*	↓2.32***	↑4.63***	↓1.55*	↓3.54***	↑1.99**	↓1.44***	↑1.58
*nr5a2*	↓75.12***	↑15.38***	↓32.40***	↓129.88***	↓4.88***	↓3.13***	↑1.28
*pou3f1*	↓5.40***	↑5.97***	↓3.82***	↓7.04***	↑1.10	↓1.78***	↑1.04
*pou3f2b*	↓3.65***	↓1.08	↓6.93***	↓2.49***	↓3.94***	↓1.02***	↓2.83***
*pou3f3b*	↓6.54***	↑1.40*	↓9.14***	↓5.26***	↓4.69***	↓1.16**	↓2.02
*scrt2*	↓1.34***	↑1.62***	↓2.79***	↑1.02**	↑1.21	↓1.41***	↓4.01***
*pou2f2a*	↓1.10***	↓1.66***	↓7.34***	↑1.33	↓1.83***	↓1.84***	↓17.92***
*pou2f2b*	↓1.17***	↓2.25***	↓5.14***	↑1.16**	↓2.64***	↓2.19***	↓13.01***
*mafba*	↓3.00***	↓1.34	↓7.93***	↓2.00***	↓4.04***	↓1.24***	↓4.92***
*pbx1b*	↓1.52***	↓1.57	↓3.91***	↓1.00*	↓2.38***	↓1.18***	↓4.60***
*scrt1a*	↓1.27***	↓3.09***	↓2.15***	↓1.04***	↓3.93***	↓1.86***	↓3.86***
*zfhx3b*	↓1.49***	↓1.58***	↓3.49***	↓1.09***	↓2.36***	↓1.54***	↓4.94***
*nr2f5*	↓4.09***	↓3.27*	↓6.08***	↓3.87***	↓13.34***	↓6.01***	↓9.42***
*ebf1a*	↓4.27***	↑4.38***	↓6.44***	↑1.03	↑1.03	↑10.15***	↑1.52
*pitx2*	↓175.87***	↑1.16	↓416.72***	↓7.19	↓152.16***	↑40.61***	↓1.43

Gene-specific analyses of differential expression, created through (**A**) Hurdle model and (**B**) ANOVA statistical comparisons between distinct cell clusters in our 48 h *evx1*^*i232/*+^*;evx2*^*sa140/*+^ heterozygote incross single-cell atlas (see Fig. 9A and also Methods for experimental details and rationale for using both statistical methods). For these comparisons, the Hurdle model is probably the most statistically robust method as there are sufficient cell numbers in each group to effectively model the variance (see Methods for more information). We also provide the ANOVA data for completeness and comparison. Column 1 shows the gene symbol. Columns 2–8 show fold-change values. ↑ = fold-change increase, ↓ = fold-change decrease in the antecedent (first) population compared to the consequent (second) population in each comparison. Statistically significant (*P* < 0.05) values are indicated in bold. *** *P* < 0.001, ** *P* < 0.01, * *P* < 0.05*.* Mutant Groups 1 + 2 combined, Mutant Group 3, Mutant Group 1, Mutant Group 2, Mutant Group 3, WT Group 1, and Mutant Group 1 are the antecedent populations for columns 2, 3, 4, 5, 6, 7 and 8 respectively. WT Groups 1 + 2 combined, Mutant Groups 1 + 2 combined, WT Group 1, WT Group 2, WT Groups 1 + 2 combined, WT Group 2 and Mutant Group 2 are the consequent populations for columns 2, 3, 4, 5, 6, 7 and 8 respectively. Additional data for each comparison are available in Supp. Data Tables 2 (Hurdle model data) and 3 (ANOVA data)

**Fig. 10:**
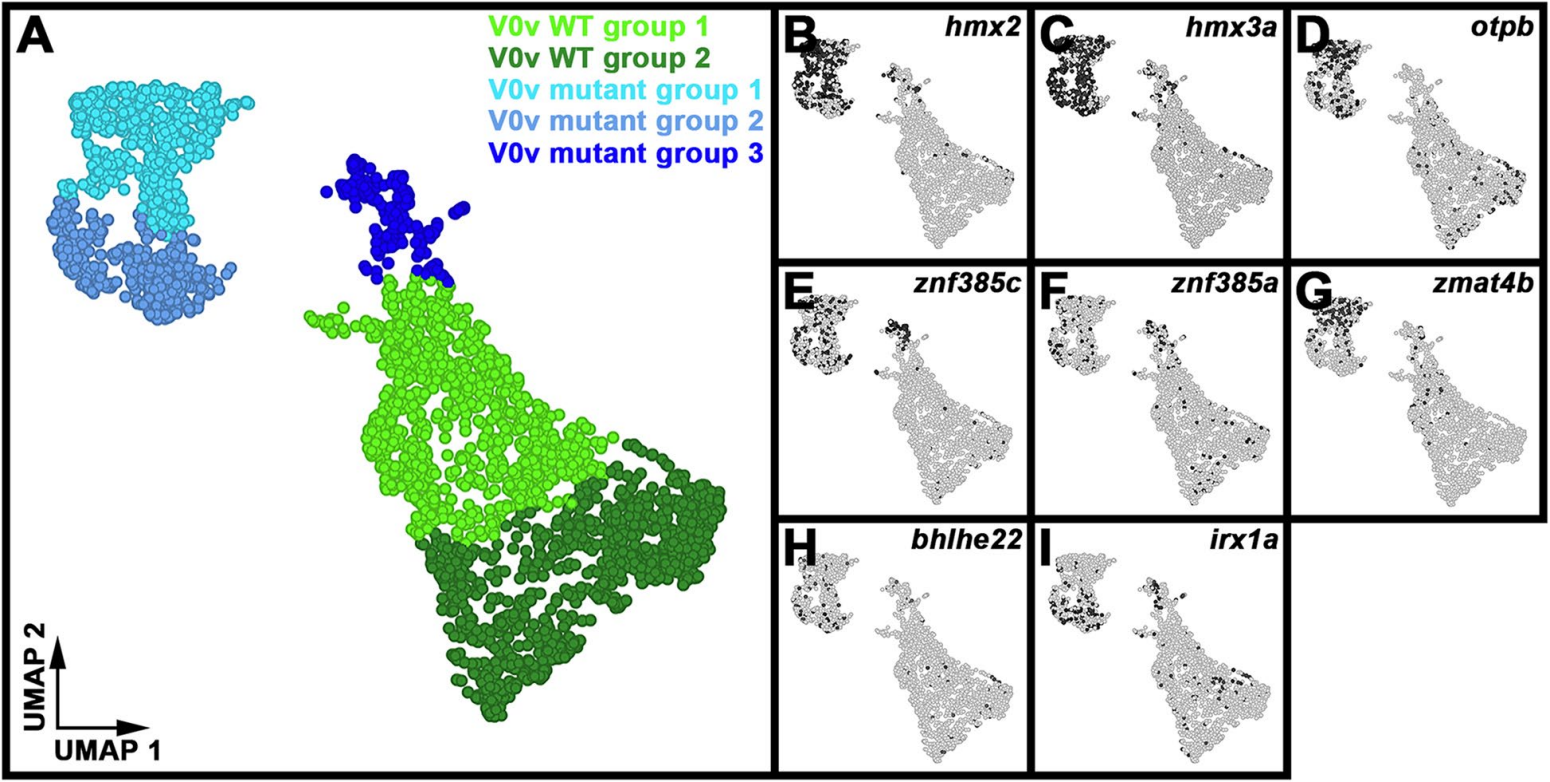
Genes upregulated in *evx1;evx2* Mutant Group 1 and 2 V0v spinal interneurons. (**A**) 2D UMAP plot of the 48 h post-mitotic V0v spinal interneuron single-cell RNA-seq atlas (2860 cells). Cells were obtained from 48 h embryos produced from an incross of *evx1*^*i232/*+^*;evx2*^*sa140/*+^ heterozygous parents homozygous for *Tg(evx1:EGFP)*^*SU2*^. Clusters are color-coded by cell identity: V0v WT Group 1 (light green), V0v WT Group 2 (dark green), V0v Mutant Group 1 (turquoise), V0v Mutant Group 2 (light blue), and V0v Mutant Group 3 (dark blue). For ease of cell type comparison, panel 10A has been reproduced from Fig. 6A. (**B-I**) 2D UMAP plots of differential gene expression between cell clusters. Black shows high levels of expression, light grey shows low levels of expression. All expression data have been normalized (see Methods). (**B**) *hmx2,* (**C**) *hmx3a,* (**D**) *otpb*, (**E**) *znf385c*, (**F**) *znf385a,* (**G**) *zmat4b*, (**H**) *bhlhe22*, and (**I**) *irx1a*

We also identified some transcription factor genes that are upregulated in V0v mutant cells compared to WT cells (Fig. 10, Table 7). As with the downregulated genes, these have a few different patterns of expression. For example, *hmx2, hmx3a*, *otpb* and *znf385c* are all highly expressed in Mutant Group 1 and 2 cells, but only a few cells express these genes in either of the WT clusters or, in the case of *hmx2, hmx3a* and *otpb,* in the Mutant Group 3 cluster. *znf385c* is expressed in more Mutant Group 3 cells than the other three genes and it is expressed at statistically significant higher levels in this cluster than in the other two mutant clusters (Fig. 10B-E, Table 7). *znf385a* has a similar pattern of expression to these four genes, but it is expressed in fewer Mutant Group 1 and 2 cells, and like *znf385c,* it is expressed at highest levels in the Mutant Group 3 cluster (Fig. 10F, Table 7). In contrast, *zmat4b* is predominantly expressed by cells in Mutant Group 1, although there is still a statistically significant increase in expression of this gene in the Mutant Group 2 cluster compared to the WT Group 2 cluster and in the Mutant Group 3 cluster compared to WT cells (Fig. 10G, Table 7). In contrast, *bhlhe22* and *irx1a* are predominantly expressed by cells in the Mutant Group 2 cluster, although there is still a statistically significant increase in expression of *bhlhe22* in the Mutant Group 1 cluster compared to WT Group 1, and of *bhlhe22* and *irx1a* in the Mutant Group 3 cluster compared to WT cells (Fig. 10H-I, Table 7).

**Table 7:** Differential expression analysis of genes upregulated in *evx1;evx2* Mutant Group 1 and 2 V0v spinal interneurons

**A**							
**Gene Symbol**	**Hurdle Model Fold-Change**						
	**Mutant 1 + 2 vs WT 1 + 2**	**Mutant 3 vs Mutant 1 + 2**	**Mutant 1 vs WT 1**	**Mutant 2 vs WT 2**	**Mutant 3 vs WT 1 + 2**	**WT 1 vs WT 2**	**Mutant 1 vs Mutant 2**
*hmx2*	↑29.04***	↓8.42***	↑52.12***	↑15.65***	↑3.42***	↑1.02	↑3.41***
*hmx3a*	↑66.64***	↓19.79***	↑44.99***	↑97.79***	↑3.34***	↑1.07	↓2.03***
*otpb*	↑7.22***	↓9.21***	↑15.17***	↑3.12***	↓1.28	↓1.65***	↑2.94***
*znf385c*	↑5.17***	↑2.33**	↑4.90***	↑5.35***	↑11.97***	↓1.10**	↓1.20***
*znf385a*	↑1.65***	↑1.61**	↑1.77***	↑1.49***	↑2.64***	↓1.16***	↑1.02***
*zmat4b*	↑2.88***	↓1.66***	↑9.09***	↑1.51***	↑1.72***	↑1.23***	↑7.36***
*bhlhe22*	↑1.84***	↓1.44**	↑1.51***	↑2.44***	↑1.28*	↓1.09	↓1.77***
*irx1a*	↑1.82***	↑1.01	↑1.07	↑5.22***	↑1.83***	↑1.04	↓4.68***
**B**							
**Gene Symbol**	**ANOVA Fold-Change**						
	**Mutant 1 + 2 vs WT 1 + 2**	**Mutant 3 vs Mutant 1 + 2**	**Mutant 1 vs WT 1**	**Mutant 2 vs WT 2**	**Mutant 3 vs WT 1 + 2**	**WT 1 vs WT 2**	**Mutant 1 vs Mutant 2**
*hmx2*	↑58.60***	↓3.14***	↑48.04***	↑83.14***	↑18.66***	↑2.32	↑1.34***
*hmx3a*	↑43.78***	↓4.66***	↑23.86***	↑87.66***	↑9.39***	↑2.20	↓1.67**
*otpb*	↑8.45***	↓42.06***	↑28.32***	↑4.13***	↓4.98***	↓4.60***	↑1.49***
*znf385c*	↑20.81***	↑1.56**	↑24.82***	↑18.68***	↑32.48***	↓1.89*	↓1.42
*znf385a*	↑3.83***	↑1.52*	↑4.26***	↑3.55***	↑5.82***	↓1.50*	↓1.25
*zmat4b*	↑11.70***	↓1.42***	↑11.26***	↑15.31***	↑8.21***	↑8.24***	↑6.07***
*bhlhe22*	↑14.58***	↓6.26	↑4.51***	↑21.00***	↑2.33**	↓1.57	↓7.31**
*irx1a*	↑6.85***	↓1.70	↑1.30	↑13.48***	↑4.02***	↑1.19	↓8.67***

Gene-specific analyses of differential expression, created through (**A**) Hurdle model and (**B**) ANOVA statistical comparisons between distinct cell clusters in our 48 h *evx1*^*i232/*+^*;evx2*^*sa140/*+^ heterozygote incross single-cell atlas (see Fig. 10A and also Methods for experimental details and rationale for using both statistical methods). For these comparisons, the Hurdle model is probably the most statistically robust method as there are sufficient cell numbers in each group to effectively model the variance (see Methods for more information). We also provide the ANOVA data for completeness and comparison. Column 1 shows the gene symbol. Columns 2–8 show fold-change values. ↑ = fold-change increase, ↓ = fold-change decrease in the antecedent (first) population compared to the consequent (second) population in each comparison. Statistically significant (*P* < 0.05) values are indicated in bold. *** *P* < 0.001, ** *P* < 0.01, * *P* < 0.05. Mutant Groups 1 + 2 combined, Mutant Group 3, Mutant Group 1, Mutant Group 2, Mutant Group 3, WT Group 1, and Mutant Group 1 are the antecedent populations for columns 2, 3, 4, 5, 6, 7 and 8 respectively. WT Groups 1 + 2 combined, Mutant Groups 1 + 2 combined, WT Group 1, WT Group 2, WT Groups 1 + 2 combined, WT Group 2 and Mutant Group 2 are the consequent populations for columns 2, 3, 4, 5, 6, 7 and 8 respectively. Additional data for each comparison are available in Supp. Data Tables 2 (Hurdle model data) and 3 (ANOVA data)

Taken together, these data identify multiple potential additional members of the GRNs downstream of Evx1 and Evx2 in V0v spinal interneurons. In future experiments it would be interesting to test whether any of the transcriptional regulators that are downregulated in V0v interneurons are required to specify the excitatory (glutamatergic) phenotype of V0v cells, and/or whether any of the upregulated genes are required to specify inhibitory fates, or repress excitatory fates, in mutant V0v cells.

### V0v interneurons ectopically express *hmx3a* in *evx1;evx2* mutants

Two of the genes that were upregulated in the majority of V0v Mutant Group 1 and Mutant Group 2 cells were *hmx2* and *hmx3a* (Fig. 10B-C, Table 7). We have previously shown that Hmx2 and Hmx3a are co-expressed in dI2 and V1 spinal interneurons, and that Hmx3a is required for the excitatory fates of dI2 interneurons. (Hmx2 also has a role in this process, but it is much more subtle than that of Hmx3a [12]). In the absence of *hmx3a* function, many dI2 interneurons change their neurotransmitter fates from glutamatergic (excitatory) to GABAergic (inhibitory) [12]. Therefore, we were surprised to discover that *hmx3a* is upregulated in mutant V0v cells that have changed their neurotransmitter phenotype from excitatory to inhibitory.

**Fig. 11:**
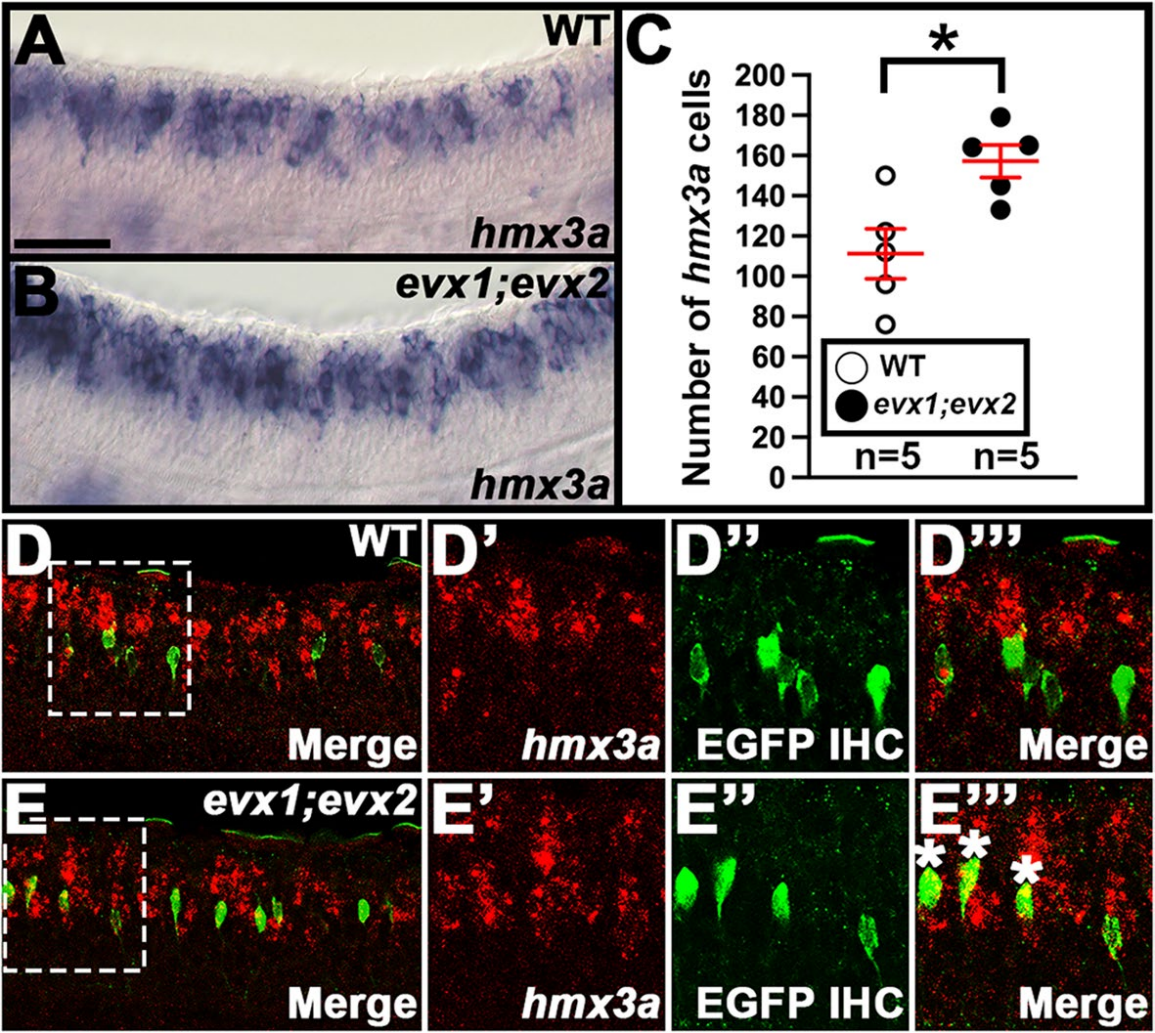
*hmx3a* expression is upregulated in a subset of V0v spinal interneurons in *evx1;evx2* double mutant embryos. (**A**,** B**,** D-D’’’**,** E-E’’’**) Lateral views of (**A**,** D-D’’’**) WT and (**B**,** E-E’’’**) *evx1*^*i232;i232*^*;evx2*^*sa140;sa140*^ double mutant embryos (labeled *evx1;evx2*) at 30 h. Rostral, left. Dorsal, up. (**C**) Number of cells expressing *hmx3a* in a precisely-defined spinal cord region adjacent to somites 6–10 at 30 h. Data are depicted as an individual value plot and *n-*values are indicated below. The wider red horizontal bar depicts the mean number of cells, and the red vertical bar depicts the S.E.M. (values are provided in Table 1). All counts are an average of five embryos. Statistically significant comparison is indicated with brackets and asterisks. * *P* < 0.05. White circles indicate WT data and black circles indicate *evx1;evx2* double mutant data. All data were first analyzed for normality using the Shapiro–Wilk test. Both data sets in C are normally distributed and so the F-test for equal variances was performed, followed by a type 2 Student’s *t*-test (for equal variances). *P*-values are provided in Table 1. (**C**) There is a statistically significant increase in the number of spinal interneurons expressing *hmx3a* in *evx1;evx2* double mutant embryos. (**D’**,** E’**) in situ hybridization for *hmx3a* is shown in red. (**D’’**,** E’’**) Immunohistochemistry for *Tg(evx1:EGFP)*^*SU2*^*,* which exclusively labels V0v interneurons in the spinal cord [14], is shown in green. (**D**,** D’’’**,** E**,** E’’’**) Merged images. (**D**,** E**) Maximum intensity projection images. (**D’-D’’’**,** E’-E’’’**) High-magnification single confocal planes of the regions indicated by white dotted boxes in D and E. (**E’’’**) A subset of ventral *hmx3a*-expressing cells in *evx1;evx2* double mutant embryos co-expresses *Tg(evx1:EGFP)*^*SU2*^ (white asterisks in **E’’’**), whereas there is no co-expression in the WT embryos (**D’’’**). Scale bar: (**A**,** B**,** D**, **E**) 50 µm, (**D’-D’’’**,** E’-E’’’**) 35 µm

**Fig. 12:**
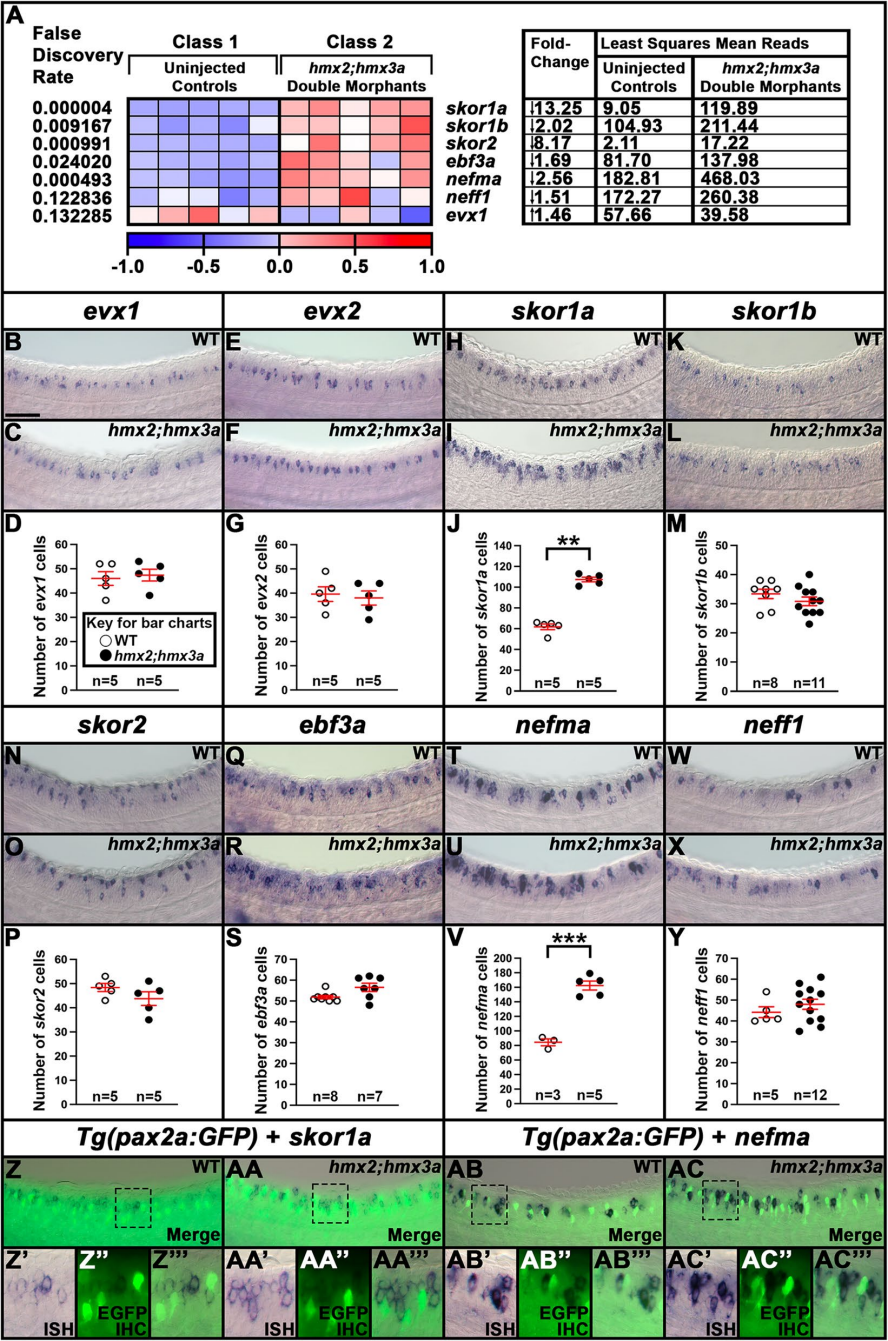
A subset of V0v spinal interneuron genes are upregulated in *hmx2;hmx3a* deletion mutant embryos. (**A**) Heatmap analysis of gene-expression profiling of 27 h *Tg(hmx CNEIII:cos:Gal4-VP16,UAS:EGFP)*^*SU41*^-expressing V1 and dI2 spinal cord interneurons. A two-class gene-specific analysis of differential expression was performed on different FAC-sorted populations of cells. Class 1: EGFP-positive cells from uninjected control embryos. Class 2: EGFP-positive cells from *hmx2;hmx3a* double knockdown (DKD) morpholino injected (morphant) embryos. Each column is a different biological replicate. Rows show relative expression levels for a subset of V0v candidate genes, shown as normalized data transformed to a mean of 0, with standard deviation of + 1 (highly expressed, red) or -1 (weakly/not expressed, blue) sigma units. Adjusted *P-*values corrected for multiple testing (false discovery rate values) are shown on the left-hand side. Column 1 of right-hand table indicates fold-change reduction (↓) in uninjected controls compared to *hmx2;hmx3a* DKD morphant embryos. Columns 2 and 3 of right-hand table show least squares mean read counts for uninjected controls and *hmx2;hmx3a* DKD morphant embryos respectively. *evx2* expression was not detected in either WT or morphant cells in this experiment. (**B**,** C**,** E**,** F**,** H**,** I**,** K**,** L**,** N**,** O**,** Q**,** R**,** T**,** U**,** W**,** X**,** Z-AC’’’**) Lateral views of (**B**,** E**,** H**,** K**,** N**,** Q**,** T**,** W**,** Z-Z’’’**,** AB-AB’’’**) homozygous WT and (**C**,** F**,** I**,** L**,** O**,** R**,** U**,** X**,** AA-AA’’’**,** AC-AC’’’**) homozygous *hmx2;hmx3a*^*SU44;SU44*^ deletion mutant embryos at 27 h. Rostral, left. Dorsal, up. (**D**,** G**,** J**,** M**,** P**,** S**,** V**,** Y**) Number of cells expressing (**D**) *evx1*, (**G**) *evx2*, (**J**) *skor1a,* (**M**) *skor1b*, (**P**) *skor2*, (**S**) *ebf3a,* (**V**) *nefma* and (**Y**) *neff1* in a precisely-defined spinal cord region adjacent to somites 6–10 at 27 h. Data are depicted as individual value plots with *n-*values provided below. For each plot, the wider red horizontal bar depicts the mean number of cells, and the red vertical bar depicts the S.E.M. (values are provided in Table 1). All counts are an average of at least three embryos. Statistically significant comparisons are indicated with brackets and asterisks. *** *P* < 0.001. ** *P* < 0.01. White circles indicate WT and black circles indicate data from homozygous *hmx2; hmx3a*^*SU44;SU44*^ mutants. All data were first analyzed for normality using Shapiro–Wilk test. Data sets in J and S are not normally distributed and Wilcoxon-Mann–Whitney tests were performed. Data sets in **D**,** G**,** M**,** P**,** V** and **Y** are normally distributed and so an F-test for equal variances was performed, followed by a type 2 Student’s *t*-test (for equal variances). *P*-values are provided in Table 1. (**J**, **V**) There is a statistically significant increase in the number of spinal interneurons expressing *skor1a* and *nefma*, but not (**D**,** G**, **M**,** P**,** S**,** Y**) *evx1, evx2, skor1b, skor2, ebf3a,* or *neff1* in homozygous *hmx2; hmx3a*^*SU44;SU44*^ mutant embryos. in situ hybridization for (**Z’**,** AA’**) *skor1a* and (**AB’**,** AC’**) *nefma* genes is shown in dark blue. (**Z’’**,** AA’’**,** AB’’**, **AC’’**) Immunohistochemistry for *Tg(pax2a:GFP),* which specifically labels V1 interneurons in the spinal cord [6], is shown in green. (**Z**,** Z’’’**,** AA**,** AA’’’**,** AB**,** AB’’’**,** AC**,** AC’’’**) Merged images. (**Z**,** AA**,** AB**,** AC**) Maximum intensity projection images. (**Z’-Z’’’**,** AA’-AA’’’**,** AB’-AB’’’**,** AC’-AC’’’**) High-magnification single confocal planes of the regions indicated by black dotted boxes in **Z**,** AA**,** AB**, and **AC**. (**AA’’’**,** AC’’’**) The increased numbers of cells expressing (**AA’’’’**) *skor1a* or (**AC’’’**) *nefma* in (**AA’’’**,** AC’’’**) homozygous *hmx2;hmx3a*^*SU44;SU44*^ mutant embryos do not co-express *Tg(pax2a:GFP)*, suggesting that the cells that have upregulated *skor1a* and *nefma* expression in these mutants are not V1 spinal interneurons. (**B**,** C**) *evx1*, (**E**, **F**) *evx2* and (**Q**,** R**) *ebf3a *in situ hybridization experiments were performed with the molecular crowding reagent Dextran Sulfate. This was omitted for (**H**, **I**) *skor1a*, (**K**, **L**) *skor1b*, (**N**,** O**) *skor2*, (**T**,** U**) *nefma* and (**W**,** X**) *neff1 *in situ hybridization experiments. Scale bar: (**B**,** C**,** E**, **F**,** H**,** I**,** K**,** L**,** N**,** O**,** Q**,** R**,** T**,** U**,** W**,** X**,** Z**,** AA**,** AB**,** AC**) 50 µm, (**Z’-Z’’’**,** AA’-AA’’’**,** AB’-AB’’’**,** AC’-AC’’’**) 20 µm

To further confirm this intriguing result, and examine whether it is also the case at earlier developmental stages, we analyzed *hmx3a* expression in 27 h *evx1;evx2* double mutants. We found that there is a statistically significant increase in the number of *hmx3a*-expressing cells in the double mutants, compared to WT siblings (Fig. 11A-C, Table 1). Double-labelling experiments with *Tg(evx1:EGFP)*^*SU2*^ confirmed that while V0v interneurons do not express *hmx3a* in WT embryos, many of them turn on *hmx3a* expression in *evx1;evx2* double mutants (Fig. 11D-E’’’), suggesting that Evx1/2 normally repress *hmx3a* expression in V0v interneurons. To assess whether Hmx3a might reciprocally repress *evx1* and *evx2* expression in V1 and dI2 spinal interneurons, we examined expression of *evx1* and *evx2* in a deletion mutant that lacks both *hmx2* and *hmx3a*. However, we found no change in the number of spinal cord cells expressing either *evx1* or *evx2* in *hmx2;hmx3a* deletion mutants compared to WT siblings (Fig. 12B-G, Table 1).

While we were expression-profiling V0v cells, we were also, for a different project, attempting to identify transcription factor genes that might act downstream of *hmx3a* in dI2 and/or V1 interneurons. We were doing this by comparing bulk RNA-Seq data from 27 h FAC-Sorted dI2 and V1 spinal interneurons isolated from *hmx2;hmx3a* double morphant and uninjected WT siblings embryos expressing *Tg(hmx CNEIII:cfos:Gal4-VP16,UAS:EGFP)*^*SU41*^. (The *Tg(hmx CNEIII:cfos:Gal4-VP16,UAS:EGFP)*^*SU41*^ transgenic line recapitulates endogenous *hmx3a* expression in the zebrafish spinal cord (Supp. Figure 4)). To our surprise, we found that expression of the V0v gene, *skor1a*, was upregulated more than 13-fold in dI2 and/or V1 interneurons from *hmx2;hmx3a* double morphants compared to uninjected controls (Fig. 12A, Supp. Table 4). This suggests that Hmx2 and/or Hmx3a normally repress *skor1a* expression in dI2 and/or V1 cells. Intrigued by this result, we examined whether expression of any of the other five genes downregulated in *evx1*;*evx2* mutants at 30 h, *skor1b, skor2, ebf3a*, *nefma* or *neff1* (Fig. 4D-L, Fig. 5A-C, G-I, Table 1) was upregulated in *hmx2;hmx3a* double morphants. The bulk RNA-seq data suggested that *skor1b* and *nefma* expression might also be upregulated (Fig. 12A, Supp. Table 4). Too few reads were reliably detected to assess whether *skor2* is also upregulated and the fold-change for both *ebf3a* and *neff1* was less than two, suggesting that any differences might be due to noise in the experiment.

As some of these results were inconclusive, and also because our previous analyses identified some differences between the phenotypes of *hmx2;hmx3a* double morphants and homozygous *hmx2;hmx3a* deletion mutants [12], we decided to analyze the spinal cord expression of *skor1a*, *skor1b*, *skor2*, *ebf3a, nefma* and *neff1* in these mutants. We found that there was a statistically significant increase in the number of spinal cord cells expressing either *skor1a* or *nefma* in *hmx2;hmx3a* deletion mutants compared to WT siblings (Fig. 12H-J, T-V, Table 1). However, there was no statistically significant difference in the number of spinal cord cells expressing *skor1b, skor2, ebf3a* or *neff1* (Fig. 12K-S, W-Y, Table 1). As discussed above, *hmx2* and *hmx3a* are expressed in both dI2 and V1 cells. Therefore, to test which of these two cells types was upregulating expression of *skor1a* and *nefma*, we performed double-labelling experiments with *Tg(pax2a:GFP)*, which, in the spinal cord, is exclusively expressed in V1 cells [6]. We observed no co-expression of GFP and either *skor1a* or *nefma* in either WT siblings or *hmx2;hmx3a* deletion mutant embryos, suggesting that the cells that ectopically express *skor1a* and *nefma* in *hmx2;hmx3a* deletion mutants are dI2 interneurons and not V1 interneurons (Fig. 12Z-AC’”). This suggests that Hmx2/3a normally repress *skor1a* and *nefma* expression in dI2 interneurons. Taken together with our V0v data described earlier in this paper, these data raise the intriguing possibility that Evx1/2 might regulate expression of *skor1a* and *nefma* in V0v interneurons by repressing expression of Hmx3a, which itself normally represses *skor1a* and* nefma.*

## Discussion

### *skor1a, skor1b, skor2, ebf3a *and* neff1* all require Evx1/2 function for their expression in V0v spinal interneurons

**Fig. 13:**
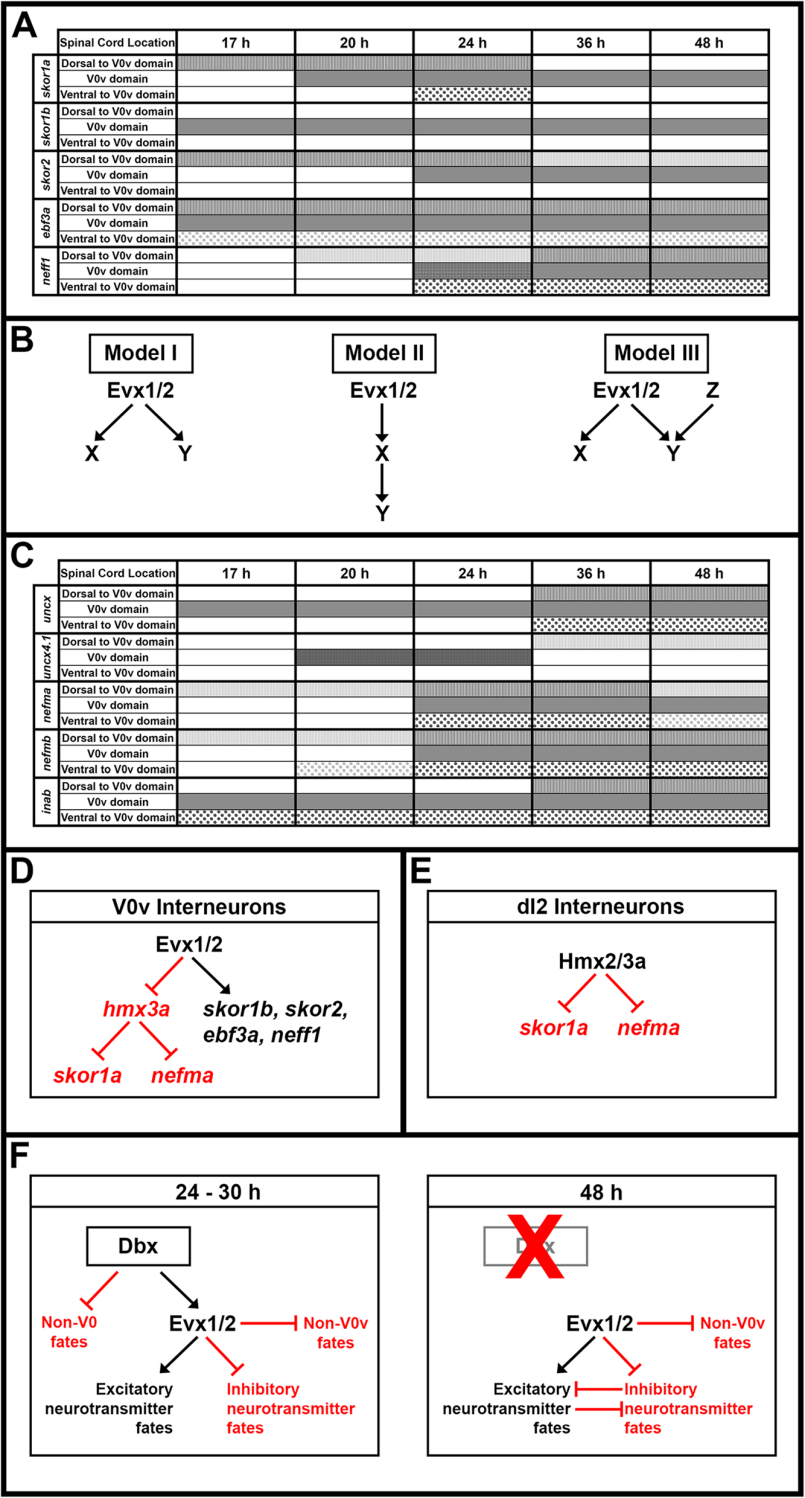
Possible GRNs downstream of Evx1/2 in V0v spinal interneurons. (**A**) Schematic summary of temporal expression profiles of *skor1a* (row 1), *skor1b* (row 2), *skor2* (row 3), *ebf3a* (row 4) and *neff1* (row 5) in the zebrafish spinal cord at 17 h (column 2), 20 h (column 3), 24 h (column 4), 36 h (column 5) and 48 h (column 6) (for in situ hybridization data, please see Fig. 2F-Y, AT-AX). Column 1 lists the location of the expression in the spinal cord. Strong expression in the V0v domain is shown in solid gray. Weak expression in the V0v domain is shown in dark cross-hatching. Expression dorsal to the V0v domain is depicted by either dark (strong expression) or light grey (weak expression) vertical lines. Expression ventral to the V0v domain is represented by either dark (strong expression) or light grey (weak expression) dots. (**B**) Possible models that explain the temporal expression profiles of candidate GRN genes downstream of Evx1/2 in V0v spinal interneurons (Fig. 2). Model I shows parallel pathways of activation of genes X and Y downstream of Evx1/2. This model may explain the activation of genes that are expressed at similar times in V0v interneurons. In contrast, genes that are expressed at different times may be explained by at least two different models. Model II shows a hierarchical pathway of gene activation downstream of Evx1/2. Evx1/2 activates gene X and the protein product of X then activates gene Y. In this case gene X will be expressed before gene Y. Finally, Model III shows the parallel activation of gene Y by both Evx1/2 and the protein product of gene Z. In this case gene Y will only be expressed when Evx1/2 and Y are expressed and if Y is expressed later than Evx1/2, genes activated by this method will be expressed later than genes activated just by Evx1/2. For simplicity, in these models, we are showing a single step direct gene activation for each step of the pathway. However, as studies have shown for other spinal cord neurons, it is possible that V0v spinal interneuron fates are not specified directly but rather, via a repression of repression mechanism (e.g. [10, 74, 93–97]). (**C**) Temporal expression profiles of *uncx* (row 1), *uncx4.1* (row 2), *nefma* (row 3), *nefmb* (row 4) and *inab* (row 5) in the zebrafish spinal cord at 17 h (column 2), 20 h (column 3), 24 h (column 4), 36 h (column 5) and 48 h (column 6) (for in situ hybridization data, please see Fig. 2Z-AS, AY-AAC). Column 1 lists the location of the expression in the spinal cord. Strong expression in the V0v domain is shown in solid gray. Weak expression in the V0v domain is shown in dark cross-hatching. Expression dorsal to the V0v domain is depicted by either dark (strong expression) or light grey (weak expression) vertical lines. Expression ventral to the V0v domain is represented by either dark (strong expression) or light grey (weak expression) dots. (**D**) Our data suggest that Evx1/2 may regulate the expression of *skor1a* and *nefma* in V0v spinal interneurons by repressing the expression of *hmx3a* (shown in red). In contrast, the expression of *skor1b, skor2, ebf3a* and *neff1*, although dependent on Evx1/2, is independent of *hmx3a*. (**E**) Our data suggest that in excitatory dI2 spinal interneurons, *hmx3a* might repress the expression of non-dI2 fates by repressing the expression of *skor1a* and *nefma*. (**F**) Possible model that explains our scRNA-seq data for Mutant Group 3 cells. At 24–30 h, *dbx1a* and *dbx1b* expression persists in zebrafish V0v spinal interneurons as they become post-mitotic and start to differentiate. Together, Dbx and Evx1/2 repress non-V0v fates in WT cells. Evx1/2 are also required to specify the excitatory neurotransmitter fates of WT V0v spinal interneurons and repress inhibitory neurotransmitter fates. In the absence of Evx1/2, at 24–30 h, double mutant *evx1*^*i232/i232*^*;evx2*^*sa140/sa140*^ cells switch their neurotransmitter fates from excitatory to inhibitory, but they do not change their V0v identities (their axon trajectories are unchanged and they do not ectopically express En1b)[14]. In contrast, by 48 h, *dbx1a/b* are no longer expressed in V0v spinal interneurons, and Evx1/2 is now needed to maintain V0v cell fates by repressing other inhibitory interneuron and motoneuron fates. Consequently, double mutant cells begin to transfate and adopt inhibitory, non-V0v fates by 48 h

Taken together, our 30 h in situ hybridization and 48 h scRNA-seq data strongly suggest that *skor1a, skor1b, skor2, ebf3a* and *neff1* are all expressed in V0v spinal interneurons and their expression in these cells requires Evx1/2 function (Figs. 4, 5 and 6, Tables 1 & 2). Therefore, these five genes are all potential members of GRNs downstream of Evx1/2 in V0v interneurons. It is also likely, given that each of these genes has distinct patterns of spinal expression in addition to the V0v domain, that they also have roles in different subsets of other spinal neurons (Fig. 13A & C).

Consistent with this hypothesis, *skor2* and *skor1a*, unlike *skor1b*, are both expressed in the dorsal spinal cord at 17 h, but they have very different expression patterns to each other at 20 h (Fig. 2F-G, P-Q, Fig. 13A), *ebf3a* is expressed in cells ventral and dorsal to the V0v domain at all stages examined (Fig. 2U-Y, Fig. 13A) and *neff1* is expressed in a broad dorsal–ventral stripe of cells at 36 h and 48 h (Fig. 2AW-AX, Fig. 13A). In addition, while the number of spinal cells that express each of these genes is reduced in *evx1;evx2* double mutants, in each case some cells remain (Figs. 4 and 5). These remaining cells are presumably non-V0v cells since Evx1 and Evx2 are transcription factors expressed in all V0v interneurons and no other spinal cord cells. Intriguingly, we observed a larger reduction (49 cells) in the number of spinal cells expressing *skor1b* than *skor1a* or *skor2* (24 cells each) in 30 h *evx1;evx2* double mutants (Fig. 4A-I, Table 1). This suggests that fewer V0v interneurons express *skor1a* and *skor2* than *skor1b* at this stage. This could be because the former two genes are only expressed in distinct subsets of V0v interneurons, or it could reflect the later expression of these genes in V0v cells. i.e., maybe at this stage of development these genes have only turned on in the “older” V0v interneurons and not yet in the “younger” ones. On the other hand, the reduction in the number of spinal cells expressing *ebf3a* in 30 h *evx1;evx2* mutants (25 cells) is very similar to that for *skor1a* and *skor2*, even though our in situ hybridization experiments suggest that *ebf3a* is expressed in V0v interneurons as early as *skor1b* (Fig. 2K, U, Fig. 4J-L, Fig. 13A, Table 1). Interestingly, while our 30 h data suggest that more V0v interneurons express *skor1b* than *skor1a* or *skor2*, at 48 h, *skor2* is expressed in many more V0v interneurons than *skor1a* or *skor1b*, and, while there is not much difference between *skor1a* expression in the two different WT Groups, *skor1b* and *skor2* are expressed by more WT Group 1 cells than WT Group 2 cells (Fig. 6H-J, Table 2). This suggests that *skor* gene expression in V0v interneurons is dynamic, that specific subsets of V0v interneurons express distinct combinations of *skor* genes at different times in development, and that *skor1a* and *skor1b* may only be expressed by V0v interneurons for a relatively short period.

In addition to being expressed in different subsets of other spinal cord cells, these genes also differ in when they are first expressed in the V0v spinal domain. For example, *skor1b* is expressed in the V0v spinal region as early as 17 h (Fig. 2K, Fig. 13A), whereas *skor1a* is not expressed in this region until 20 h and *skor2* not until 24 h (Fig. 2G, R, Fig. 13A). *ebf3a* is, like *skor1b*, expressed in the V0v region as early as 17 h (Fig. 2U, Fig. 13A) whereas *neff1* is not expressed in this spinal cord domain until 24 h (Fig. 2AV, Fig. 13A). This is important because temporal differences in expression may reflect different positions in the hierarchy of interactions that make up GRNs, with earlier expressed genes regulating the expression of later expressed genes (Model II, Fig. 13B and cf. Model I). This could be tested in future work by examining epistatic relationships between these genes. Alternatively, later-expressed genes may require a later-expressed transcription factor in addition to Evx1/2, in order to be expressed (Model III, Fig. 13B and cf. Model II). Interestingly, our data suggest that there is limited overlap between the varied spatial and temporal expression patterns of these genes. This is consistent with a model where different types of spinal interneurons are specified by distinct GRNs, rather than there being a cassette of genes that specifies the same functional characteristic in different neurons.

Currently, there is very little known about the functions of any of these genes in spinal cord development. *neff1* encodes a NIF protein (also referred to as Type IV Intermediate Filament proteins). In mature mammalian CNS, NIF proteins are important for axon function and maintenance, but their functions during development are less clear [98–102]. (See also more detailed discussion of NIF proteins below). Ebf and Skor proteins have been implicated in development of different neurons in the brain (e.g. [63, 65, 67, 103–106]), but very little is known about their spinal cord functions in any vertebrate. Mouse Skor1 is expressed in dI4 and dI5 spinal interneurons, where it binds Lbx1 [107], and human Skor1 has been implicated in Restless legs syndrome (also known as Willis-Ekbom disease) [108]. *Ebf3* is expressed in spinal interneurons in mouse and chick [105, 109] and correct *Ebf3* spinal expression requires Lmx1b in mouse [66]. This is interesting, given that Lmx1ba/b are also downstream of Evx1/2 in zebrafish V0v interneurons [13]. However, none of these previous data indicate what the functions of these transcription factor genes are in V0v spinal interneurons.

Our data, as discussed above, show that all these genes are downstream of Evx1/2 in V0v interneurons. To date, the only abnormal V0v interneuron phenotype that we have found in zebrafish *evx1;evx2* single or double mutants is their change from being glutamatergic to glycinergic [14]. This suggests that these genes may encode members of GRNs that regulate this phenotype. However, it is also possible that some of these genes function downstream of Evx1/2 in other aspects of V0v differentiation and/or function that we have not yet detected. So far, most of the identified transcription factor genes that specify spinal neuron functional characteristics are expressed throughout the stages of development that we examined by in situ hybridization (e.g. [3, 5, 13, 14]). However, *skor2* and *neff1* are not expressed in the V0v spinal cord domain until 24 h (Fig. 2R, AV, Fig. 13A), which is probably too late to be required for specifying V0v cell glutamatergic fates [14]. These genes may, instead, be involved either in maintaining correct neurotransmitter phenotypes, or in specifying later aspects of V0v interneuron development. To test these different hypotheses, future studies will need to analyze the phenotypes of V0v interneurons in mutants for each of these genes.

### Evx1/2 may regulate the expression of *uncx, nefma*, *nefmb,* and *inab* in different ways at different developmental time points

Our data also suggest that *uncx, uncx4.1, nefma*, *nefmb* and *inab* are expressed by V0v interneurons. *uncx* and *inab* are expressed in the V0v spinal cord domain at all the stages that we examined by in situ hybridization as well as being expressed by cells in both scRNA-seq WT clusters at 48 h (Figs. 2Z-AD, AY-AAC, 6L, Q, Fig. 13C, Table 2). *nefma* and *nefmb* are also expressed in the V0v domain at 24 h and at older stages (Fig. 2AL-AN, AQ-AS, Fig. 13C), although *nefma* and *nefmb* are expressed by more WT Group 1 cells that WT Group 2 cells in our scRNA-seq data at 48 h and *nefmb* is only expressed by a few WT cells in this data set (Fig. 6N-O, Table 2). In contrast, *uncx4.1* is only expressed transiently in the V0v spinal cord domain at 20 h and 24 h in our in situ hybridization data, although we do detect a very small number of V0v cells expressing this gene in our 48 h scRNA-seq data (Fig. 2AF-AG, Fig. 6M, Fig. 13C).

It is less clear whether any of these genes require Evx1/2 function for their expression in V0v interneurons. We did not see any obvious difference in *uncx4.1* expression in 30 h *evx1;evx2* double mutants (Fig. 4P-Q) and the only difference in the mutant clusters in the 48 h scRNA-Seq data was a slight, but statistically-significant increase in expression in the Mutant Group 2 cluster compared to the WT Group 2 cluster (Fig. 6M, Table 2). In addition, while the phenotypes of *skor1a, skor1b, skor2, ebf3a* and *neff1* spinal expression in the absence of Evx1/2 function were generally consistent between our experiments at 30 h and 48 h (the one exception being the expansion of *neff1* expression in Mutant Group 3 at 48 h, Fig. 6P, Table 2), this was not the case for *uncx, nefma*, *nefmb* and *inab.* While we saw no statistically significant change in the number of cells expressing *uncx*, *nefmb* or *inab* in 30 h *evx1;evx2* mutants (Figs. 4–5, Table 1), at 48 h we see an increased number of cells expressing *uncx* and *nefmb* in Mutant Group 1 and 2 clusters (although the increase is not statistically significant for *nefmb* in Mutant Group 2) and, in contrast, the expression of *inab* is statistically-significantly reduced in mutant clusters (Fig. 6L, O, Q, Table 2). These differences suggest that the expression of these four genes is regulated differently at these two developmental stages. For example, maybe *inab* only requires Evx1/2 for its expression in V0v interneurons at later stages, and *uncx* and *nefmb* require Evx1/2 to be turned off in V0v interneurons at later stages. However, this does not explain the more surprising difference in *nefma* expression, where we saw a statistically-significant reduction in the number of cells expressing *nefma* in 30 h *evx1;evx2* mutants (Fig. 5A-C, Table 1), but we see a statistically-significant increase in its expression in all three mutant clusters at 48 h (Fig. 6N, Table 2A). While it is possible for transcription factors to function as both activators and repressors of transcription (e.g. [110–118]), it is unusual that transcription factors like Evx1 and Evx2 would be required both to turn a specific gene on, and then to later turn it off, in the same cells.

*uncx* and *uncx4.1* encode paired-type homeodomain transcription factors and while spinal cord expression of *uncx* in zebrafish and mouse, and *uncx4.1* in mouse has previously been reported [119–122], there is currently no data available on the function of these genes in spinal interneurons. *nefma*, *nefmb* and *inab* are, like *neff1*, *NIF* genes. These three genes have distinct expression patterns from each other and from *neff1*. They are all expressed in the V0v spinal cord domain from 24 h onwards, but only *inab* is expressed in this domain at the earlier stages that we examined. All four of these genes are also expressed in other spinal cord domains at all the stages that we analyzed (Fig. 2AJ-AAC, Fig. 13A, C). These expression patterns suggest that all of these genes function not just in V0v interneurons, but also in other distinct subsets of spinal neurons.

As mentioned above, NIF proteins are important for axon function and maintenance at later stages of differentiation in mammals, but a developmental function for these proteins has not yet been described [98–102]. Our data suggest that expression of *nefma*, *nefmb, inab* and *neff1* genes is regulated by Evx1/2 in V0v INs, albeit in different ways. The NIF proteins encoded by these genes have DNA-binding domains [123, 124], and it has been suggested that these domains may regulate gene expression [125, 126]. While these proteins are generally thought to be cytoplasmic, it is possible that either full-length proteins or shorter forms of the proteins enter the nucleus [127, 128]. For example, the N-terminal DNA-binding domain of Vimentin, a different intermediate filament protein, can enter the nucleus and regulate nuclear architecture and chromatin structure [128]. Therefore, it is possible that *neff1* and *nefma,* both of which are expressed by statistically-significantly fewer cells in 30 h *evx1;evx2* mutants*,* are part of the GRN that regulates neurotransmitter properties in V0v interneurons (Fig. 5A-C, G-I, Table 1). If this is not the case, then they likely function downstream of Evx1/2 in a not-yet-identified aspect of V0v interneuron development. However, *evx1;evx2* double mutants have no obvious axon defects during development (we have examined stages up to 48 h; [14]), suggesting that these NIF proteins are not required for axon outgrowth or pathfinding at the stages that we are examining. It is even less clear what the NIF genes that are upregulated in mutant V0v interneurons are doing. Either way, our data suggest that there are novel developmental functions for these genes.

### Additional candidate GRN transcription factor genes downstream of Evx1 and Evx2 in V0v interneurons

Our scRNA-seq data identified 18 additional transcription factors that may be part of V0v GRNs as they are expressed in at least some V0v WT cells and are downregulated in V0v mutant cells (Fig. 9, Table 6). These are *ccdc3a, dachc, luzp1, mycb, nr5a2, pou3f1, pou3f2b, pou3f3b*, *scrt2*, *pou2f2a, pou2f2b, mafba, pbx1b, scrt1a, zfhx3b*, *nr2f5, ebf1a* and *pitx2*. To our knowledge, this is the first report of *pou2f2a* or *pbx1b* expression in the CNS of any animal, and of *ccdc3a, luzp1, pou2f2b* and *ebf1a* in the spinal cord, although brain expression for these genes has been documented [129–132]. In contrast, *dachc, mycb, nr5a2, pou3f1, pou3f2b, pou3f3b, scrt2, mafba, scrt1a, zfhx3b, nr2f5* and *pitx2* have already been shown to be expressed in the V0v spinal domain in zebrafish embryos [130, 133–139]. Notably*, pitx2* and *mafba* are expressed in a narrower dorsal–ventral spinal cord domain than the others, that appears to coincide only with the V0v domain, suggesting that these genes may be expressed specifically in V0v interneurons [137, 139].

Interestingly, in mice, a small subgroup of V0v interneurons (less than 10%) express Pitx2. This compares to 17.45% of the WT cells in our 48 h data (312/933 WT Group 1 cells and 12/924 WT Group 2 cells). In mouse, the Pitx2^+^ V0v interneurons are preferentially clustered around the central canal and can be further subdivided into excitatory cholinergic (V0c) and glutamatergic (V0g) subtypes [140]. Pitx2 has also been shown to be necessary and sufficient to drive expression of the inhibitory neurotransmitter gene GAD1 in *C. elegans* type D GABAergic motor neurons and it is required for the GABAergic differentiation of superior colliculus cells in the mouse brain [141, 142]. In contrast, Pitx2 function in V0v interneurons is still unknown, but as these cells are excitatory, it may be distinct from its role in these other cell types.

Currently, there is not a lot of data on spinal cord functions of most of the other genes. One exception is *scrt2*. When *scrt2* was knocked down with morpholinos in zebrafish embryos, the number of *islet2-*positive motoneurons was increased compared to uninjected controls, whereas the number of glutamatergic Rohon-Beard sensory neurons, and *pax2a-*positive inhibitory interneurons were unchanged (other types of interneurons were not examined) [136]. This is intriguing, given that some of the Mutant Group 3 cells ectopically express motoneuron markers.

We also identified eight transcription factor genes that are upregulated in mutant V0v interneurons at 48 h. *hmx2, hmx3a*, *otpb, znf385c, znf385a*, *zmat4b, bhlhe22* and *irx1a* were all detected in a small number of WT V0v interneurons, and many more mutant cells (Fig. 10, Table 7). As far as we are aware, this is the first report of spinal cord expression of *znf385a*, *znf385c* or *zmat4b*, and the first report of *irx1a* expression in the zebrafish spinal cord (*Irx1* is expressed in the mouse spinal cord next to the hind limb [143]). However, interestingly, *otpb* and *bhlhe22* expression has previously been detected in the zebrafish spinal cord, including in what appears to be the V0v domain [130]. This suggests that these two genes may need Evx1/2 function to be turned off in V0v interneurons. In the zebrafish brain, Otpb is required and sufficient for specifying aspects of a dopaminergic phenotype [144]. *Bhlhe22*, which was previously known as *Bhlhb5*, has essential roles in mouse retinal development [145], axon elongation of corticospinal motor neurons in mouse [146], and survival of inhibitory neurons in the dorsal horn in mouse [147]. In addition, it has been implicated in the formation of dI6, V1 and V2a spinal interneurons in chicken [148]. However, analysis of a zebrafish *bhlhe22* mutant detected no obvious differences in the spinal expression of *en1b*, *evx1* and *vsx2* in mutants compared to WT siblings, suggesting that V0v, V1 and V2a interneurons still formed in normal numbers [149]. Therefore, the roles of these genes in V0v interneurons remain to be discovered.

We were intrigued to discover that *hmx2* and *hmx3a* are expressed in mutant V0v interneurons (Fig. 10B-C, Table 7, Fig. 11A-C, E-E”’, Table 1). In a previous study, we showed that *hmx2* and *hmx3a* are co-expressed in dI2 and V1 spinal interneurons, and that Hmx3a is required for the excitatory fates of dI2 interneurons. (Hmx2 also has a role in this process, but it is much more subtle than that of Hmx3a) [12]. In the absence of Hmx3a function, many dI2 interneurons change their neurotransmitter fates from glutamatergic (excitatory) to GABAergic (inhibitory) [12], suggesting that Hmx3a is required to specify glutamatergic fates in these cells. Therefore, we were surprised that *hmx2* and *hmx3a* are expressed in *evx1;evx2* mutant V0v cells, given that these mutant cells have changed their neurotransmitter phenotype from excitatory to inhibitory. We confirmed this result using a combination of in situ hybridization and immunohistochemistry. We saw a large increase in the number of spinal cells expressing *hmx3a* in *evx1;evx2* double mutants at 30 h (Fig. 11A-C, Table 1). In addition, we used double-labelling experiments to show that V0v spinal interneurons express *hmx3a* in *evx1;evx2* double mutants but not WT siblings (Fig. 11D-E’”). It is likely that these results reflect ectopic expression of *hmx3a* in mutant V0v interneurons, rather than *hmx3a* expression in cells that have transfated into dI2 or V1 interneurons (see discussion of Mutant Group 3 cells below), as we see the expanded *hmx3a* expression as early as 30 h (whereas we do not see expanded expression of other cell fate markers like *en1b* at these earlier stages [14]), and in our scRNA-seq data, we predominantly detect expression of *hmx3a* in Mutant Groups 1 and 2 rather than Mutant Group 3 (Fig. 10C). This temporal difference in *hmx3a* expression in *evx1;evx2* double mutants (expanded at 30 h but not present in Mutant Group 3 cells at 48 h) is probably a consequence of the double mutant cells transfating into inhibitory spinal cells types by 48 h (see discussion below).

Taken together, these data suggest that Evx1/2 repress *hmx3a* expression in V0v interneurons. Therefore, we wondered if any of the genes that require Evx1/2 for their expression in V0v cells might be repressed by Hmx2/3a. Interestingly, our data suggest that two of the six genes that we identified as requiring Evx1/2 function at 30 h, *skor1a* and *nefma*, are upregulated in dI2 interneurons in *hmx2;hmx3a* deletion mutants (Fig. 4A-C, Fig. 5A-C, Fig. 12H-J, T-V, Z-AC’”, Table 1), suggesting that Hmx2/3a may usually repress these genes in both dI2 and V0v interneurons (Fig. 13D-E). However, in contrast, there is no statistically significant change in the number of spinal cord cells expressing *skor1b*, *skor2*, *ebf3a* or *neff1* in *hmx2;hmx3a* deletion mutants (Fig. 12K-S, W-Y, Table 1). In combination, these data suggest that there are at least two distinct GRNs downstream of Evx1/2 in V0v neurons, one that includes repression of Hmx2/3a and one that is independent of Hmx2/3a (Fig. 13D).

### Two molecularly distinct subsets of WT V0v interneurons exist at 48 h

Our scRNA-seq analysis of V0v interneurons in embryos from an incross of *evx1;evx2* heterozygous mutant parents, identified five distinct clusters of cells. Based on our differential gene expression analyses, two are likely to be distinct WT clusters and the other three distinct mutant clusters (Fig. 6A). As discussed in the Results, there are 15% more cells than we would expect in the WT clusters (1857 observed versus 1609 expected WT cells), compared to the mutant clusters (1003 observed versus 1251 expected mutant cells). This suggests that either the mutant cells were more fragile and, therefore, had a higher probability of not making it into our data set, or some of the mutant cells are contained in what we have defined as the WT clusters. Both these explanations are possible. Due to their altered expression profiles, the mutant cells might be more likely to lose their integrity / become sick, in which case they would have been excluded from our analyses. It is also possible that some of the mutant cells have WT-like phenotypes and ended up in the WT clusters. Our previous analyses demonstrate that the V0v phenotypes of *evx1* and *evx2* single mutants are not completely penetrant. In single mutants, not all mutant cells lose expression of the genes that we analyzed, whereas more cells lose expression of these genes in double mutants [14]. If this lack of penetrance persists to 48 h, we would expect some mutant cells to have a “WT” phenotype. Consistent with this, a small number of cells in the WT clusters in our UMAP plots appear to have partial mutant phenotypes where they express *evx1* and/or *evx2* but also express inhibitory genes, or they express markers of both glutamatergic and inhibitory fates (Supp. Figure 3).

Our discovery of two distinct subtypes of V0v interneurons at 48 h, is consistent with the existence of distinct molecular and/or functional subtypes of V0v interneurons in mouse [17, 140] and adult zebrafish [150], and further highlights the conservation of neuronal specification between zebrafish and mammals. While we did not observe any obvious subtypes of V0v interneurons in our analyses of these cells at earlier developmental stages [14], the Higashijima group has previously identified three subsets of V0v interneurons with distinct morphologies that form at different times during the first four days of development [151]. V0-eA (commissural ascending) interneurons form first [151], and these correspond morphologically to the neurons that we previously analyzed [14]. At later stages V0-eB (commissural bifurcating), and then V0-eD (commissural descending) cells develop [151]. These researchers reliably detected neurons with a V0-eB morphology at 60 h and neurons with a V0-eD morphology at 84 h [151]. However, BrdU-labelling experiments showed that most V0-eB neurons are post-mitotic by 30–36 h. In contrast, most V0-eD neurons are not post-mitotic until 42–48 h [151]. Therefore, it is possible that our WT Groups 1 and 2 correspond to V0-eA and V0-eB cells. Consistent with this, the gene expression profile of the WT Group 1 cluster at 48 h more closely resembles what we saw at 27–30 h than WT Group 2. There is statistically-significantly more expression of *skor1b*, *skor2*, *ebf3a*, *nefma*, *nefmb*, *uncx* and *neff1* in WT Group 1 than WT Group 2 cells (Fig. 6I-L, N-P, Table 2A). As mentioned earlier, a small subset of V0v interneurons in mouse are cholinergic [140]. However, it is unlikely that either of our WT Groups correspond to these cells as both WT Group 1 and WT Group 2 contain too many cells and, in addition, we do not detect any expression of *chatb* in any of the cells in our 48 h data set and we only detect *chata* expression in a small subset of WT Group 1 cells (6.75% (63/933 cells)) and a very small number of WT Group 2 and Mutant Group 3 cells (0.97% (9/924 cells) and 5.97% (12/201 cells) respectively, data not shown).

### *evx1;evx2* double mutant cells may transfate into distinct inhibitory interneurons, or motoneurons

The most surprising result from our scRNA-seq analyses is the phenotype of the cells in the Mutant Group 3 cluster. The lack of *evx1*, *evx2* and *slc17a6a* expression in these cells and the increase in expression of markers of inhibitory cells, including *slc6a5, slc6a1b* and *gad1b* suggest that this cluster contains the most severe mutant cells, which presumably are the double mutant cells (Fig. 6A-G, Table 2). As discussed in the Results section, the number of cells in this cluster is consistent with this hypothesis. However, the phenotype of these cells at 48 h is surprising, compared to what we have seen using in situ hybridization at earlier stages of development (Fig. 5G-I, Fig. 6L, P, Table 1, Table 2). The phenotype of Mutant Group 3 cells is distinct from the other two Mutant Groups: distinct subsets of Mutant Group 3 cells express markers of either different types of inhibitory spinal cord interneurons, or motoneurons (Fig. 8, Table 3, Table 5).

One possible explanation of these different phenotypes is that the Mutant Group 3 cells are changing their gene expression profiles not because of their genotype, but as a side-effect of the experimental procedures. That some aspect of the experiment, for example the cell dissociation, has caused a subset of cells to aberrantly turn on genes that they wouldn’t normally express. For example, where we just see a few cells expressing a particular gene, we cannot completely rule out the possibility that this is just noise in the experiment. However, if this was the case, we would expect to see a wider variety of genes mis-expressed. We also might expect the cells to be sick, whereas all these cells appear healthy as they express very low levels of mitochondrial transcripts, typical of healthy cells (< 6%, data not shown), and pass all other stringent scRNA-seq quality controls (see Methods for further information). In addition, the cell numbers are not consistent with this hypothesis. If this was the case, and cells of all genotypes were equally likely to be affected this way, then we would expect the ratio of WT cells (Group 1 and Group 2) to mutant cells in Mutant Group clusters 1 and 2 to be 9:7 (this assumes that the two Mutant Groups include both single and double mutant cells). Therefore, we would predict there to be 1496 WT cells and 1163 mutant cells. However, what we observe is 1857 cells in WT Groups 1 and 2 and 802 cells in Mutant Groups 1 and 2 (*P* value for Chi squared test < 0.0001).

We consider that it is much more likely that the Mutant Group 3 cluster consists of double mutant cells, and that by 48 h, these cells have started to transfate into different types of spinal neurons. This seems the most parsimonious explanation given that there are several distinct (non-overlapping) sub-clusters of 20 or more cells each in Mutant Group 3 in the UMAP analysis, and the expression of each gene that defines these sub-clusters is statistically significantly higher in Mutant Group 3 compared to all other clusters (Fig. 8, Table 3, Table 5). As described in the Methods section of the paper, we used ANOVA to analyze these data as Nault and colleagues [55] have shown that it is the best method for calculating differential expression in scRNA-seq data when cell numbers are small. Given that none of the potential ectopic fates that we observe are glutamatergic, and that they are instead inhibitory or cholinergic, this would also suggest that there is a feedback mechanism between neurotransmitter phenotype and cell type identity / cell fate. This hypothesis could also explain the large number of cells in Mutant Group 3 that express *neff1*, even though expression of this gene is downregulated in Mutant Groups 1 and 2, as *neff1* is broadly expressed in the spinal cord at 48 h, and it is possible that it is expressed by motoneurons and/or inhibitory interneurons at this stage (Fig. 2AX, Fig. 6P, Table 2A).

Several studies have suggested that spinal cord fates are specified via a repression of repression mechanism, where fate-specific genes inhibit all other possible fates rather than directly specifying the fate in question (e.g. [10, 74, 93–97]). Our data is similarly consistent with a mechanism where loss of Evx1/2 allows other non-V0v-cell fate-specifying genes to be expressed (Fig. 13F). Our analyses suggest that these different fates are non-overlapping, which would be consistent with a model where, as cells start to express one of these other fate-specifying genes, that gene then represses expression of other fate-specifying genes. Such a mechanism could stochastically produce different subsets of cells with distinct fates. It is currently not clear why these different ectopic fates are restricted to motoneurons and inhibitory interneurons. However, the first phenotype that we observe in *evx1;evx2* mutants is the loss of glutamatergic markers and gain of inhibitory markers. This suggests the intriguing hypothesis that there is something about this early change in neurotransmitter phenotypes that influences the later change in other aspects of cell fate (Fig. 13F).

Interestingly, in mouse *Evx1* mutants, expression of Evx2 is also lost in the spinal cord and about two thirds of V0v interneurons transfate into V1 interneurons, based on their expression of En1 (which, in the spinal cord, is only expressed by V1 cells) and their changed migration pattern and axon trajectories [17, 71]. While this previous mouse study did not examine neurotransmitter phenotypes, V1 interneurons are inhibitory [71, 152, 153]. In our earlier analyses of zebrafish *evx1;evx2* double mutants, we saw no evidence of V0v interneurons ectopically expressing *en1b* or Pax2 at 24 h or 30 h (Pax2 is expressed by V1 and several other inhibitory spinal interneurons) or changing their axon trajectories at 27 h or 48 h [14]. However, our data in this paper suggest that *evx1;evx2* double mutant V0v interneurons express markers of different inhibitory spinal neuron or motoneuron fates by 48 h. 28.36% (57/201 cells) of Mutant Group 3 cells express *en1b,* suggesting that almost a third of the double mutant cells may be transfating to V1 cells, compared to two thirds in the mouse study (Table 3). We do not think that we missed an increase in *en1b* expression in our earlier analyses as we saw a reduction, albeit not statistically significant, in the number of spinal cells expressing *en1b* at 24 h [14]. Previously, we suggested that the mouse and zebrafish phenotypes might be different because *dbx* expression persists in at least some V0v cells in zebrafish [14]. This might also explain why zebrafish *evx1;evx2* double mutant V0v cells still develop V0v morphologies and do not initially express genes associated with other spinal cord fates. V0v and V0_D_ interneurons both develop from *dbx*-expressing progenitor cells, and they have similar axon trajectories [17, 25, 26]. Therefore, it is possible that Evx1/2 are initially just required to specify the glutamatergic phenotype of V0v interneurons (V0_D_ neurons do not express Evx1/2 and are inhibitory), as Dbx can initially repress non-V0 cell fates. However, as Dbx function wanes, Evx1/2 may also be required to maintain V0v identities by repressing other interneuron and motoneuron identities (Fig. 13F). It remains unclear why more V0v cells acquire V1 fates in mouse than in zebrafish, although it is possible that this is also due to temporal differences in when Evx1/2 is required to maintain V0v identities.

## Conclusions

In conclusion, this paper identifies two molecularly distinct subtypes of WT V0v spinal interneurons at 48 h. We also identify 25 transcriptional regulators that are downstream of Evx1/2 in V0v spinal interneurons at 30 h and/or 48 h that are, therefore, strong candidates for being members of the GRNs that specify the functional characteristics of these cells, plus 11 transcriptional regulators that are upregulated in V0v spinal interneurons at 48 h when Evx1/2 activity is reduced (*nefma* is in both of these groups as it is downregulated in e*vx1;evx2* mutants at 30 h and upregulated at 48 h). Interestingly, two of the transcriptional regulators that are upregulated in *evx1/2* mutants are *hmx2* and *hmx3a*, and we show that Hmx2/3a, in turn, repress expression of *skor1a* and *nefma* in dI2 interneurons. This suggests that Evx1/2 might regulate *skor1a* and *nefma* expression in V0v interneurons by repressing Hmx2/3a expression. Finally, our data suggest that in the absence of both Evx1 and Evx2, V0v spinal interneurons initially change their neurotransmitter phenotypes from excitatory to inhibitory and then at a later point of development, transfate into distinct types of inhibitory spinal interneurons, or motoneurons. Taken together, these findings significantly increase our knowledge of V0v spinal development and move us towards a greater understanding of the GRNs that regulate this essential process.

### Supplementary Information

**Additional file 1: Supplementary Figure 1.***inaa* and *ebf3a* Genes are Not Expressed in Zebrafish Spinal Cord. (A-D) Lateral views of 30 h WT (A, C) embryos or (B, D) high magnification views of head regions indicated with black dotted boxes in A and C respectively. Rostral, left. Dorsal, up. Neither (A)* inaa* nor (C) *ebf3b* are expressed in spinal cord. Both genes are only expressed in a small subset of cells in the dorsal telencephalon (*, A-D). These embryos were over-stained to try and detect any weak expression that might be present. The low-level background expression is probably due to probe-trapping in the CNS ventricles and other tissues. While we cannot unequivocally rule out that (A) *inaa* and (C) *ebf3b* are broadly or ubiquitously expressed in the spinal cord, we think this is highly unlikely given the high intensity, specific staining of both genes in the brain. (A’) Heatmap ANOVA analysis of *inaa* expression in different FAC-sorted populations of cells. Class 1: All trunk cells. Class 2: All post-mitotic spinal neurons. Class 3: V0v interneurons. Each square is a different biological replicate. The relative expression levels of *inaa* are shown as normalized data transformed to a mean of 0, with standard deviation of +1 (highly expressed, red) or -1 (weakly/not expressed, blue) sigma units. The *P-*value (left-hand side) is corrected for multiple testing. *inaa* is not reproducibly expressed in either all neurons (Class 2) or all V0v spinal interneuron (Class 3) samples. This analysis is not provided for *ebf3b* because this gene was not present on our microarray. Scale bar: (A, C) 200 µm, (B, D) 70 µm.

**Additional file 2: Supplementary Figure 2.***nefla* and *neflb* are Not Expressed in V0v Spinal Interneurons. (A) Heatmap ANOVA analysis of *nefla* and *neflb* expression in different FAC-sorted populations of cells. Class 1: All trunk cells. Class 2: All post-mitotic spinal neurons. Class 3: V0v interneurons. Each column is a different biological replicate. Rows show relative expression levels for the gene in question as normalized data transformed to a mean of 0, with standard deviation of +1 (highly expressed, red) or -1 (weakly/not expressed, blue) sigma units. *P-*values (left-hand side) are corrected for multiple testing. *nefla* and *neflb* are expressed in some post-mitotic spinal interneurons (Class 2) but not in V0v spinal interneurons (Class 3). (B, C, E, F) Lateral views of (B, E) WT and (C, F) *evx1*^*i232;i232*^*;evx2*^*sa140;sa140*^ double mutant embryos (labeled *evx1;evx2*) at 30 h. Rostral, left. Dorsal, up. (D, G) Number of cells expressing (D) *nefla* and (G) *neflb* in a precisely-defined spinal cord region adjacent to somites 6-10 at 30 h. Data are depicted as individual value plots and *n-*values are shown below. For each plot, the wider red horizontal bar depicts the mean number of cells, and the red vertical bar depicts the S.E.M. (these values are listed in Table 1). All counts are an average of at least four embryos. White circles indicate WT data and black circles indicate *evx1;evx2* double mutant data. All data were analyzed for normality using the Shapiro-Wilk test. Data sets in D and G are normally distributed and so the F-test for equal variances was performed, followed by a type 2 Student’s *t*-test (for equal variances). *P*-values are provided in Table 1. (D, G) There is no statistically significant difference in the number of spinal interneurons expressing either (D) *nefla* or (G) *neflb* in *evx1;evx2* double mutant embryos, compared to WT embryos. Scale bar: 50 µm.

**Additional file 3: Supplementary Figure 3.** Three-way Differential Gene Expression of WT and *evx1*/2 Mutant V0v Interneurons. (A) For ease of cell type comparison, panel Supp. Fig. 3A has been reproduced from Fig. 6A. 2D UMAP plot of 48 h post-mitotic V0v spinal interneuron single-cell RNA-seq atlas (2860 cells). Cells were obtained from 48 h embryos produced from an incross of *evx1*^*i232/+*^*;evx2*^*sa140/+*^ heterozygous parents homozygous for *Tg(evx1:EGFP)*^*SU2*^. Clusters are color-coded by cell identity: V0v WT Group 1 (light green), V0v WT Group 2 (dark green), V0v Mutant Group 1 (turquoise), V0v Mutant Group 2 (light blue), and V0v Mutant Group 3 (dark blue). Panel (B) indicates the colour-coding for panels (C-D). This has been reproduced from Fig. 8S. (C-D) Cells expressing only gene 1 are green. Cells expressing only gene 2 are red. Cells expressing only gene 3 are blue. Cells are yellow, pink, or turquoise if they co-express genes 1 and 2, genes 2 and 3, and genes 1 and 3 respectively. Cells expressing all three genes are white. Black shows cells with no expression detected for all three genes of interest. All expression data have been normalization (see Methods). (C) A very small subset of WT cells co-express *evx1, evx2 *and the glycinergic inhibitory marker *slc6a5* (white cells). (D) Similarly, a very small subset of WT cells co-express markers of both glutamatergic excitatory (*slc17a6a*) and glycinergic inhibitory (*slc6a5*) phenotypes, together with *evx2* (white cells).

**Additional file 4: Supplementary Figure 4. ***Tg(hmx CNEIII:cos:Gal4-VP16,UAS:EGFP)*^*SU41*^ Recapitulates Endogenous *hmx3a* Expression in the Zebrafish Spinal Cord. (A) Schematic showing Shuffle-LAGAN analysis of the contiguous *hmx3a-hmx2* genomic region with zebrafish sequence as the baseline, compared to orthologous genomic regions in *H. sapiens* (row 1), *M. musculus *(row 2), *G. gallus *(row 3), and *X. tropicalis* (row 4). Conserved exonic coding sequences are shown in dark blue. Conserved exonic untranslated sequences are shown in light blue. Grey arrows indicate 5’-3’ orientation. Conserved Non-coding Elements (CNEs) upstream of *hmx3a* (CNE I and II), and intergenic between *hmx3a* and *hmx2* (CNE III), are shown in pink. The genomic amplicons used for transgenic testing are indicated with red dotted boxes. Only the transgenic line created with CNE III (*Tg(hmx CNEIII:cos:Gal4-VP16,UAS:EGFP)*^*SU41*^) showed EGFP expression in the spinal cord similar to endogenous *hmx3a* expression (see Methods). This line was used for the experiments described in this paper. (B-B’’’) Lateral views of WT spinal cord at 27 h. Rostral, left. Dorsal, up. (B’) in situ hybridization for *hmx3a* is shown in red. (B’’) Immunohistochemistry for *Tg(hmx CNEIII:cos:Gal4-VP16,UAS:EGFP)*^*SU41*^ is shown in green. (B, B’’’) Merged images. (B) maximum intensity projection image. (B’-B’’’) high-magnification single confocal planes of the region indicated by white dotted box in B. In zebrafish spinal cord, *hmx3a* mRNA is exclusively expressed by V1 and dI2 interneurons (12)). (B’’’) All *hmx3a*-expressing spinal interneurons co-express *Tg(hmx CNEIII:cfos:Gal4:UAS:EGFP)*^*SU41*^ (white asterisks). (C) Quantitative RT-PCR indicates that *hmx2, hmx3a, slc17a6b* and *slc32a1* expression is enriched in *Tg(hmx CNEIII:cfos:Gal4-VP16,UAS:EGFP)*^*SU41*^-expressing cells (blue) compared to non-EGFP-expressing cells (red). Cells were isolated via FACS from 27 h transgenic embryos. This data further suggests that *Tg(hmx CNEIII:cos:Gal4-VP16,UAS:EGFP)*^*SU41*^ recapitulates endogenous *hmx3a* expression in *slc17a6b-*positive (excitatory) dI2 and *slc32a1­-*positive (inhibitory) V1 cells that also specifically co-express *hmx2* and *hmx3a* in the zebrafish spinal cord. Scale bar: (B) 50 µm, (B’-B’’’) 25 µm.

**Additional file 5: Supplementary Table 1.** Gene Names, Previous Names, ZFIN Identifiers, Primer Sequences and References for in situ Hybridization Probes. Column 1 lists genes used in this study. Previous names, where known, are provided in column 2. Column 3 contains the unique ZFIN identification number for each gene. Columns 4-6, where relevant, show the primer sequences and expected product sizes (in base pairs (bp)) respectively, used to generate templates for anti-sense RNA riboprobe synthesis from 27 h WT cDNA and the annealing temperature used in the polymerase chain reaction. For further conditions for riboprobe synthesis, please see Methods. Column 7 provides the reference for the in situ hybridization RNA riboprobe used in our experiments.

**Additional file 6: Supplementary Table 2.** Hurdle Model Statistical Analyses of Differential Expression in Our Single-Cell RNA-Seq Atlas of V0v Spinal Interneurons From an Incross of Zebrafish *evx1*^*i232/+*^*;evx2*^*sa140/+*^ Heterozygous Parents. Statistically robust Hurdle modelling was performed to analyze differential gene expression between distinct cell populations in our 48 h post-mitotic V0v spinal interneuron single-cell RNA-seq atlas (see Fig 6A and Methods). Each Excel sheet corresponds to a distinct Hurdle model statistical comparison of differential gene expression. The specific comparison is indicated on the Excel page tab. WT1: WT Group 1; WT2: WT Group 2; M1: Mutant Group 1; M2: Mutant Group 2; M3: Mutant Group 3. On each page, column A lists the gene ID from the Lawson Lab zebrafish transcriptome annotation model V4.3.2 (50). Column B lists the gene symbol. (Note that *skor2, neff1, isl1a, pou2f2a, pou2f2b* and *zfhx3b* genes returned previous gene names in the Lawson annotation. For ease of comparison, the current gene names shown in this paper are given in column B, with the Lawson gene symbols shown in parentheses). Column C provides the *P*-value for the comparison. This has not been corrected for multiple testing. Column D provides the *P*-value corrected for multiple testing by the application of the False Discovery Rate (FDR) Benjamini Hochberg method (46). Column E shows the ratio of the least squares mean reads for the antecedent (first population, column G) versus the consequent (second population, column H) population in the comparison. Column F shows the fold-change. The fold-change is converted from the ratio values in column E. When the ratio in column E is greater than 1, the fold-change in column F is identical to the ratio in column E. When the ratio in column E is less than 1, the fold-change in column F is calculated using the formula: -1/ratio. Each row corresponds to a distinct gene symbol. Each sheet has been sorted by the fold-change value in column F, from smallest to largest value. Consequently, negative fold-change values are shown at the top of the sheet, followed by positive fold-change values in the middle, and N.C. fold-change values at the bottom of the sheet. Negative fold-change values occur when the least squares mean reads for the antecedent (first population in the comparison, column G) is less than the least squares mean reads for the consequent (second population in the comparison, column H), and so expression of that gene is upregulated in the consequent (second) population in the comparison. In contrast, positive fold-change values occur when the least squares mean reads for the antecedent (first population in the comparison, column G) is greater than the least squares mean reads for the consequent (second population in the comparison, column H), and so expression of that gene is upregulated in the antecedent (first) population in the comparison. N.C. The Hurdle model of differential expression analysis cannot be calculated. Usually this is because there is no or little expression in one population in the comparison.

**Additional file 7: Supplementary Table 3. **ANOVA Statistical Analyses of Differential Expression in Our Single-Cell RNA-Seq Atlas of V0v Spinal Interneurons From an Incross of Zebrafish *evx1*^*i232/+*^*;evx2*^*sa140/+*^ Heterozygous Parents. ANOVA statistical modelling was also performed to aid inference of differential gene expression between distinct cell populations in our 48 h post-mitotic V0v spinal interneuron single-cell RNA-seq atlas (see Fig 6A and Methods). Each Excel sheet corresponds to a distinct ANOVA statistical comparison of differential gene expression. The specific comparison is indicated on the Excel page tab. WT1: WT Group 1; WT2: WT Group 2; M1: Mutant Group 1; M2: Mutant Group 2; M3: Mutant Group 3. On each page, column A lists the gene ID from the Lawson Lab zebrafish transcriptome annotation model V4.3.2 (50). Column B lists the gene symbol. (Note that *skor2, neff1, isl1a, pou2f2a, pou2f2b* and *zfhx3b* genes returned previous gene names in the Lawson annotation. For ease of comparison, the current gene names shown in this paper are given in column B, with the Lawson gene symbols shown in parentheses). Column C provides the *P*-value for the comparison. This has not been corrected for multiple testing. Column D provides the *P*-value corrected for multiple testing by the application of the False Discovery Rate (FDR) Benjamini Hochberg method (46). Column E gives the t-value. This is the size of the difference between the populations in the comparison relative to the variation in the data. The t-value is given in units of standard error. Column F shows the ratio of the least squares mean reads for the antecedent (first population, column H) versus the consequent (second population, column I) population in the comparison. Column G shows the fold-change. The fold-change is converted from the ratio values in column F. When the ratio in column F is greater than 1, the fold-change in column G is identical to the ratio in column F. When the ratio in column F is less than 1, the fold-change in column G is calculated using the formula: -1/ratio. Each row corresponds to a distinct gene symbol. Each sheet has been sorted by the fold-change value in column G, from smallest to largest value. Consequently, negative fold-change values are shown at the top of the sheet, followed by positive fold-change values in the middle, and N.C. fold-change values at the bottom of the sheet. Negative fold-change values occur when the least squares mean reads for the antecedent (first population in the comparison, column H) is less than the least squares mean reads for the consequent (second population in the comparison, column I), and so expression of that gene is upregulated in the consequent (second) population in the comparison. In contrast, positive fold-change values occur when the least squares mean reads for the antecedent (first population in the comparison, column H) is greater than the least squares mean reads for the consequent (second population in the comparison, column I), and so expression of that gene is upregulated in the antecedent (first) population in the comparison. N.C. The ANOVA model of differential expression analysis cannot be calculated.

**Additional file 8: Supplementary Table 4.** Gene-Specific Analysis of Differential Expression in 27 h *Tg(hmx CNEIII:cos:Gal4-VP16,UAS:EGFP)*^*SU41*^-expressing V1 and dI2 Spinal Cord Interneurons Isolated From Uninjected Control and *hmx2;hmx3a* Double Morphant Embryos. Gene-Specific Analysis (GSA) was performed to analyze differential gene expression in V1 and dI2 spinal cord interneurons between 27 h uninjected control and *hmx2;hmx3a* double morphant samples (see Methods). Column A lists the gene symbol. Note that *skor2, neff1, isl1a, pou2f2a, pou2f2b* and *zfhx3b* genes returned previous gene names in the Lawson annotation. For ease of comparison, the current gene names shown in this paper are given in column A, with the Lawson gene symbols shown in parentheses. Column B provides the *P*-value for the comparison. This has not been corrected for multiple testing. Column C provides the *P*-value corrected for multiple testing by the application of the False Discovery Rate (FDR) Benjamini Hochberg method (46). Column D shows the ratio of the least squares mean reads for the antecedent (first population, column F) versus the consequent (second population, column G) population in the comparison. Column E shows the fold-change. The fold-change is converted from the ratio values in column D. When the ratio in column D is greater than 1, the fold-change in column E is identical to the ratio in column D. When the ratio in column D is less than 1, the fold-change in column E is calculated using the formula: -1/ratio. Each row corresponds to a distinct gene symbol. Each sheet has been sorted by the fold-change value in column E, from smallest to largest value. Consequently, negative fold-change values are shown at the top of the sheet, with positive fold-change values at the bottom of the sheet. Negative fold-change values occur when the least squares mean reads for the antecedent (first population in the comparison, column F) is less than the least squares mean reads for the consequent (second population in the comparison, column G), and so expression of that gene is upregulated in the consequent (second) population in the comparison. In contrast, positive fold-change values occur when the least squares mean reads for the antecedent (first population in the comparison, column F) is greater than the least squares mean reads for the consequent (second population in the comparison, column G), and so expression of that gene is upregulated in the antecedent (first) population in the comparison.

## Data Availability

Microarray data were previously deposited in the NCBI Gene Expression Omnibus with accession number GSE145916. Single-cell and bulk RNA-Seq data have been deposited in the NCBI Gene Expression Omnibus with accession numbers GSE240239 and GSE240238 respectively.

## Abbreviations

BSA: Bovine Serum Albumin
cDNA: Complementary Deoxyribonucleic Acid
CNS: Central Nervous System
DIC: Differential Interference Contrast
DKD: Double Knock-Down (co-injection of morpholinos targeting two different genes)
DPBS: Dulbecco’s Phosphate-Buffered Saline
EGFP: Enhanced Green Fluorescent Protein
FACS: Fluorescent-Activated Cell Sorting
FBS: Foetal Bovine Serum
GRN: Gene Regulatory Network
GSA: Gene-Specific Analysis
h: Hours post fertilization
IACUC: Institutional Animal Care and Use Committee
IOS: Integrative Organismal Systems
NBT/BCIP: Nitro-Blue Tetrazolium/5-Bromo-4-Chloro-3’-Indolyphosphate
N.C.: Not Calculated
NIF: Neuronal Intermediate Filament
N.S.: Data Not Shown
NSF: National Science Foundation
PBS: Phosphate-Buffered Saline
PBST: Phosphate-Buffered Saline + 0.1% Tween-20
PCA: Principal Components Analysis
PCR: Polymerase Chain Reaction
PTU: 1-Phenyl-2-thiourea
qRT-PCR: Quantitative Reverse Transcriptase Polymerase Chain Reaction
RCF: Relative Centrifugal Force
RIN: RNA Integrity Number
S.E.M.: Standard Error of the Mean
TMM: Trimmed Means of M-Values
UMAP: Uniform Manifold Approximation and Projection
UTR: Untranslated Region
WT: Wild-Type
