# Supplementary figures and images for: Molecular analyses of zebrafish V0v spinal interneurons and identification of transcriptional regulators downstream of Evx1 and Evx2 in these cells
Source: Neural Dev. 2023 Nov 28;18:8. doi: 10.1186/s13064-023-00176-w (PMC10683209; doi:10.1186/s13064-023-00176-w)

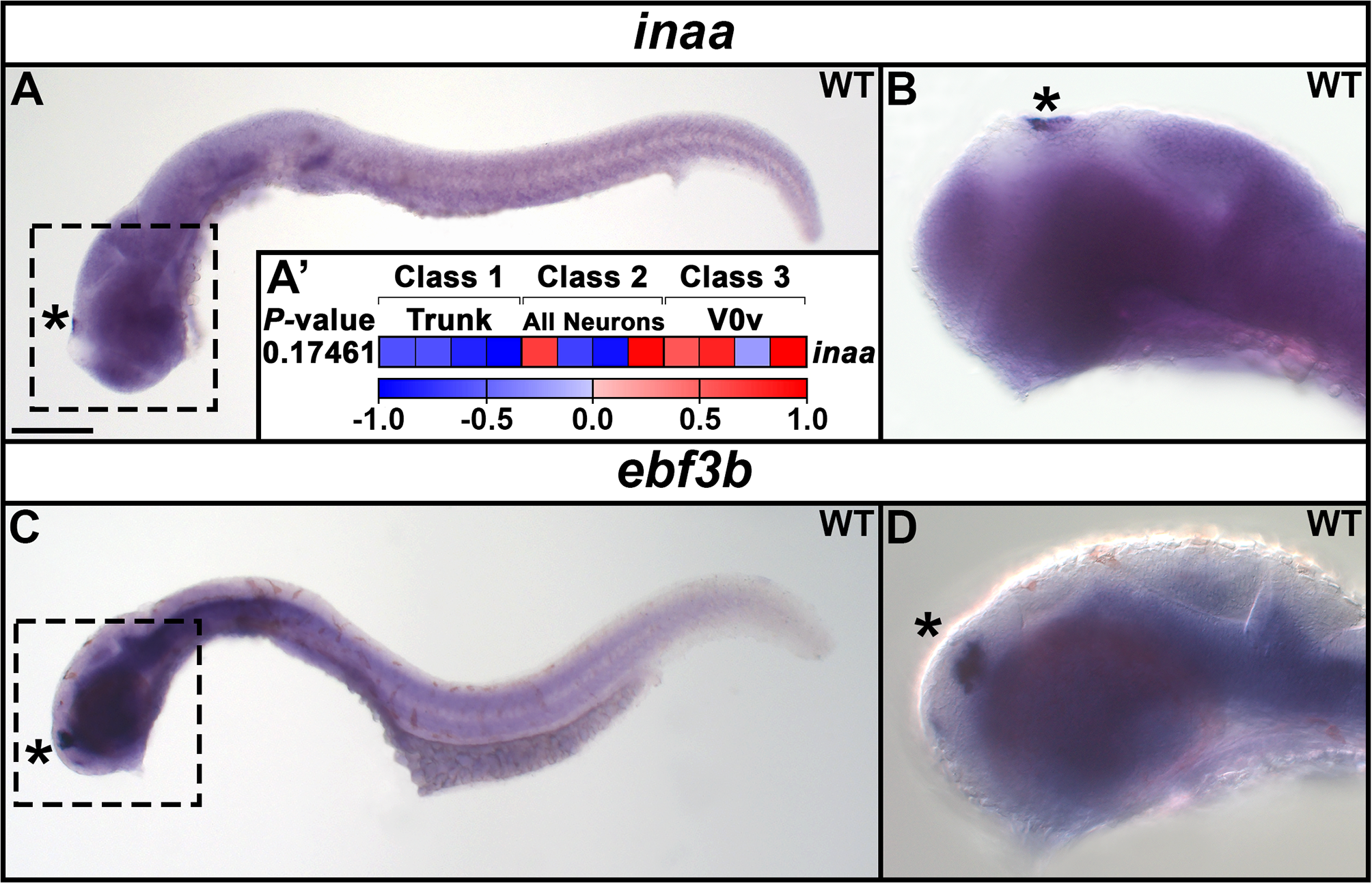

Supplement: Supplementary file 1 — Additional file 1: Supplementary Figure 1.inaa and ebf3a Genes are Not Expressed in Zebrafish Spinal Cord. (A-D) Lateral views of 30 h WT (A, C) embryos or (B, D) high magnification views of head regions indicated with black dotted boxes in A and C respectively. Rostral, left. Dorsal, up. Neither (A) inaa nor (C) ebf3b are expressed in spinal cord. Both genes are only expressed in a small subset of cells in the dorsal telencephalon (*, A-D). These embryos were over-stained to try and detect any weak expression that might be present. The low-level background expression is probably due to probe-trapping in the CNS ventricles and other tissues. While we cannot unequivocally rule out that (A) inaa and (C) ebf3b are broadly or ubiquitously expressed in the spinal cord, we think this is highly unlikely given the high intensity, specific staining of both genes in the brain. (A’) Heatmap ANOVA analysis of inaa expression in different FAC-sorted populations of cells. Class 1: All trunk cells. Class 2: All post-mitotic spinal neurons. Class 3: V0v interneurons. Each square is a different biological replicate. The relative expression levels of inaa are shown as normalized data transformed to a mean of 0, with standard deviation of +1 (highly expressed, red) or -1 (weakly/not expressed, blue) sigma units. The P-value (left-hand side) is corrected for multiple testing. inaa is not reproducibly expressed in either all neurons (Class 2) or all V0v spinal interneuron (Class 3) samples. This analysis is not provided for ebf3b because this gene was not present on our microarray. Scale bar: (A, C) 200 µm, (B, D) 70 µm. [file 13064_2023_176_MOESM1_ESM.tif]

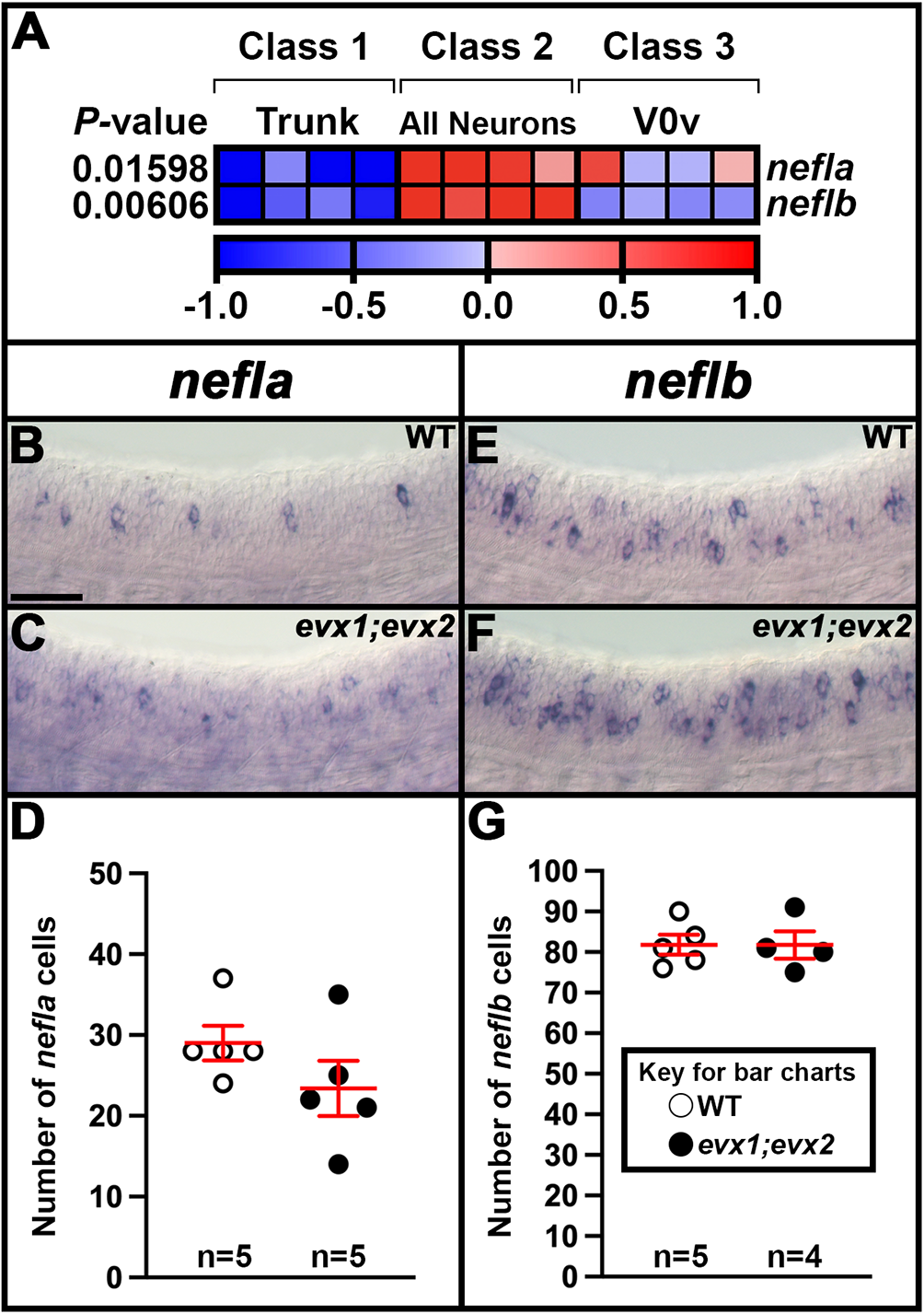

Supplement: Supplementary file 2 — Additional file 2: Supplementary Figure 2.nefla and neflb are Not Expressed in V0v Spinal Interneurons. (A) Heatmap ANOVA analysis of nefla and neflb expression in different FAC-sorted populations of cells. Class 1: All trunk cells. Class 2: All post-mitotic spinal neurons. Class 3: V0v interneurons. Each column is a different biological replicate. Rows show relative expression levels for the gene in question as normalized data transformed to a mean of 0, with standard deviation of +1 (highly expressed, red) or -1 (weakly/not expressed, blue) sigma units. P-values (left-hand side) are corrected for multiple testing. nefla and neflb are expressed in some post-mitotic spinal interneurons (Class 2) but not in V0v spinal interneurons (Class 3). (B, C, E, F) Lateral views of (B, E) WT and (C, F) evx1i232;i232;evx2sa140;sa140 double mutant embryos (labeled evx1;evx2) at 30 h. Rostral, left. Dorsal, up. (D, G) Number of cells expressing (D) nefla and (G) neflb in a precisely-defined spinal cord region adjacent to somites 6-10 at 30 h. Data are depicted as individual value plots and n-values are shown below. For each plot, the wider red horizontal bar depicts the mean number of cells, and the red vertical bar depicts the S.E.M. (these values are listed in Table 1). All counts are an average of at least four embryos. White circles indicate WT data and black circles indicate evx1;evx2 double mutant data. All data were analyzed for normality using the Shapiro-Wilk test. Data sets in D and G are normally distributed and so the F-test for equal variances was performed, followed by a type 2 Student’s t-test (for equal variances). P-values are provided in Table 1. (D, G) There is no statistically significant difference in the number of spinal interneurons expressing either (D) nefla or (G) neflb in evx1;evx2 double mutant embryos, compared to WT embryos. Scale bar: 50 µm. [file 13064_2023_176_MOESM2_ESM.tif]

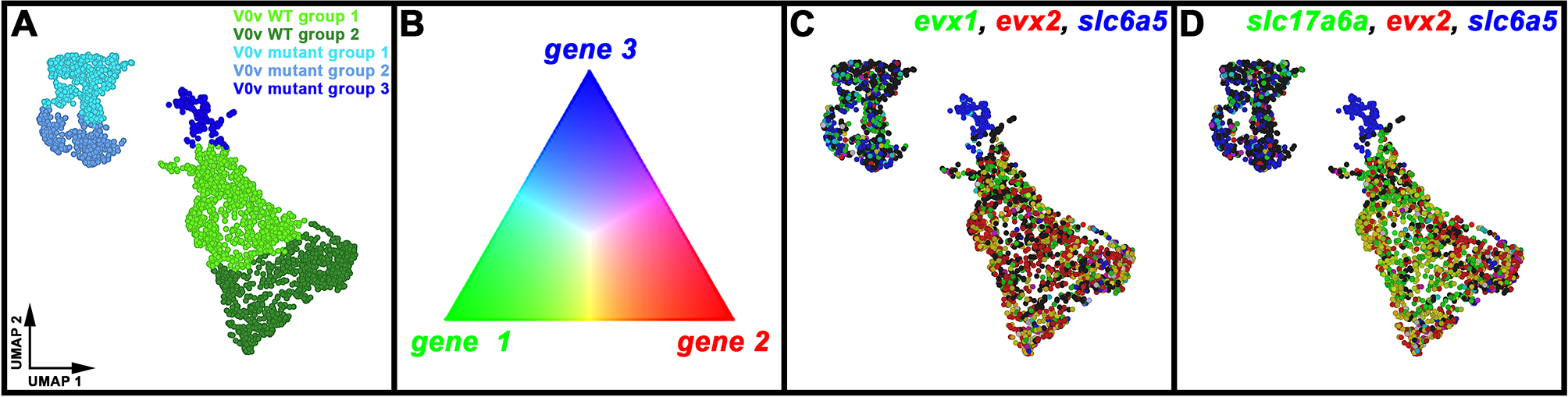

Supplement: Supplementary file 3 — Additional file 3: Supplementary Figure 3. Three-way Differential Gene Expression of WT and evx1/2 Mutant V0v Interneurons. (A) For ease of cell type comparison, panel Supp. Fig. 3A has been reproduced from Fig. 6A. 2D UMAP plot of 48 h post-mitotic V0v spinal interneuron single-cell RNA-seq atlas (2860 cells). Cells were obtained from 48 h embryos produced from an incross of evx1i232/+;evx2sa140/+ heterozygous parents homozygous for Tg(evx1:EGFP)SU2. Clusters are color-coded by cell identity: V0v WT Group 1 (light green), V0v WT Group 2 (dark green), V0v Mutant Group 1 (turquoise), V0v Mutant Group 2 (light blue), and V0v Mutant Group 3 (dark blue). Panel (B) indicates the colour-coding for panels (C-D). This has been reproduced from Fig. 8S. (C-D) Cells expressing only gene 1 are green. Cells expressing only gene 2 are red. Cells expressing only gene 3 are blue. Cells are yellow, pink, or turquoise if they co-express genes 1 and 2, genes 2 and 3, and genes 1 and 3 respectively. Cells expressing all three genes are white. Black shows cells with no expression detected for all three genes of interest. All expression data have been normalization (see Methods). (C) A very small subset of WT cells co-express evx1, evx2 and the glycinergic inhibitory marker slc6a5 (white cells). (D) Similarly, a very small subset of WT cells co-express markers of both glutamatergic excitatory (slc17a6a) and glycinergic inhibitory (slc6a5) phenotypes, together with evx2 (white cells). [file 13064_2023_176_MOESM3_ESM.tif]

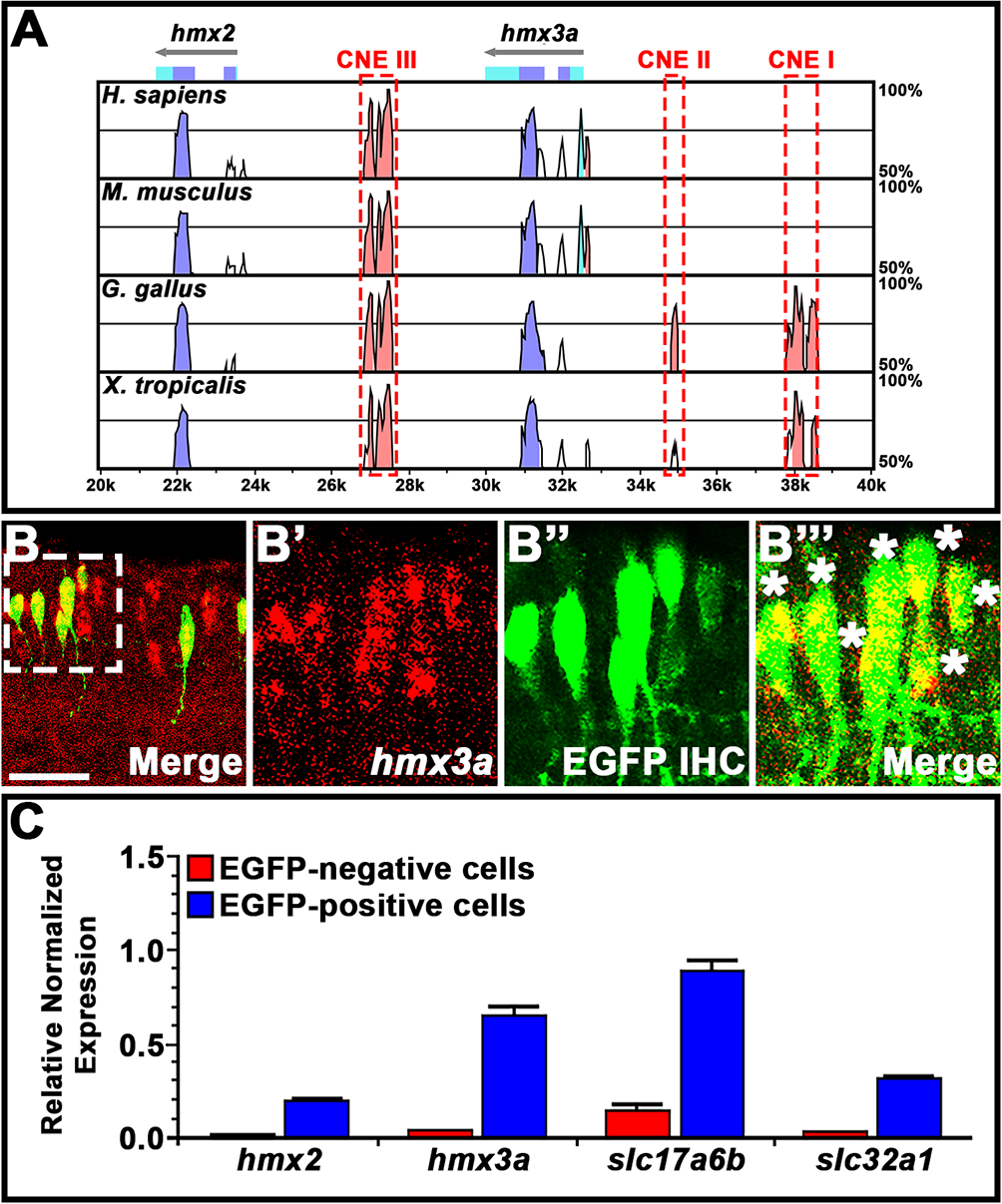

Supplement: Supplementary file 4 — Additional file 4: Supplementary Figure 4. Tg(hmx CNEIII:cos:Gal4-VP16,UAS:EGFP)SU41 Recapitulates Endogenous hmx3a Expression in the Zebrafish Spinal Cord. (A) Schematic showing Shuffle-LAGAN analysis of the contiguous hmx3a-hmx2 genomic region with zebrafish sequence as the baseline, compared to orthologous genomic regions in H. sapiens (row 1), M. musculus (row 2), G. gallus (row 3), and X. tropicalis (row 4). Conserved exonic coding sequences are shown in dark blue. Conserved exonic untranslated sequences are shown in light blue. Grey arrows indicate 5’-3’ orientation. Conserved Non-coding Elements (CNEs) upstream of hmx3a (CNE I and II), and intergenic between hmx3a and hmx2 (CNE III), are shown in pink. The genomic amplicons used for transgenic testing are indicated with red dotted boxes. Only the transgenic line created with CNE III (Tg(hmx CNEIII:cos:Gal4-VP16,UAS:EGFP)SU41) showed EGFP expression in the spinal cord similar to endogenous hmx3a expression (see Methods). This line was used for the experiments described in this paper. (B-B’’’) Lateral views of WT spinal cord at 27 h. Rostral, left. Dorsal, up. (B’) in situ hybridization for hmx3a is shown in red. (B’’) Immunohistochemistry for Tg(hmx CNEIII:cos:Gal4-VP16,UAS:EGFP)SU41 is shown in green. (B, B’’’) Merged images. (B) maximum intensity projection image. (B’-B’’’) high-magnification single confocal planes of the region indicated by white dotted box in B. In zebrafish spinal cord, hmx3a mRNA is exclusively expressed by V1 and dI2 interneurons (12)). (B’’’) All hmx3a-expressing spinal interneurons co-express Tg(hmx CNEIII:cfos:Gal4:UAS:EGFP)SU41 (white asterisks). (C) Quantitative RT-PCR indicates that hmx2, hmx3a, slc17a6b and slc32a1 expression is enriched in Tg(hmx CNEIII:cfos:Gal4-VP16,UAS:EGFP)SU41-expressing cells (blue) compared to non-EGFP-expressing cells (red). Cells were isolated via FACS from 27 h transgenic embryos. This data further suggests that Tg(hmx CNEIII:cos:Gal4-VP16,UAS:EGFP)SU4 [file 13064_2023_176_MOESM4_ESM.tif]
