# Supplementary material for: Molecular analyses of zebrafish V0v spinal interneurons and identification of transcriptional regulators downstream of Evx1 and Evx2 in these cells
Source: Neural Dev. 2023 Nov 28;18:8. doi: 10.1186/s13064-023-00176-w (PMC10683209; doi:10.1186/s13064-023-00176-w)
Supplement: Supplementary file 5 — Additional file 5: Supplementary Table 1. Gene Names, Previous Names, ZFIN Identifiers, Primer Sequences and References for in situ Hybridization Probes. Column 1 lists genes used in this study. Previous names, where known, are provided in column 2. Column 3 contains the unique ZFIN identification number for each gene. Columns 4-6, where relevant, show the primer sequences and expected product sizes (in base pairs (bp)) respectively, used to generate templates for anti-sense RNA riboprobe synthesis from 27 h WT cDNA and the annealing temperature used in the polymerase chain reaction. For further conditions for riboprobe synthesis, please see Methods. Column 7 provides the reference for the in situ hybridization RNA riboprobe used in our experiments. [file 13064_2023_176_MOESM5_ESM.docx]

**Supplementary Table 1. Gene Names, Previous Names, ZFIN Identifiers, Primer Sequences and References for *in situ* Hybridization Probes.**

| **Gene Symbol** | **Previous Names** | **ZFIN ID** | **Primer Sequences** | **PCR Product Size (bp)** | **Annealing Temperature (^o^C)** | **Reference for Probe** |
| --- | --- | --- | --- | --- | --- | --- |
| *ebf3a* | *ebf3, zgc:158829* | ZDB-GENE-070112-292 | Not applicable. | | | Li *et al*., (2010). |
| *ebf3b* | *si:ch211-207n2.6* | ZDB-GENE-131126-3 | FW: CAATAATGGTGTCAGAACAGAGC  RV: **AATTAACCCTCACTAAAGGGA**CCTCTTTTGGCAGTCTTTCC | 761 | 62.0 | This publication – see Methods. |
| *evx1* | *cb416* | ZDB-GENE-980526-364 | Not applicable. | | | Thaëron *et al*., (2000). |
| *evx2* | None | ZDB-GENE-980526-215 | Not applicable. | | | Sordino *et al*., (1996). |
| *hmx3a* | *hmx3, Nkx5-1, nkx5.1, zgc:109845* | ZDB-GENE-001020-1 | Not applicable. | | | Feng & Xu (2010). |
| *inaa* | *si:ch211-67n3.6* | ZDB-GENE-060531-65 | FW: GAGGAGGTCAACGACTACCG  RV: **AATTAACCCTCACTAAAGGGA**AATATGCCATGGTGCTTACC | 846 | 64.0 | This publication – see Methods. |
| *inab* | *ina, etID309796.1, gefiltin, zgc:110516* | ZDB-GENE-990415-83 | FW: TGCTTACAGGAAGCTGTTGG  RV: **AATTAACCCTCACTAAAGGGA**AATAAATGCTTTGCCGTTGC | 702 | 63.0 | This publication – see Methods. |
| *nefla* | *si:ch211-222n4.10* | ZDB-GENE-091117-1 | FW: CTCTAGCCGCCTCCACTATG  RV: **AATTAACCCTCACTAAAGGGA**AACCTCCAACTCCCACACTG | 1091 | 64.0 | This publication – see Methods. |
| *neflb* | *nefl, zgc:136626* | ZDB-GENE-060312-44 | FW: TGTTTTCTCCCTCCATTGCT  RV: **AATTAACCCTCACTAAAGGGA**GGCATACTGACACAATGCTCA | 812 | 62.0 | This publication – see Methods. |
| *nefma* | *zgc:112359* | ZDB-GENE-050522-205 | FW: CACACCGCACCTGTACACTAC  RV: **AATTAACCCTCACTAAAGGGA**CGGTCTTCTATGTCGTTCAGC | 1075 | 65.0 | This publication – see Methods. |
| *nefmb* | *nefm, zgc:158491* | ZDB-GENE-070103-4 | FW: CGAAAAGCAAGTCGACAAGG  RV: **AATTAACCCTCACTAAAGGGA**TTGAGGTGATATTGCAAAGAGC | 740 | 62.0 | This publication – see Methods. |
| *skor1a* | *zgc:194563* | ZDB-GENE-080722-5 | FW: ACTATCCGGTCATCCCTGTG  RV: **GCAATTAACCCTCACTAAAGGGA**GAGGTGGCTTAAAGGTGCAG | 1374 | 64.0 | This publication – see Methods. |
| *skor1b* | *si:dkey-246a16.3* | ZDB-GENE-050419-82 | FW: CGAGAGCAGGTCGATAGAGG  RV: **GCAATTAACCCTCACTAAAGGGA**GAGGTTTGAAGGTGCAGGTC | 968 | 64.0 | This publication – see Methods. |
| *skor2* | *skor2, zgc:153395* | ZDB-GENE-060825-57 | FW: CGCAAAGACGCTTTTTATCC  RV: **AATTAACCCTCACTAAAGGGA**AAATGGAGAGCTGCCTTTCAG | 1026 | 61.0 | This publication – see Methods. |
| *uncx* | None | ZDB-GENE-080509-1 | FW: CAAGGGTGCAGGTATGGTTTC  RV: **AATTAACCCTCACTAAAGGGA**CTGGTTGTCGCTGATTCTGAATTTTG | 985 | 64.0 | This publication – see Methods. |
| *uncx4.1* | None | ZDB-GENE-050714-1 | FW: AAATAGCACGACGGGAGTTG  RV: **AATTAACCCTCACTAAAGGGA**CAAGTGAGCAGAGCCACTGA | 1051 | 63.0 | This publication – see Methods. |
| *neff1* | *cb126, ina, sb:cb126; zgc:65851* | ZDB-GENE-030131-9669 | FW: GAGCGATAGCAAGGCAGATAGC  RV: **AATTAACCCTCACTAAAGGGA**CTGGAATACAGAATCACATGGCCG | 463 | 66.0 | This publication – see Methods. |

References

Feng Y, Xu Q. Pivotal role of hmx2 and hmx3 in zebrafish inner ear and lateral line development. Dev Biol. 2010;339(2):507-18.

Li S, Yin M, Liu S, Chen Y, Yin Y, Liu T, et al. Expression of ventral diencephalon-enriched genes in zebrafish. Developmental dynamics: an official publication of the American Association of Anatomists. 2010;239(12):3368-79.

Sordino P, Duboule D, Kondo T. Zebrafish *Hoxa* and *Evx-2* genes: cloning, developmental expression and implications for the functional evolution of posterior *Hox* genes. Mech Dev. 1996;59:165 – 75.

Thaëron C, Avaron F, Casane D, Borday V, Thisse B, Thisse C, et al. Zebrafish evx1 is dynamically expressed during embryogenesis in subsets of interneurones, posterior gut and urogenital system. Mechanisms of development. 2000;99(1-2):167-72.
